# A dynamic social relations model for clustered longitudinal dyadic data with continuous or ordinal responses

**DOI:** 10.1093/jrsssa/qnad115

**Published:** 2023-09-05

**Authors:** Rebecca Pillinger, Fiona Steele, George Leckie, Jennifer Jenkins

**Affiliations:** Independent Researcher, Edinburgh, UK; Department of Statistics, London School of Economics & Political Science, London, UK; School of Education, University of Bristol, Bristol, UK; Applied Psychology and Human Development, University of Toronto, Toronto, Canada

**Keywords:** autoregressive model, cross-lagged effects, dyadic data, dynamic panel model, round-robin data

## Abstract

Social relations models allow the identification of cluster, actor, partner, and relationship effects when analysing clustered dyadic data on interactions between individuals or other units of analysis. We propose an extension of this model which handles longitudinal data and incorporates dynamic structure, where the response may be continuous, binary, or ordinal. This allows the disentangling of the relationship effects from temporal fluctuation and measurement error and the investigation of whether individuals respond to their partner’s behaviour at the previous observation. We motivate and illustrate the model with an application to Canadian data on pairs of individuals within families observed working together on a conflict discussion task.

## 1 Introduction

Data on pairs of subjects or dyads are commonly collected in social research, for example in studies of consensus in individuals’ perceptions of a common target (such as spouses’ assessment of the quality of their relationship) or in studies of interactions between individuals (such as cooperation between coworkers). The prototypical design for collecting dyadic data is a round-robin design in which each member of a group of subjects rates or interacts with every other member. The resulting directed relationship data exhibit a complex correlational structure as each person is a member of multiple dyads. Furthermore, each dyad contributes two responses, one associated with each dyad member in their distinct roles as ‘actor’ and ‘partner’ in their two-way ratings or interactions. The social relations model (SRM) is widely used to analyse dyadic data from round-robin designs. The SRM decomposes the overall variance in the directed dyadic measurements into separate variance components attributable to individuals as actors, individuals as partners, and unique interaction (relationship) variance. The SRM also provides detailed analysis of reciprocity both at the individual level (referred to as generalised reciprocity correlation) and at the level of unique relationships (dyadic reciprocity correlation). This allows one to answer the questions: Do individuals who in general show high outcome behaviour in their roles as actors in their interactions also in general experience high outcome behaviour from others in their roles as partners? and Within dyads, is atypical outcome behaviour reciprocated, either positively or negatively?

Variants on the basic SRM that are of particular relevance to this article are models for clustered data where individuals within clusters have distinct roles (Kashy & Kenny, 1990; Snijders & Kenny, 1999). We present an application to data on constructiveness from family members working on a task in pairs in a round-robin design where the individuals within families are distinguishable by their roles (mother, father, child, with children potentially further differentiated by birth order) and separate actor, partner, and relationship variances and reciprocity correlations are specified by role or pairing of roles. Jenkins et al. (2012) study informant and partner influences among siblings on their ratings of sibling affection and hostility. Browne et al. (2016) study cognitive sensitivity exhibited between family members on cooperative tasks and show the relative importance of family, individuals, and unique relationships vary substantially by role. The findings from studies such as these have been that individuals do show consistency across family relationships in terms of the behaviours that they direct to others (the actor effect) and to a lesser extent the behaviours that they elicit from others (the partner effect) but in general these effects are substantially smaller than those attributable to the relationship. Thus the important theoretical conclusion from this area of research has been that the combination of people in the dyad is more important to understand relationship quality in families than the individuals themselves. However, a limitation of all these cross-sectional studies is that individual behaviours are measured only once and so the relationship effect is confounded with and therefore inflated by measurement error.

There have been very few examples of longitudinal round-robin data where individual behaviours are measured repeatedly over time. Yet such data have a number of advantages. First, intensive longitudinal data, because of the separation of time-invariant and time-varying processes, have greater potential to isolate different types of interpersonal influence. The non-dynamic SRM allows for the identification of a partner effect. This represents a particular type of interpersonal influence: an individual consistently elicits the same response from multiple interactional partners (e.g. a person elicits a high level of hostility from all partners). The dynamic SRM, through modelling the time-varying behaviour of each individual, allows us to identify another type of interpersonal influence: whether one person’s prior behaviour predicts another person’s subsequent behaviour. We refer to this as a ‘proximal trigger’.

Separating the time-invariant from the time-varying also allows the opportunity to properly examine time-invariant processes, having removed both temporal fluctuation and measurement error. A theoretically important question for dyadic research is to estimate the different contributions from the stabilities of individuals (actors and partners), the unique combination of a dyad (relationship variance), and the unexplained temporal fluctuation (i.e. the combined effects of potentially meaningful fluctuation in behaviour and measurement error, which we together refer to as ‘residual error’). Yet previous SRMs confound the relationship variance and residual error, with the result that researchers overstate the importance of relationship effects in driving dyadic interactions, understate the importance of family and individual effects (actor and partner), and have nothing to say about temporal fluctuation (some of which may be explained by proximal triggers). Correctly assigning these components of variation additionally enables accurate assessment of how they, and the average level of the outcome variable, differ according to explanatory variables. For instance, do adults show less hostility than children? Understanding time-invariant influences on behaviour (e.g. personality and age-related biological maturation) have been core theoretical issues in social and developmental psychology.

Analysing longitudinal dyadic data with a model that combines the key features of the SRM for cross-sectional dyadic data with methods for multivariate response longitudinal data allows for a richer exploration of the ways in which individuals affect one another. In this paper we propose a dynamic SRM for repeated measures data from a clustered round-robin design where individuals have specific roles. The model can be viewed as a type of cross-classified multilevel (random effects) model for bivariate longitudinal data with a first-order autocorrelation and cross-lagged structure on the within-dyad residual correlation matrix. We show that the dynamic SRM can also be framed as an extension of an autoregressive cross-lagged panel model (or vector autoregressive model). A generalisation suitable for binary and ordinal responses is developed and applied in an analysis of family, actor, partner, and relationship effects on the observed behaviour by one family member towards another in a conflict discussion task carried out by a sample of 7-year old Canadian children and their families. Model estimation can be carried out using software for Bayesian analysis using Markov chain Monte Carlo (MCMC) methods.

The rest of the paper is organised as follows. An overview of the SRM and other approaches to the analysis of dyadic data is given in Section 2. In Section 3, we describe a cross-sectional SRM for clustered round-robin data in order to fix notation, terminology, and the interpretation of model parameters. Section 4 shows how this model can be extended for the analysis of longitudinal data with a first-order autoregressive and cross-lagged dynamic structure. Then, a generalisation of this model, referred to as a latent dynamic SRM, is described for binary and ordinal responses. Section 5 describes the data we use from the Kids, Families and Places study, a birth cohort study of Canadian children and their families. Section 6 sets out the research questions to be investigated using the dynamic SRM (and various simplifications) and provides results from the data analysis. Section 7 concludes with a summary of the main findings and a discussion of potential model extensions.

## 2 Overview of the SRM and other approaches to the analysis of dyadic data

The SRM was proposed by David Kenny and colleagues in psychology to study interpersonal perception and behaviours (Kenny et al., 2006; Kenny & La Voie, 1984). Application of the SRM has since expanded to incorporate a broad range of topics, including geography (studies of migration flows between countries; Zhang et al., 2020), international relations and economics (militarised interstate disputes and international trade; Dorff & Ward, 2013), anthropology and behavioural ecology (studies of food sharing among indigenous Nicaraguan horticulturalists; Koster & Leckie, 2014), law (studies of jurors influencing each other on jury verdicts; Marcus et al., 2000), education (reports of bullying among classmates within school classes; Veenstra et al., 2007), and management (ratings of deference shown towards coworkers within scientific research teams; Joshi & Knight, 2015).

In the classic round-robin design each individual *i* is paired with every other individual *j*, leading to two directed responses for each undirected dyad, for example, the behaviour of *i* (the ‘actor’) towards *j* (the ‘partner’) and the behaviour of *j* towards *i* where the actor–partner roles are swapped. (The terms actor and partner may be replaced by others such as perceiver and target, sender and receiver, or rater and subject according to the application.) The pairing of each individual with every other individual (or in some applications a subset of the others) allows three types of effect to be estimated: an individual’s actor effect (shared by all dyads with the same actor), an individual’s partner effect (shared by all dyads with the same partner), and a relationship effect (specific to each unique directed actor–partner combination). Interest lies in estimating the components of variance in the response that are attributable to these three effects, and in the correlation between an individual’s actor and partner effects (referred to as generalised reciprocity) and between the two relationship effects for a given dyad (dyadic reciprocity).

The SRM was originally formulated for single-group designs where both individuals and dyads are non-differentiated or exchangeable (Kenny & La Voie, 1984). More generally, dyads are formed within clusters such as families or organisations (Kenny et al., 2006) and individuals may have different roles such as parent and child in families or employee and manager in the workplace. In such designs, cluster effects are of interest and the mean response, variance decomposition, and reciprocity correlations for a dyad *ij* may depend on the roles of individuals *i* and *j*. Kashy and Kenny (1990) described an extended SRM for family designs where all parameters are role-specific and showed how the model can be framed as a structural equation model. An alternative approach, proposed by Snijders and Kenny (1999), is to view the SRM as a type of cross-classified multilevel (random effects) model where actor and partner effects are crossed because each actor is paired with multiple partners, and vice versa. A complication arises in that, rather than making the usual assumption that the different sets of random effects are independent, the actor and partner random effects must be allowed to correlate in order to capture the generalised reciprocity correlation. Similarly, the residual terms must be allowed to correlate within dyads to capture the dyadic reciprocity correlation. An important advantage of the multilevel framework is the straightforward generalisation of the SRM to handle cluster effects in *k*-cluster designs where clusters may vary in size (Snijders & Kenny, 1999).

Among the few studies that have considered SRMs for longitudinal round-robin data, the most common approaches for clustered data are based on cross-sectional models fitted separately to each time point. Nestler et al. (2017) review two-stage methods where estimates of actor and partner effects from a series of cross-sectional SRMs are used as response variables in longitudinal analyses of changes in these effects over time. As noted by Nestler et al., two-stage approaches are problematic because they ignore uncertainty in the estimation of actor and partner effects and their correlation. An alternative approach described by Nestler et al. is to use a multivariate response SRM, employed in previous research for multiple cross-sectional responses (Card et al., 2008), treating repeated measures as a multivariate response for each directed dyad *ij* and estimating an unstructured covariance matrix. While a multivariate SRM fully captures within undirected dyad covariances, the lack of structure leads to a large number of random effect parameters. This would especially be the case in our application to family data where all parameters may be role-specific.

A restricted form of the full multivariate model is the social relations growth model (SRGM) (Nestler et al., 2017) in which random effects for the intercept and a linear time effect are decomposed into actor, partner, and dyad effects. The SRGM is appropriate for developmental processes where there is interest in the relative contributions of actor, partner, and dyad effects to the mean of *y* at baseline (the intercept) and to changes in *y* over time (the slope), and correlations among the different intercept and slope effects. For example, Nestler et al. apply the SRGM to study changes in how much freshmen at a US university liked each other over time after their first meeting. Moreover, given the complexity of the covariance structure and large number of parameters to interpret, the SRGM is most suited to applications where change can be represented by a linear function of time and to exchangeable round-robin designs (i.e. without role effects).

Another approach to the analysis of dyadic data is a stochastic block model (SBM). In an SBM, each node in a network is assumed to belong to one (or possibly more) of *K* latent blocks and the objective is to discover the community structure of the network through identification of blocks of highly connected nodes (Airoldi et al., 2008; Holland et al., 1983). SBMs have been generalised to handle longitudinal network data where block membership may be time-varying and the evolution of a network over time is studied using a form of hidden Markov model in which block membership at time *t* depends on membership at t−1 (Bartolucci et al., 2018; Matias & Miele, 2017). SBMs are typically applied to network data where ties may exist between any pair of the observed nodes and the aim is to model the probability of a tie. While SRMs can also be used in this situation, and models that combine an SRM and an SBM have recently been proposed for cross-sectional data of this type (Redhead et al., 2023), the focus of this paper is the analysis of longitudinal network data from a round-robin design. In a blocked or clustered round-robin design, interest lies in interactions between nodes, rather than ties; these interactions are confined to members of the same block and block membership is known and time-invariant. In our application, for example, families are ‘blocks’ and the discrete latent variable for blocks in an SBM is instead represented by a continuous cluster-specific random effect.

## 3 SRM for clustered cross-sectional round-robin data

We begin with a description of an SRM for clustered round-robin data in the context of a family study with two parents and two children, although it is straightforward to accommodate mixed family sizes (e.g. single-parent or one-child families). Suppose that each pair of family members is observed in an interaction and the response of interest is the behaviour of one individual in a pair towards the other. Individuals are distinguished by their role in the family: mother (M), father (F), younger child/sibling ($S_{1}$), and older child ($S_{2}$). Dyads are also distinguished by the respective roles of the actor and partner. Denote by $y_{ijk}$ the behaviour of actor *i* towards partner *j* in family *k*, where i,j=1,…,4 (i≠j) index the family roles (M, F, $S_{1},S_{2}$), and k=1,…,K. For each pair of family members, we observe the bivariate response $\left(y_{ijk},y_{\;jik}\right)$, and within a family there are up to 12 directed dyads (corresponding to each actor–partner combination) and 6 undirected dyads (corresponding to each pair of individuals without specifying one as the actor and the other as the partner).

Following Snijders and Kenny (1999), a linear model for $y_{ijk}$ can be written:


(1) $$y_{ijk}=\mu_{ij}+f_{k}+a_{ik}+p_{\;jk}+d_{ijk}\;\text{ for}\;i\neq j$$

where $\mu_{ij}$ is the mean, which varies across directed dyads, $f_{k}$ is the family effect, $a_{ik}$ is the actor effect for individual *i* in family *k*, $p_{\;jk}$ is the partner effect for individual *j* in family *k*, and $d_{ijk}$ is the relationship or dyad effect unique to the directed dyad *ij* in family *k*. The following univariate and bivariate distributions are assumed for the random effects, allowing the actor, partner, and relationship variances and covariances to depend on family roles (hence the inclusion of *i* and *j* subscripts on these parameters).


(2) $$\begin{array}{rl} \;f_{k} & ∼N(0,\sigma_{\;f}^{2})\\ \left[\begin{array}{c} a_{ik}\\ p_{ik} \end{array}\right] & ∼N\left(\left[\begin{array}{c} 0\\ 0 \end{array}\right],\left[\begin{array}{cc} \sigma_{ai}^{2}\\ \rho_{api}\sigma_{ai}\sigma_{\;pi} & \sigma_{\;pi}^{2} \end{array}\right]\right)\\ \left[\begin{array}{c} d_{ijk}\\ d_{\;jik} \end{array}\right] & ∼N\left(\left[\begin{array}{c} 0\\ 0 \end{array}\right],\left[\begin{array}{cc} \sigma_{dij}^{2}\\ \rho_{dij}\sigma_{dij}\sigma_{dji} & \sigma_{dji}^{2} \end{array}\right]\right) \end{array}$$

The four $\rho_{ap}$ parameters are the role-specific generalised reciprocity correlations, i.e. the correlations between the same individual’s actor and partner effects, and measure the relationship between an individual’s tendency to act in a certain way towards all other family members and their tendency to elicit a certain behaviour from all other family members. The six $\rho_{d}$ parameters are dyad-level correlations between relationship effects, referred to as dyadic reciprocity; these are permitted to vary across the six undirected dyad types with $\rho_{dij}=\rho_{dji}$. These correlations measure the extent to which unusual behaviour within dyads, that is, behaviour unaccounted for by the two family members’ general actor and partner tendencies, is positively or negatively reciprocated.

The total variance of $y_{ijk}$ can thus be partitioned as


(3) $$\mathrm{var}(y_{ijk})=\sigma_{\;f}^{2}+\sigma_{ai}^{2}+\sigma_{\;pj}^{2}+\sigma_{dij}^{2}$$

where the variance components are respectively the between family variance, and the between-actor, between-partner, and between relationship variances across families.

While the model can be estimated using maximum likelihood or iterative generalised least squares (Goldstein, 2010; Snijders & Kenny, 1999), Bayesian methods are more computationally efficient and flexible for estimation of cross-classified and other types of non-hierarchical multilevel model (Browne et al., 2001; Gill & Swartz, 2001), and extensions to generalised linear models for discrete response data (Hoff, 2005; Koster & Leckie, 2014) and latent variable models for multivariate data (Gin et al., 2020).

## 4 Dynamic SRM for clustered longitudinal round-robin data

### 4.1 Longitudinal SRM with dynamic effects

Our research questions concern the dynamics in dyadic interactions, that is, the persistence in an individual’s behaviour over the course of an interaction and the moment-to-moment influences of each undirected dyad member’s behaviour on the other’s. Also of interest is the extent to which such dynamics vary according to individual roles within the family and the respective roles of the actor and partner within a dyad. We therefore propose a dynamic SRM which has two components: an SRM for the repeated measures of *y* and a dynamic (autoregressive and cross-lagged) structure for the time-varying residuals.

The SRM component of the model is a simple extension of (1) with an extra subscript *t* to denote the timing of each measurement of *y* and the addition of a time-varying residual $e_{tijk}$ to capture temporal fluctuations (and measurement error) in the behaviour shown between family members (i.e. the state):


(4) $$\begin{array}{rl} y_{tijk} & =\mu_{ij}+f_{k}+a_{ik}+p_{\;jk}+d_{ijk}+e_{tijk}\;\text{ for}\;i\neq j\\ \left[\begin{array}{c} e_{tijk}\\ e_{tjik} \end{array}\right] & ∼N\left(\left[\begin{array}{c} 0\\ 0 \end{array}\right],\left[\begin{array}{cc} \sigma_{eij}^{2}\\ \rho_{eij}\sigma_{eij}\sigma_{eji} & \sigma_{eji}^{2} \end{array}\right]\right) \end{array}$$

where the distributions of the random effects $f_{k}$, $a_{ik}$, $p_{\;jk}$, and $d_{ijk}$ are the same as in (2) for the cross-sectional case.

The intercepts $\mu_{ij}$ are the coefficients of 12 dummy variables, one for each combination of the family roles of the actor and partner. It is straightforward to extend (4) to include other covariates which may be time-invariant or time-varying characteristics of individuals, dyads, and families. We do not consider covariates in our application because our focus is on the unconditional autoregressive and cross-lagged effects, random effect variances and correlations, and variance partitioning coefficients (VPCs). However, we consider a dyad-specific linear trend by replacing $\mu_{ij}$ with $\beta_{0ij}+\beta_{1ij}t$ in a special case of the social relations growth model of Nestler et al. (2017).

We extend the model given by (4) and (2) to allow for autoregressive and cross-lagged correlation among the within-dyad deviations $e_{tijk}=y_{tijk}-\theta_{ijk}$, where $\theta_{ijk}=\mu_{ij}+f_{k}+a_{ik}+p_{\;jk}+d_{ijk}$ is the linear predictor: an individual’s mean response over the entire period of the interaction in a particular dyad. Specifically we consider a first-order model where the deviation in the response for actor *i* towards partner *j* at time *t* from their mean response depends on their own lagged deviation one occasion ago, $e_{(t-1)ijk}$, and that of their partner, $e_{(t-1)jik}$. In other words, we allow an individual’s deviation from their long-run average behaviour towards any particular partner to be correlated with their corresponding deviation in long-run average behaviour towards the same partner at the previous measurement occasion (an autoregressive effect), and to be affected by that partner’s unique behaviour towards them at the previous measurement occasion (a cross-lagged effect). Including an autoregressive effect allows us to test the hypothesis that individuals’ unique behaviours exhibit some degree of persistence, while a cross-lagged effect tests the hypothesis that individuals will react to the behaviour shown to them and reciprocate in kind (or alternatively, respond with opposite behaviour), i.e. that an individual’s behaviour towards another influences the second individual’s behaviour towards the first.

The within-dyad model can therefore be written as


(5) $$e_{tijk}=\phi_{1ij}e_{(t-1)ijk}+\phi_{2ij}e_{(t-1)jik}+\eta_{tijk}\;\text{ for}\;t>1$$

where $\phi_{1ij}$ and $\phi_{2ij}$ are the autoregressive and cross-lagged effects which vary across directed dyads. The residuals $\eta_{tijk}$, which capture both temporal fluctuation not accounted for by the autoregressive and cross-lagged effects and measurement error, are sometimes referred to as shocks or innovations and are assumed to have a bivariate normal distribution


(6) $$\left[\begin{array}{c} \eta_{tijk}\\ \eta_{tjik} \end{array}\right]∼N\left(\left[\begin{array}{c} 0\\ 0 \end{array}\right],\left[\begin{array}{c} \sigma_{\eta ij}^{2}\\ \rho_{\eta ij}\sigma_{\eta ij}\sigma_{\eta ji}\sigma_{\eta ji}^{2} \end{array}\right]\right)$$

where $\rho_{\eta ij}=\rho_{\eta ji}$ but we allow $\sigma_{\eta ijk}^{2}\neq \sigma_{\eta jik}^{2}$. To complete the model specification, we assume that the residuals for t=1, $\left(e_{1ijk},e_{1jik}\right)$, also have a bivariate normal distribution but with a different covariance matrix to $\left(\eta_{tijk},\eta_{tjik}\right)$ for t>1.

Usami et al. (2019) review a range of cross-lagged panel models for longitudinal bivariate data of the form $y_{ti}=(y_{1ti},y_{2ti})$ where the two responses at each time *t* are observed on the same unit *i* (e.g. individual). Such data have a two-level hierarchical structure with repeated measures of the bivariate response nested within individuals. One of the models reviewed by Usami et al. is a random intercept model which includes (uncorrelated) response-specific random effects $\left(u_{1i},u_{2i}\right)$ to allow for unmeasured time-invariant influences on each response. The model specified by (4) and (5) generalises this model for the case of longitudinal dyadic data, a particular kind of bivariate data that have a more complex non-hierarchical structure, as described above; moreover, dyads are nested within family clusters. Thus the response-specific random intercepts are replaced by the linear combination of family, individual (actor and partner), and dyad random effects given by (4), allowing additionally for the random effect variances and covariances to be role-specific. In a random coefficients extension of the random intercept model of Usami et al., Hamaker et al. (2018) allow the autoregressive and cross-lagged effects in (5) to vary randomly across individuals. However, we restrict our attention to fixed dyad-type-specific effects due to the complex structure of our data and joint modelling of multiple dyads within families.

An alternative way to incorporate dynamic effects is to allow for autoregressive and cross-lagged effects among the responses $y_{tijk}$ rather than among the residuals. For standard bivariate panel data (on independent subjects rather than clustered dyads), such a model is known in psychometrics as an autoregressive cross-lagged model (Bollen & Curran, 2006) and in econometrics as a random effects panel vector autoregressive model (Binder et al., 2005). However, by generalising a result from Anderson and Hsiao (1981) for univariate repeated models, it can be shown that the two specifications are equivalent. To see how the model of (5) can be reformulated as an SRM generalisation of an autoregressive cross-lagged model, we substitute $e_{tijk}=y_{tijk}-\theta_{ijk}$ in (5) to obtain


(7) $$y_{tijk}={\tilde{\theta }}_{ijk}+\phi_{1ij}y_{(t-1)ijk}+\phi_{2ij}y_{(t-1)jik}+\eta_{tijk}\;\text{ for}\;t>1$$

where ${\tilde{\theta }}_{ijk}=(1-\phi_{1ij})\theta_{ijk}-\phi_{2ij}\theta_{\;jik}$. Since the coefficient of $y_{(t-1)ijk}$ in this parameterisation is identical with the coefficient of $e_{(t-1)ijk}$ in the original parameterisation given by (4) and (5), we can interpret $\phi_{1ij}$ equally well as the effect of the actor’s response at the previous occasion on their current response or as the effect of the deviation in their response at the previous occasion from their average when acting towards that partner on the deviation in their response at the current occasion; the former interpretation is more straightforward but the latter corresponds to our conceptualisation of the model as a dynamic structural equation model (SEM). Similarly, we can equally well interpret $\phi_{2ij}$ as the effect of the partner’s response at the previous occasion on the actor’s current response or as the effect of the partner’s deviation in their response at the previous occasion from their average when acting towards this actor on the actor’s deviation in their response at the current occasion from their average when acting towards that partner. Finally, ${\tilde{\theta }}_{ijk}$ can be decomposed as ${\tilde{\theta }}_{ijk}={\tilde{\mu }}_{ij}+{\tilde{\;f}}_{ijk}+{\tilde{a}}_{ijk}+{\tilde{\;p}}_{\;jik}+{\tilde{d}}_{ijk}$ with


(8) $$\begin{array}{rl} {\tilde{\mu }}_{ij} & =(1-\phi_{1ij})\mu_{ij}-\phi_{2ij}\mu_{\;ji}\\ {\tilde{\;f}}_{ijk} & =(1-\phi_{1ij})f_{k}-\phi_{2ij}f_{k}\\ {\tilde{a}}_{ijk} & =(1-\phi_{1ij})a_{ik}-\phi_{2ij}a_{\;jk}\\ {\tilde{\;p}}_{\;jik} & =(1-\phi_{1ij})p_{\;jk}-\phi_{2ij}p_{ik}\\ {\tilde{d}}_{ijk} & =(1-\phi_{1ij})d_{ijk}-\phi_{2ij}d_{\;jik} \end{array}$$

We note that $\left({\tilde{\;f}}_{ijk},{\tilde{a}}_{ijk},{\tilde{\;p}}_{\;jik},{\tilde{d}}_{ijk}\right)$ cannot be interpreted as family, actor, partner, and relationship effects because all vary across both actors and partners within a family. In an alternative specification of a dynamic model for $y_{tijk}$, ${\tilde{\theta }}_{ijk}$ could be replaced by an SRM decomposition, but the variances and covariances of the family, actor, partner, and relationship effects in the resultant model would be conditional on $\left(y_{(t-1)ijk},y_{(t-1)jik}\right)$. We instead focus on the parameterisation given by (4) and (5) where the variance–covariance parameters for $\left(f_{k},a_{ik},p_{\;jk},d_{ijk}\right)$ have an unconditional interpretation. However, we use the parameterisation of (7) and (8) for estimation, as described below.

### 4.2 Latent dynamic SRM for ordinal data

In our application, the response variable is ordinal rather than continuous. We therefore consider a generalisation of the dynamic SRM to handle ordinal responses. We note that binary responses are simply two-category ordinal responses and so this generalisation can equally be applied to binary response variables. The model is specified in terms of an underlying continuous response $y_{tijk}^{*}$, related to an observed ordinal $y_{tijk}$ with *C* categories by


(9) $$y_{tijk}=c\;⟺\;y_{tijk}^{*}\in [\tau_{c-1},\tau_{c}),\;c=1,\ldots ,C$$

where $\tau_{c}$ are threshold parameters with $-\infty =\tau_{0}<\tau_{1}<\cdots <\tau_{C-1}<\tau_{C}=\infty$. The dynamic SRM for ordinal responses can then be written as


(10) $$y_{tijk}^{*}=\mu_{ij}+f_{k}+a_{ik}+p_{\;jk}+d_{ijk}+e_{tijk}\;\text{ for}\;i\neq j$$

For identification, we must fix the location and scale of $y_{tijk}^{*}$. We fix the location of $y_{tijk}^{*}$ by setting $\tau_{1}=0$, and we fix its scale by setting $\mathrm{var}(\eta_{tijk})=1$ in (5) and $\mathrm{var}(e_{1ij})=1$ in (10). Retaining the normality assumption for $\eta_{tijk}$ and $e_{1ijk}$ gives an ordered probit model.

As in the continuous response case, the dynamic SRM can be formulated as an autoregressive cross-lagged model where (7) is expressed in terms of the latent response $y_{tijk}^{*}$ and the lagged and cross-lagged latent responses $y_{(t-1)ijk}^{*}$ and $y_{(t-1)jik}^{*}$. This leads to a bivariate generalisation of the latent autoregression model proposed by Pudney (2008) for a univariate ordinal response.

It is well known in the latent variable modelling literature that the inappropriate treatment of multivariate ordinal data as continuous can lead to biased parameter estimates, especially when *C* is small and for skewed response distributions (Dolan, 1994). For this reason, it is commonly advocated for factor analysis of ordinal data to be based on polychoric correlations, which estimate the associations among multivariate normal $y^{*}$s, rather than Pearson correlations for the manifest ordinal *y*s (e.g. Flora & Curran, 2004). Pearson correlations computed for discrete *y*s underestimate the dependency among the $y^{*}$s, which leads to biased estimates of factor loadings. These findings are relevant for the dynamic SRM proposed in this paper because it is a form of latent variable model with various correlation parameters relating to within-person autocorrelation ($\phi_{1ij}$), and cross-person within-dyad autocorrelation ($\phi_{2ij}$) and the random effect and innovation correlations in (2) and (6).

### 4.3 Estimation

As in the case of the basic cross-sectional SRM and the generalisations noted in Section 3, Bayesian MCMC estimation provides an efficient way of estimating the dynamic SRM, especially in data with many individuals per cluster and for ordinal and other discrete responses. All data analysis presented in Section 6 was carried out using Gibbs sampling, as implemented in the JAGS package (Plummer, 2003). Although the variance components and correlations of interest in the SRM part of the model are the unconditional parameters of (10), it is more convenient for estimation purposes to specify the model in the autoregressive cross-lagged response form of (7) with *y* replaced by $y^{*}$ and the fixed intercept ${\tilde{\mu }}_{ij}$ and the variance–covariance parameters of the random effects $\left({\tilde{\;f}}_{ijk},{\tilde{a}}_{ijk},{\tilde{\;p}}_{\;jik},{\tilde{d}}_{ijk}\right)$ expressed in terms of the parameters of interest using the relationships in (8). An advantage of MCMC estimation is the possibility to derive estimates and credible intervals for functions of the parameters of the fitted model using the parameter chains. (The JAGS code is provided in the online supplementary material.)

In order to ensure the estimates of the innovation correlations lie between −1 and 1, we model the off-diagonal elements of the precision matrix for each undirected dyad with a hyperbolic tangent function (i.e. Fisher’s *z* transformation). We use exponential priors with rate 1 for the gaps between each pair of consecutive thresholds, Normal priors with mean 0 and precision 0.001 for the fixed part parameters, Wishart priors with the 2×2 identity matrix and 2 degrees of freedom for the random part precision matrices (i.e. inverses of the variance–covariance matrices of the actor and partner effects for each role, and of the variance–covariance matrices of the relationship effects for each directed dyad type), a Gamma prior with shape 0.001 and rate 0.001 for the precision of the family effects, and Normal priors with mean 0 and variance 0.001 for the parameters used in modelling the correlations between the innovations.

## 5 Data

Participants in this study were originally recruited as part of the *Kids, Families, and Places* study, a birth cohort longitudinal study that followed children born between February 2006 and February 2008 in Toronto or Hamilton, Canada, into school (Browne et al., 2018; Meunier et al., 2013). This sample was recontacted to take part in a subsequent study, the goals of which were to understand the development of cooperation in family relationships. The data for the current study were drawn from the cooperation study. Participants from 223 families were video recorded working on a conflict discussion task in pairs, and raters subsequently watched the recordings and scored each individual’s constructiveness at 20 s intervals (‘snapshots’). In most families, the participants were the mother (M), father (F), younger child ($S_{1}$), and older child ($S_{2}$), but in 65 families the father did not participate (in some cases because it was a single-parent family and in others because the father did not wish to participate), and in two families one of the children did not participate. Our analysis sample therefore includes observations on either four family members (M, F, $S_{1},S_{2}$) or three family members (M, $S_{1},S_{2}$; M, F, $S_{1}$; or M, F, $S_{2}$). No adjustment is required to handle families of different sizes, although families without a participating father do not contribute to estimates of parameters for dyads involving the father. The children who were newborns at the time of initial recruitment—termed the ‘younger siblings’ or ‘younger children’ in this study—were between 5 and 9 years old when these data were collected (M=7.34; SD=0.82; 51% female). Their next oldest siblings (‘older siblings’ or ‘older children’) were 7–13 years old (M=9.93; SD=1.05; 48% female). In total, 825 individuals participated. The aim was to observe each possible pair of participating individuals within each family interacting. This was achieved for all but 42 of the families. In total, 1,086 different dyads were observed.

Constructiveness was scored on an ordinal five-point scale, from 1 (*high destructive*) to 5 (*high constructive*). (Further details of the types of behaviour exhibited in each category can be found in the online supplementary material, Section S2 on page 2.) In practice, no observation was rated as fitting in the first category, so the response has four categories, with the lowest being 2 (*somewhat destructive*). Two trained raters double coded between 23% and 32% of tapes for all dyads to establish inter-rater reliability. Kappas ranged from κ=0.72 to 0.88 and percent agreement from 92% to 96%, depending on the dyad.

Each pair of participants was observed working on the task for a maximum of 5 min (i.e. 15 snapshots), with the average observation length across all dyads being 9.7 snapshots (i.e. 3 min 14 s). There are a few instances (123 out of 21,194 total observations, or 0.6%) where the constructiveness score was unavailable for a dyad. Since the software can only handle cases where the response at any given snapshot is observed for both members of an undirected dyad, or for neither, we set to missing a further 107 observations where the partner’s response at the same snapshot is missing, so that in total 230 observations are missing (1.0%). Further analysis of missing data patterns is given in the online supplementary material, Section S2 on pages 1–3.

Table 1 gives some details of the distribution of the response and the length of time over which dyads were observed, overall and by dyad type. (Full details can be found in online supplementary material, Table S1 on page 3.) The overall mean 3.51 lies close to the middle of the portion of the scale that is actually used, almost exactly midway between *not clearly destructive or constructive* and *somewhat constructive*. The means for the various dyad types also lie between these two points, but show some variation. The overall standard deviation 0.68 is a little over half a point on the scale. There is in general slightly less variability between dyads of the same type across different families (SD of mean) than within an individual dyad across time (mean SD), and the extent of each of these sources of variation is broadly similar across dyad types. Overall, dyads are observed on average for between nine and ten snapshots, i.e. between 3 min and 3 min 20 s.

**Table 1.** Descriptive statistics overall and by dyad type

	Mean	SD	Duration
Mother ▸ father	3.67	0.53	10.90
Father ▸ mother	3.55	0.54	10.90
Mother ▸ younger child	3.79	0.70	9.80
Younger child ▸ mother	3.11	0.67	9.80
Mother ▸ older child	3.78	0.66	10.55
Older child ▸ mother	3.31	0.68	10.55
Father ▸ younger child	3.89	0.63	9.73
Younger child ▸ father	3.30	0.61	9.73
Father ▸ older child	3.72	0.62	10.23
Older child ▸ father	3.32	0.55	10.23
Younger child ▸ older child	3.26	0.58	7.75
Older child ▸ younger child	3.37	0.63	7.75
Overall	3.51	0.68	9.76

## 6 Data analysis

### 6.1 Research questions

We have three research questions which form the focus of our analysis of these data. These can all be answered using the same model, that given by (4) and (5), that is, a dynamic SRM that treats the response as ordinal. Accordingly this is our model of interest and the only one for which results will be presented here. However, as we discuss in Section 6.2, we fit a number of other models to check that our model of interest is appropriate for these data and to provide a baseline indication of parameter estimates before dynamics are accounted for. We briefly summarise what these models tell us in Section 6.3.1 before turning to the model of interest for the remainder of Section 6. Full details of these additional models and their results are given in online supplementary material, Sections S3 on page 4, S5.1 on page 18, S5.2 on page 31, and S5.4 on page 63.

#### 6.1.1 How much variance in constructiveness is attributable to family, actor, partner, relationship, and snapshot when longitudinal data are used and dynamic effects are introduced into the SRM?

Previous cross-sectional studies of psychological measures in general find that the relationship is the most important source of variation and that actor and partner effects are small in comparison. We have longitudinal data and are thus able to separate residual error from the relationship component of variation. As discussed in Section 1, since in the model given by (10) and (5) we are comparing the effect of the relationship itself with the effects of the actor and partner (as the residual error is separately partitioned), we can estimate the relative importance of individual characteristics (here represented by the actor and partner) versus the importance of the combination of the two people in the dyad (relationship).

A model using the longitudinal data but which does not include dynamics finds moderate dyadic variances for each dyad type (see online supplementary material, Table S12 on page 32 for full details), and that the relationship makes a moderate to large contribution, depending on dyad type, to the time-invariant variance (i.e. the sum of the family, actor, partner and relationship effects) in this model (see online supplementary material, Table S14 on page 39). For three of the six undirected dyad types, there are moderate and significant dyadic reciprocities in this model, indicating an association between the time-invariant relationship effects in the two directed dyads. It is possible that the relationship variance can be substantially or completely explained by the effect of the partner’s response at the previous snapshot and/or the persistence of the actor’s own response from the previous snapshot once we add dynamics to the model, so we see whether the relationship variance still makes a similar contribution in the model with dynamics, and how the contributions of the other components of variation compare. (It is also possible that the dyadic reciprocities may be explained by the addition of dynamics; they are the focus of 6.1.3.)

#### 6.1.2 How much carry-over from one moment to the next is there in an individual’s behaviour (captured by the autoregressive effects $\phi_{1ij}$) and how much does each individual trigger the proximal behaviour of their partner (captured by the cross-lagged effects $\phi_{2ij}$)?

We expect autoregressive effects to vary by role and cross-lags by dyad type. The degree to which an individual can return to their own baseline is seen as an indicator of emotion regulation (Kuppens et al., 2010). Emotion regulation has been found to increase across development (Keltner et al., 2018) but no-one has examined this in the context of the SRM. As SRM studies find strong relationship effects (behaviour varies across relationships), an individual’s emotion regulation may vary across relationships. In the Hamaker et al. (2018) dynamic SEM, this component of the model is referred to as inertia; we have also referred to it in Section 4.1 as persistence. Significant autoregressive effects are hypothesised for both parents and children and we examine whether they differ as a function of age or vary across relationships.

As discussed in Section 1, we examine proximal triggers: the pathway of the partner’s behaviour at one snapshot influencing the actor’s at the next, picked up by the cross-lags $\phi_{2ij}$. We expect to see these proximal triggers more in family dyads with low power differentials (e.g. siblings and marital partners) than in those with high power differentials (parents and children) (Rasbash et al., 2011).

#### 6.1.3 Do siblings and adult partners show stronger dyadic reciprocity correlations on constructiveness than parents and children? Are the dyadic reciprocity correlations more important in explaining constructiveness than the cross-lagged influences across all dyads?

On the basis of the power differential findings outlined in RQ2, we expect the dyadic reciprocity correlations to be lower for parent–child dyads than the other dyads. Furthermore, we expect dyadic reciprocity correlations to be stronger than cross-lagged effects (proximal triggers). The cross-lagged parameter is more exacting, as it relies on getting the time frame of influence correct. For example, a similarity of behavioural response that occurs within a 20 s snapshot (one person’s lack of constructiveness is immediately followed by the other person’s lack of constructiveness) will not be counted as one person influencing the other but rather as the behaviour of the two people being correlated.

### 6.2 Modelling

Our model of interest is that given by (10) and (5), i.e. a dynamic SRM with the constructiveness response in a 20-s interval (snapshot) treated as ordinal with four categories; the autoregressive and cross-lag effects in (5) refer to the previous snapshot. As indicated in Section 4.1, we also fit a model with a linear time trend (allowed to differ across the six dyad types). We do not include time trends in our selected model since we are not interested in change in the means over time, and including the time trend adds complexity to the reparameterisation used to fit the models. However, we fit the model with time trends to check that their inclusion does not affect estimates of the parameters of interest. For the same reason, and to allow further comparisons outside the scope of this paper, we also fit the model given by (10), i.e. a non-dynamic ordinal SRM, and models given by (4) and (5) and by (4) alone, i.e. dynamic and non-dynamic SRMs which treat the response as continuous, but do not present the results of any of these in this article (although as mentioned in Section 6.1 we do briefly summarise the findings in Section 6.3.1). Full equations and results for all these models can be found in the online supplementary material (in Section S3.4 on page 8 and Table S37 on page 63 for the equations and results, respectively, of the model with time trends; in Table S8 on page 19 for the results of the non-dynamic ordinal SRM and dynamic and non-dynamic continuous SRMs; and in equation (S5) on page 8 and Table S12 on page 32 for the equations and results, respectively, of the ordinal SRM with autoregressive effects but no cross-lagged effects), as can other details of the modelling process omitted or only briefly touched on here (in online supplementary material, Section S3.5 on page 9 and Section S4 on page 11).

Models are fitted using MCMC, calling JAGS from within R. Results are presented for the parameterisation of (10) and (5), but the model fitted departs from this parameterisation in several respects, including that, as indicated in Section 4.3, we fit the autoregressive cross-lagged response form of the model.

The model of interest was run using 5 chains, each with a burn-in of 200,000 iterations following an adaptation phase of 1,000 iterations and monitored for 50,000 iterations (storing every 10th). The long burn-in was necessary due to poor mixing of the chains for the thresholds and intercepts. Starting values for each chain were randomly drawn. Stochastic convergence was judged both by examining trajectory plots and effective sample sizes and by checking that Gelman–Rubin potential scale reduction factors were close to 1 (Gelman et al., 2004). Estimation was good for all parameters, with trajectory plots showing no issues, effective sample sizes of at least 1,500, and potential scale reduction factors within 0.05 of 1.

VPCs give the proportion of the total unexplained variance attributable to each set of random effects. To address 6.1.1, we examine a similar decomposition, but of the time-invariant variance (i.e. the sum of the family, actor, partner, and relationship variances) rather than of the total variance. These and the differences between various parameters (such as the differences between the autoregressive effects for each possible pair of dyad types) are not parameters in the model but were calculated in R using the chains of parameter estimates. The shares of the time-invariant variance are calculated separately for each dyad type, using the family variance, the actor variance for the role who is the actor in that dyad type, the partner variance for the role who is the partner in that dyad type, and the relationship variance for that dyad type.

### 6.3 Results

Table 2 shows selected parameter estimates for our model of interest. (For estimates of parameters not shown here, see online supplementary material, Table S12 on page 32.)

**Table 2.** Selected parameter estimates (with 95% credible intervals) for ordinal dynamic SRM

Fixed part and innovation correlations			
	Mean	Percentiles	
		2.5th	97.5th
Autoregressive effects			
$\phi_{1}$ (M ▸ F)	0.068	−0.025	0.162
$\phi_{1}$ (F ▸ M)	0.202*	0.112	0.293
$\phi_{1}$ (M ▸ S_1_)	0.095*	0.029	0.161
$\phi_{1}$ (S_1_ ▸ M)	0.224*	0.150	0.297
$\phi_{1}$ (M ▸ S_2_)	0.102*	0.033	0.171
$\phi_{1}$ (S_2_ ▸ M)	0.237*	0.168	0.307
$\phi_{1}$ (F ▸ S_1_)	0.085	−0.006	0.175
$\phi_{1}$ (S_1_ ▸ F)	0.208*	0.121	0.296
$\phi_{1}$ (F ▸ S_2_)	0.108*	0.023	0.192
$\phi_{1}$ (S_2_ ▸ F)	0.120*	0.030	0.209
$\phi_{1}$ (S_1_ ▸ S_2_)	0.143*	0.051	0.233
$\phi_{1}$ (S_2_ ▸ S_1_)	0.164*	0.067	0.262
Cross-lags			
$\phi_{2}$ (M ▸ F)	−0.024	−0.113	0.066
$\phi_{2}$ (F ▸ M)	0.007	−0.078	0.092
$\phi_{2}$ (M ▸ S_1_)	−0.054	−0.135	0.027
$\phi_{2}$ (S_1_ ▸ M)	−0.009	−0.065	0.046
$\phi_{2}$ (M ▸ S_2_)	0.056	−0.014	0.127
$\phi_{2}$ (S_2_ ▸ M)	0.045	−0.015	0.106
$\phi_{2}$ (F ▸ S_1_)	0.016	−0.080	0.114
$\phi_{2}$ (S_1_ ▸ F)	0.001	−0.073	0.075
$\phi_{2}$ (F ▸ S_2_)	−0.016	−0.115	0.080
$\phi_{2}$ (S_2_ ▸ F)	0.002	−0.068	0.073
$\phi_{2}$ (S_1_ ▸ S_2_)	0.099*	0.015	0.180
$\phi_{2}$ (S_2_ ▸ S_1_)	0.028	−0.067	0.123
Innovation correlations			
$\rho_{\eta }$ (M&F)	−0.021	−0.133	0.093
$\rho_{\eta }$ (M&S_1_)	0.089*	0.029	0.149
$\rho_{\eta }$ (M&S_2_)	0.104*	0.042	0.165
$\rho_{\eta }$ (F& S_1_)	0.184*	0.104	0.263
$\rho_{\eta }$ (F& S_2_)	0.108*	0.019	0.195
$\rho_{\eta }$ (S_1_& S_2_)	0.326*	0.241	0.407

Random part (other than innovation correlations)			
	Mean	Percentiles	
		2.5th	97.5th
Family variance			
$\sigma_{f}^{2}$	0.031	0.001	0.111
Actor variances			
$\sigma_{a}^{2}$ (M)	0.200	0.121	0.294
$\sigma_{a}^{2}$ (F)	0.183	0.110	0.273
$\sigma_{a}^{2}$ (S_1_)	0.121	0.072	0.182
$\sigma_{a}^{2}$ (S_2_)	0.101	0.059	0.157
Partner variances			
$\sigma_{p}^{2}$ (M)	0.084	0.049	0.132
$\sigma_{p}^{2}$ (F)	0.061	0.037	0.094
$\sigma_{p}^{2}$ (S_1_)	0.115	0.066	0.180
$\sigma_{p}^{2}$ (S_2_)	0.087	0.050	0.136
Relationship variances			
$\sigma_{d}^{2}$ (M ▸ F)	0.157	0.081	0.266
$\sigma_{d}^{2}$ (F ▸ M)	0.122	0.063	0.210
$\sigma_{d}^{2}$ (M ▸ S_1_)	0.451	0.300	0.630
$\sigma_{d}^{2}$ (S_1_ ▸ M)	0.350	0.223	0.504
$\sigma_{d}^{2}$ (M ▸ S_2_)	0.386	0.243	0.557
$\sigma_{d}^{2}$ (S_2_ ▸ M)	0.315	0.193	0.463
$\sigma_{d}^{2}$ (F ▸ S_1_)	0.296	0.161	0.472
$\sigma_{d}^{2}$ (S_1_ ▸ F)	0.119	0.063	0.204
$\sigma_{d}^{2}$ (F ▸ S_2_)	0.218	0.110	0.362
$\sigma_{d}^{2}$ (S_2_ ▸ F)	0.115	0.062	0.191
$\sigma_{d}^{2}$ (S_1_ ▸ S_2_)	0.096	0.052	0.161
$\sigma_{d}^{2}$ (S_2_ ▸ S_1_)	0.201	0.106	0.327
Generalised reciprocity correlations			
$\rho_{ap}$ (M)	0.171	−0.188	0.485
$\rho_{ap}$ (F)	0.041	−0.301	0.374
$\rho_{ap}$ (S_1_)	0.302	−0.039	0.580
$\rho_{ap}$ (S_2_)	0.360*	0.025	0.622
Dyadic reciprocity correlations			
$\rho_{d}$ (M&F)	0.173	−0.254	0.550
$\rho_{d}$ (M&S_1_)	0.503*	0.257	0.709
$\rho_{d}$ (M&S_2_)	0.281	−0.009	0.534
$\rho_{d}$ (F& S_1_)	0.050	−0.364	0.443
$\rho_{d}$ (F& S_2_)	0.151	−0.268	0.515
$\rho_{d}$ (S_1_& S_2_)	0.321	−0.071	0.633

*Note*. SRM = social relations model.

*Significant at the 5% level.

#### 6.3.1 Preliminary analysis

The results are broadly similar whether we treat the response as continuous or ordinal. As expected given that the model estimates polychoric rather than Pearson correlations, as discussed in Section 4.2, the point estimates of the autoregressive effects (the $\phi_{1}$), cross-lags (the $\phi_{2}$), generalised reciprocity correlations (i.e. actor–partner correlations, $\rho_{ap}$), dyadic reciprocity correlations (i.e. relationship correlations, $\rho_{d}$), and innovation correlations ($\rho_{\eta }$) are mostly larger when treating the response as ordinal rather than continuous. Although when including linear time trends we find that there is a significant (negative) trend for five of the six dyad types, these trends are small (the largest in magnitude being −0.06, so that it would take around 30 snapshots for predicted constructiveness to decrease by 1 category) and there is little difference in the parameter estimates between this model and our model of interest, justifying our omission of time trends in that model.

We now turn to the model of interest for the remainder of this section to address our research questions.

#### 6.3.2 Research question 1

As with the other parameters common to both models, the estimates for the relationship variances are not much different in the model of interest compared to a model without dynamics (see online supplementary material, Table S12 on page 32). Thus neither the actor’s own response or their partner’s response at the previous snapshot seem to explain much of the relationship variance.

Table 3 shows, for each dyad type, the proportion of the time-invariant variance (i.e. the sum of the family, actor, partner, and relationship variances) accounted for by each component of variance. (These are similar to VPCs, but it is not the total variance that we are partitioning, since we are excluding both temporal fluctuation and measurement error.) Even though we have removed both temporal fluctuation and measurement error to another component of variance, and added dynamics, the relationship effects still make a moderate to large contribution, accounting for a little over a quarter of the variance to a little under two thirds. They are perhaps generally more important than actor effects (which account for just under a fifth to somewhat under half), or partner effects (a little over a tenth to just over a quarter), but the credible intervals are wide for most dyad types; and indeed the point estimate of the actor effect is actually larger than that of the relationship effect in four dyad types (though the point estimate of the partner effect is smaller than that of the relationship effect in every case). The family effect is relatively unimportant, with point estimates of under 10% in all dyad types and under 5% in three types, though again the credible intervals are wide, with the upper limits reaching somewhere between a quarter and a third for some dyad types, but the lower limits extending almost all the way to no contribution for all types.

**Table 3.** Proportions of time-invariant variance for ordinal dynamic SRM (with 95% credible intervals)

	Family	Actor	Partner	Relationship
M ▸ F	0.066 (0.001,0.219)	0.448 (0.288,0.609)	0.138 (0.080,0.216)	0.349 (0.204,0.511)
F ▸ M	0.071 (0.001,0.239)	0.437 (0.278,0.592)	0.202 (0.115,0.314)	0.290 (0.164,0.442)
M ▸ ${\text{S}}_{1}$	0.038 (0.001,0.132)	0.252 (0.155,0.364)	0.145 (0.081,0.229)	0.564 (0.426,0.691)
${\text{S}}_{1}$ ▸ M	0.052 (0.001,0.175)	0.209 (0.120,0.320)	0.145 (0.081,0.231)	0.595 (0.449,0.724)
M ▸ ${\text{S}}_{2}$	0.043 (0.001,0.148)	0.286 (0.172,0.418)	0.124 (0.070,0.199)	0.546 (0.391,0.688)
${\text{S}}_{2}$ ▸ M	0.057 (0.001,0.193)	0.193 (0.107,0.308)	0.160 (0.089,0.258)	0.590 (0.428,0.730)
F ▸ ${\text{S}}_{1}$	0.049 (0.001,0.170)	0.296 (0.175,0.433)	0.186 (0.101,0.297)	0.469 (0.304,0.626)
${\text{S}}_{1}$ ▸ F	0.088 (0.002,0.284)	0.368 (0.217,0.533)	0.186 (0.109,0.287)	0.358 (0.207,0.532)
F ▸ ${\text{S}}_{2}$	0.058 (0.001,0.198)	0.356 (0.209,0.516)	0.169 (0.094,0.269)	0.416 (0.244,0.588)
${\text{S}}_{2}$ ▸ F	0.095 (0.002,0.302)	0.332 (0.192,0.492)	0.201 (0.118,0.310)	0.372 (0.223,0.537)
${\text{S}}_{1}$ ▸ ${\text{S}}_{2}$	0.088 (0.002,0.287)	0.363 (0.222,0.514)	0.261 (0.152,0.391)	0.287 (0.165,0.436)
${\text{S}}_{2}$ ▸ ${\text{S}}_{1}$	0.067 (0.001,0.226)	0.229 (0.128,0.355)	0.259 (0.146,0.395)	0.446 (0.278,0.610)

*Note*. SRM = social relations model.

With the addition of dynamics, two of the dyadic reciprocities which were significant in the model without dynamics have become non-significant (with the three which were already non-significant in that model remaining non-significant here). The third remains significant and moderately strong.

#### 6.3.3 Research question 2

The autoregressive effects (the $\phi_{1}$ parameters) are all positive and, with two exceptions, significant at the 5% level. Thus we can conclude that there is persistence from one snapshot to the next in individuals’ behaviour. However, this is not strong, with the largest point estimate being 0.237. (Recall that we noted in Section 4.2 that these can be interpreted as correlations between an individual’s constructiveness at the current snapshot and their constructiveness at the previous snapshot.) They do generally seem to be higher for children than for parents, with the notable exception of fathers acting towards mothers.

**Figure 1.**
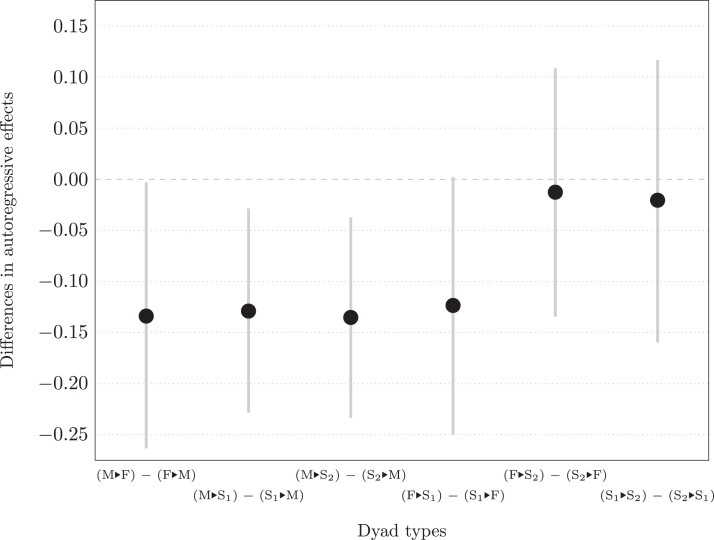
Point estimates and 95% credible intervals for differences between estimated autoregressive effects for each undirected dyad type calculated from parameter chains.

Figure 1, which shows the differences between the estimated autoregressive effects for the directed dyads associated with each undirected dyad, allows us to examine whether the autoregressive effects for one family role acting towards another are larger than the autoregressive effects for the same combination of family roles with actor and partner reversed. As described in Section 6.2, we use the chains of parameter estimates to calculate the difference between the autoregressive effects at each stored iteration, to produce a chain of differences. The mean of this chain of estimates provides the point estimate for the differences, shown in Figure 1 as black dots, while the 2.5th and 97.5th percentiles provide the lower and upper limits, respectively, of 95% credible intervals, shown as light grey lines.

For fathers, when they are interacting with mothers their autoregressive effects are (on average) higher than those of mothers, but there is no evidence of their having a higher autoregressive effect than their child when interacting with either child. Similarly, both older and younger children have higher autoregressive effects than mothers when interacting with them, but we cannot conclude that either have a higher autoregressive effect than fathers when interacting with them (though for younger siblings the point estimate of the difference is in the same direction and of a similar magnitude to the difference for younger siblings interacting with mothers, and only just misses being significant), nor that either has a higher autoregressive effect than the other when they interact together. In contrast, mothers have low autoregressive effects regardless of partner. In summary, although we see a bit of support for the hypothesis that emotion regulation increases with age, we also see that it may depend on the interactional partner.

With respect to the cross-lags (the $\phi_{2}$ parameters), these are mostly not significant at the 5% level. The exception is the cross-lag for younger siblings acting towards older siblings, which has a point estimate of 0.099. This is not a very strong effect: a difference of 1 unit either way in the older sibling’s unobserved underlying constructiveness at the previous snapshot leads to a difference of a 10th of a unit in the younger sibling’s predicted underlying constructiveness. (For context, one unit is roughly half the distance between either pair of consecutive thresholds and exactly the standard deviation of the innovations.)

#### 6.3.4 Research question 3

One of the dyadic reciprocity correlations (i.e. correlation between the relationship effects for dyads consisting of the same individuals, the $\rho_{d}$ parameters) is significant and quite strong (having a point estimate of 0.503), although none of the other dyadic reciprocity correlations are significant. Contrary to our hypothesis, this involves a parent and a child. There is no evidence that the dyadic reciprocity for any particular undirected dyad type is higher than the dyadic reciprocity for any other undirected dyad type so we cannot conclude that the dyadic reciprocities are stronger between siblings and/or between adult partners than they are between parents and children.

Returning to the cross-lag parameters (the $\phi_{2}$), we find one of them to be significant (the one with the largest point estimate). In line with our hypothesis, this is for a sibling-sibling dyad. We can conclude that it is larger than the cross-lags for three of the other dyad types: mothers acting towards fathers (where the difference is only just significant), mothers acting towards younger children, and younger children acting towards mothers. (See online supplementary material, Table S23 on page 49 for full details.) We thus have limited evidence in support of the hypothesis that the cross-lagged effects are stronger for siblings acting towards each other than for parents acting towards children or children acting towards parents, but no evidence to support their also being stronger for adult partners acting towards each other.

While the point estimates of some of the dyadic reciprocities indicate moderate or fairly strong correlations (with others being weak), they all have wide credible intervals, meaning that while it is plausible they are moderate to strong, for most dyad types it is also plausible they are weak to non-existent or even that the actor’s average constructiveness towards a particular partner has an inverse relationship to that partner’s average constructiveness towards them. The exception is that for mothers and younger children, which is found to be significant, with plausible values ranging from moderate and positive to strong and positive. This dyadic reciprocity is larger (both in the sense of being more positive and in the sense of having a greater absolute value) than every cross-lag parameter (that is, for each cross-lag parameter, when we subtract it from this dyadic reciprocity, the credible interval lies entirely above zero). No dyadic reciprocities are found to be smaller (whether less positive or having a lower absolute value) than any cross-lag. There is thus limited evidence to suggest that, in line with our hypothesis, individuals respond in kind more to their partner’s general behaviour towards them than to their behaviour at any specific moment.

However, it is possible that individuals respond either much more quickly, or slowly, than our 20 s snapshots, in which case this response would not be picked up by the cross-lag. A quicker response would be picked up by the innovation correlations (i.e. the $\rho_{\eta }$ parameters). All but one of these are significant, though they are weak to moderate. The dyadic reciprocity for mothers and younger children is stronger than the innovation correlation (at both the first and subsequent snapshots), but we cannot say which is stronger for the other dyad types. The innovation correlation for snapshots after the first for the relevant undirected dyad type is stronger than the cross-lag parameter for mothers acting towards older children, older children acting towards mothers, fathers acting towards younger siblings, younger siblings acting towards fathers, and older siblings acting towards fathers; we cannot say which is stronger for the other dyad types. (See online supplementary material, Tables S27 on page 55 to S36 on page 62 for full details of the differences between the estimates of the dyadic reciprocities, innovation correlations, and cross-lag parameters.)

Since the cross-lag parameters are almost all non-significant while the autoregressive parameters are mostly significant, it is natural to consider whether a model which includes autoregressive but not cross-lagged effects would be more appropriate. This is beyond the scope of this article, but we present the results in online supplementary material, Table S12 on page 32; the estimates for parameters common to this model and the model of interest are similar in both models, so similar conclusions would be reached by fitting either.

#### 6.3.5 Other parameters of interest

The generalised reciprocity correlation (i.e. the correlation between the actor and partner effects for the same individual, the $\rho_{ap}$ parameters) is significant and fairly strong (point estimate 0.360) for the older child, but there is no evidence of any generalised reciprocity correlation for any other family role.

## 7 Discussion

In this paper, we considered the problem of studying the dynamics of individual behaviour where pairs of subjects (dyads) are observed to interact with each other over time, and individuals are clustered and distinguished by their roles within the cluster. We propose an extension of the widely used SRM that is suitable for round-robin designs, which can be framed as a cross-classified multilevel (random effects) model where the responses have a complex correlation structure within clusters. In addition to the actor and partner effects that can be identified in a cross-sectional SRM, we specify first-order autoregressive and cross-lagged effects to study the dynamics of individual behaviour. Autoregressive (or lagged) effects allow for persistence in an individual’s response, while cross-lagged effects allow for the influence of a person’s response at time t−1 on their dyadic partner’s response at *t*. Moreover, longitudinal data allow separation of relationship (dyad) effects from measurement error, which are confounded in a cross-sectional design. We show that the dynamic SRM, with an autoregressive and cross-lagged structure imposed on the residuals, is equivalent to a SRM extension of the vector autoregressive model from econometrics and psychometrics where the dynamic structure is specified among the responses. Our approach can handle continuous, binary and ordinal responses, where for discrete responses the model is expressed in terms of underlying continuous latent response variables. Model estimation can be carried out using MCMC methods implemented in Bayesian software.

The development of the dynamic SRM was motivated by a study of intra-family dynamics using repeated observations on the behaviour of pairs of family members as they discuss a recent conflict. One question of interest is how much of the variation in the degree of constructiveness directed from one family member to another can be attributed to family, actor, partner, and relationship effects and to the residual. We find most of the variation is attributable to the residual (i.e. the time-varying behaviour that may be meaningful, combined with measurement error), and the remainder is mostly attributable to the actor, partner, and relationship effects in roughly equal measure (though perhaps the actor effects account for a little more than partner effects, and relationship effects for a little more than actor effects); little variation is attributable to family effects. We also investigate the persistence in individuals’ behaviour and the proximal triggers of one person’s behaviour on that of their interactional partner in the task, and the extent to which these vary according to the particular combination of actor and partner family roles in the dyad. We find that for most combinations there is moderate persistence, but no evidence of any influence of one individual’s behaviour on another’s. The degree of persistence shown varies between combinations of family role, with some evidence of less persistence for mothers compared to children as actors but not for fathers; the degree of persistence seems to be explained more by the combination of family roles than simply the actor’s or partner’s family role. Finally, we look at the extent to which one individual’s behaviour towards another in general across time is related to the second individual’s behaviour towards the first, after accounting for both individuals’ actor and partner effects, their persistence, and the moment-to-moment influence of each on the other. We find little evidence of any such generalised reciprocity. We are mostly not able to say whether the strongest effect a partner has on an actor is through their behaviour at that moment, their behaviour immediately before, or their average behaviour over time.

The dynamic SRM has many other potential applications in the social sciences where grouped or ungrouped dyadic data are collected using round-robin and related designs, and responses are often discrete. Another family example is a study of inter-generational exchanges of support between family members across the lifecourse, where there may be interest in the extent of family, giver, receiver, and relationship effects on exchanges, the persistence of exchanges between individuals A and B over time, and whether A giving support to B at t−1 influences whether B provides (possibly a different form of) support to A at *t*. In management research, the model could be used to study the dynamics of interactions between coworkers within an organisation and variation in persistence and influence according to the relative seniority of individuals in a dyad.

Our model assumes that the dynamics of interactions follow a first-order Markov process, although in principle second and higher order lags and cross-lags may be included. While the trajectories in behaviour over time were not a focus of our analysis, it is possible to combine the dynamic SRM with the growth curve SRM (Nestler et al., 2017) in an SRM extension of the autoregressive latent trait (ALT) model proposed by Bollen and Curran (2004). Such a model would allow the slopes of dyad-specific time trends (assumed fixed in our analysis), to vary randomly across partners, actors, and relationships. However, an ALT SRM would have a highly complex implied covariance structure and should be applied with caution; even in the case of a univariate response ALT model, the results may be misleading if there is any misspecification of the autoregressive or growth component of the model (Voelke, 2008). Another potentially useful generalisation would be to allow the random effect and residual variances to depend on characteristics other than dyad type; for example, the relationship variance for sibling pairs may depend on their age difference and sex composition.

## Supplementary Material

qnad115_Supplementary_Data

## Data Availability

The analysis is based on data from the *Kids, Families and Places* study. At the time of publication, the data for this paper are not yet publicly available because of ongoing analyses and research ethics board procedures for data access. For data access please write to responsive_interactions@utoronto.ca. Further details about the study can be found at https://www.oise.utoronto.ca/kfp/Researchers/index.html.
