# Supplementary material for: A dynamic social relations model for clustered longitudinal dyadic data with continuous or ordinal responses
Source: J R Stat Soc Ser A Stat Soc. 2023 Sep 5;187(2):338–57. doi: 10.1093/jrsssa/qnad115 (PMC11090398; doi:10.1093/jrsssa/qnad115)
Supplement: qnad115_Supplementary_Data [file qnad115_supplementary_data.pdf]

A dynamic social relations model for clustered longitudinal dyadic data: Supplementary materials

Rebecca Pillinger, Fiona Steele, George Leckie, and Jennifer Jenkins

1st July 2023

London School of Economics

A dynamic social relations model for clustered longitudinal dyadic data: Supplementary materials

Rebecca Pillinger, Fiona Steele, George Leckie, and Jennifer Jenkins

1st July 2023

S1 Introduction

This document provides supplementary materials for the paper "A dynamic social relations model for clustered longitudinal dyadic data" ((Pillinger et al., Submitted)). Sections S2 and S4 provide fuller details on respectively the data we analyse and the estimation process than there was space for in the article. Section S3 gives the equations of all models for which results are shown in this document, and explains how the additional quantities we present (such as VPCs) were calculated. Section S5 provides results for some alternative models that were not selected as our model of interest and for which results accordingly do not appear in the article: models with and without dynamics treating the response as continuous; an ordinal response model without dynamics; and an ordinal response model with dynamics and with fixed linear time trends. It also provides some additional quantities for the model of interest: correlations between the response at successive snapshots; the total correlation between each pair of dyad types; differences between various model parameters; and the proportions of the total dyad-type variance (i.e. the total variance excluding the innovation variance) due to each component of variation.

S2 Data

Participants in this study were originally recruited as part of the *Kids, Families and Places* study, a birth-cohort longitudinal study that followed children born between February 2006 and February 2008 in Toronto or Hamilton, Canada, into school (Meunier et al., 2013; Browne et al., 2018). This sample was recontacted to take part in a subsequent study, the goals of which were to understand the development of cooperation in family relationships. The data for the current study were drawn from the cooperation study. Participants from 223 families were video recorded working on a conflict discussion task in pairs, and raters subsequently watched the recordings and scored each individual's constructiveness at 20 second intervals ("snapshots"). In most families, the participants were the mother (M), father (F), younger child (S_1), and older child (S_2), but in 65 families the father did not participate (in some cases because it was a single-parent family and in others because the father did not wish to participate), and in two families one of the children did not participate. The children who were newborns at the time of initial recruitment–termed the "younger siblings" or "younger children" in this study–were between five and nine years old when these data were collected (M=7.34; SD=0.82; 51% female). Their next oldest siblings ("older siblings" or "older children") were seven to thirteen years old (M=9.93; SD=1.05; 48% female). In total, 825 individuals participated.

The aim was to observe each possible pair of individuals within each family interacting. This was achieved for 123 of the families with mother, father, younger child, and older child participants, 56 of the families with only mother, younger child and older child participants, and both the family with mother, father, and younger child participants and the family with mother, father, and older child participants. For the remaining families, some of the possible

pairs were not observed, with the most common patterns in families with two parents participating being for just the parents not to be observed interacting together (12 families) or just the siblings not to be observed interacting together (8 families), and the most common patterns in families without a participating father being for just the mother and younger child not to be observed interacting together (4 families) or just the siblings not to be observed interacting together (also 4 families). In total, 1086 different undirected dyads and 2172 different directed dyads were observed.

Constructiveness was scored on an ordinal five-point scale:

1	*High destructive*	Significant belittling, anger, coercion, power assertion or ignoring (i.e. marked disengagement in face of partner's efforts to engage). High costs to the partner (all negative categories checked and no positive categories)
2	*Somewhat destructive*	Low level of belittling, anger, coercion, power assertion or ignoring/blocking (i.e. marked disengagement in face of partner's efforts to engage). Some costs to the partner (more negative than positive categories checked)
3	*Not clearly destructive or constructive*	Involved destructive as well as constructive elements (equal number of positive and negative categories checked)
4	*Somewhat constructive*	Some encouragement, positive affect, interest, cooperation. Involves little cost to partner (more positive than negative categories checked)
5	*High constructive*	A lot of encouragement, positive affect, interest, de-escalation. Involves no costs to the partner (all positive categories checked and no negative categories)

In practice, no observation was rated as fitting in the first category, *high destructive*, so the response has four categories.

Each pair of participants was observed working on the task for a maximum of 5 minutes (i.e. 15 snapshots), with the average observation length across all dyads being 9.7 snapshots (i.e. 3 minutes 14 seconds). There are a few instances (123 out of 21194 total observations, or 0.6%) where a constructiveness score is not assigned to a particular individual when interacting with a particular partner at a particular snapshot. This was because there were occasional times when the raters were not able to see or hear the participants clearly enough in the recording to assign a score, for example because they had turned away from the camera or were reaching for something out of the frame. Since the software can only handle cases where the response at any given snapshot is observed for both members of a dyad, or for neither, we set to missing a further 107 observations where the partner's response at the same snapshot is missing, so that in total 230 observations are missing (1.0%). Missingness seems to be related to the value of the individual's own response at the previous snapshot (when this is observed), both before and after we set the further 107 observations to missing, with 0.6% of observations following a response of 3 missing but only 0.4% of observations following a response of 2, 0.3% of observations following a response of 4, and 0.0% of observations following a response of 5 before we set the additional observations to missing, and 1.0% of observations following a response of 3 missing but only 0.9% of observations following a response of 2, 0.6% of observations following a response of 4, and only 0.1% of observations following a response of 5 after we set the additional observations to missing. It also seems to be related to the value of the partner's response at the previous snapshot (again, when this is observed) - moderately so before we set the additional observations to missing (with 0.6% of observations following a response of 3 missing, but only 0.5% following a response of 2 or a response of 4, and 0.4% following a response of 5), and more strongly so (presumably because the partner's response at the previous snapshot is related to the missingness of the partner's own response at the current snapshot, which now also implies missingness of the individual's own response) after setting the additional responses to missing (with 1.0% missing following a partner's response of 3, but only 0.9% following a partner's response of 2, 0.6% following a partner's response of 4, and 0.1% following a partner's response of 5). These relationships between the value of the individual's own or their partner's

Table S1: Descriptive statistics overall and by dyad type

	Mean	Med.	S.D. of mean	Mean S.D.	Min.	Max.	Range	Duration	Obs.
Mother ▸ Father	3.665	4	0.301	0.450	3.007	4.113	1.106	10.901	10.866
Father ▸ Mother	3.549	4	0.302	0.429	3.007	4.021	1.014	10.901	10.866
Mother ▸ Younger child	3.784	4	0.427	0.559	2.963	4.461	1.498	9.797	9.783
Younger child ▸ Mother	3.137	3	0.453	0.482	2.590	3.788	1.198	9.797	9.783
Mother ▸ Older child	3.791	4	0.407	0.492	3.032	4.418	1.386	10.545	10.505
Older child ▸ Mother	3.313	3	0.434	0.506	2.682	3.923	1.241	10.545	10.505
Father ▸ Younger child	3.856	4	0.399	0.479	3.127	4.453	1.327	9.727	9.720
Younger child ▸ Father	3.296	3	0.365	0.465	2.753	3.880	1.127	9.727	9.720
Father ▸ Older child	3.699	4	0.375	0.513	2.941	4.296	1.355	10.230	10.013
Older child ▸ Father	3.334	3	0.306	0.457	2.842	3.914	1.072	10.230	10.013
Younger child ▸ Older child	3.286	3	0.342	0.445	2.849	3.820	0.971	7.751	7.439
Older child ▸ Younger child	3.400	3	0.387	0.500	2.859	3.922	1.063	7.751	7.439
Overall	3.506	4	0.676		2.000	5.000	3.000	9.758	9.652

response at one snapshot and the missingness of the individual's response at the next provide a further motivation for including dynamics in the model we fit, since otherwise its imputation for missing responses would be biased.

Table S1 gives some details of the distribution of the response and number of observations overall and for each dyad type. In the Overall row, all values are calculated by simply pooling all observations, except the duration and number of (non-missing) observations which are calculated for each dyad and then averaged across all dyads. The value straddling the S.D. of Mean and Mean S.D. columns is simply the standard deviation of all observations pooled. In the other rows, the mean, median, standard deviation, minimum, maximum, and range are calculated across all snapshots for each dyad of that type, and the number of observations (i.e. the duration for which the dyad was observed) and of non-missing observations are counted. The means across all dyads of each type of the means, s.d.s, minima, maxima, ranges, numbers of observations, and of non-missing observations are respectively the values in the Mean, Mean S.D., Min., Max., Range, Duration, and Obs. columns. The medians across all dyads of each type of the medians are the values in the Med. column. The standard deviations across all dyads of each type of the means are the values in the S.D. of Mean column. Note that this is different to the way the values shown in Table 1 of the article are calculated, since those are the mean and standard deviations of all observations from dyads of a particular dyad type pooled, rather than calculating for each individual dyad of that type separately and then averaging across dyads as we do here. The Mean S.D. and S.D. of Mean columns give a rough idea of how the magnitude of the variance within dyads over time compares to the magnitude of the variance across dyads of the time-invariant factors that contribute to the response. The models we fit will of course give a much better picture of this.

The overall mean lies close to the middle of the portion of the scale that is actually used, almost exactly midway between *Not clearly destructive or constructive* and *Somewhat constructive*. The means for the various dyad types also lie between these two points, but show some variation, with the least constructive dyad type being younger children acting towards mothers, with a mean of 3.1, and the most constructive fathers acting towards younger children, with a mean of 3.9. Overall, the median is 4, *Somewhat constructive*, but only half the dyad types (those with a parent as the actor) share this median, with the other half (those with a child as the actor) having a median of 3, *Not clearly destructive or constructive*. Dyad types also differ somewhat in the average lowest value exhibited (with the lowest being younger children acting towards mothers, at 2.6, and the highest fathers acting towards younger children, at 3.1) and the average highest value exhibited (with again the lowest being younger children acting towards mothers, at 3.8, and the highest being mothers acting towards younger children, at 4.6).

The standard deviation overall is 0.7, or a little over half a point on the scale. There is in general slightly less variability between dyads of the same type across different families than within an individual dyad across time, and the extent of each of these sources of variation is broadly similar across dyad types, with mothers acting towards fathers being

the most similar across families (with the means across time for this dyad type having a standard deviation of 0.30) and younger children acting towards mothers being the least similar across families (with the means across time for this dyad type having a standard deviation of 0.45); and fathers acting towards mothers being the most consistent across time (with a standard deviation of 0.43 on average) and mothers acting towards younger children being the least consistent across time (with a standard deviation of 0.56 on average). For most dyad types, individual dyads exhibit on average somewhere between 2 and 3 different values on the scale (generally closer to 2 than 3), indicating a certain degree of consistency across time, with younger children acting towards older children the most consistent (with an average range of 0.97) and mothers acting towards younger children the least consistent (with an average range of 1.50).

Since there are few missing values, the average numbers of observations and of non-missing observations are similar, both overall and for each dyad type. Overall, dyads are observed on average for between 9 and 10 snapshots, i.e. between 3 minutes and 3 minutes 20 seconds, and also have between 9 and 10 non-missing observations. Since observations come from pairs of people observed working together, dyad types consisting of the same individuals (e.g. mothers acting towards fathers and fathers acting towards mothers) have the same number of observations; and since we set an individual's response to be missing at any snapshot where their partner's response was missing, they also have the same number of non-missing observations. Mothers and fathers are observed for longest on average, at nearly 11 snapshots (3 minutes 40 seconds), and older and younger children for the shortest time, at between 7 and 8 snapshots (2 minutes 20 seconds to 2 minutes 40 seconds).

S3 Models

S3.1 Longitudinal social relations model

The simplest model which we present results for in this document is a longitudinal social relations model with no dynamics, in which the response is treated as continuous. This model is given by:

$$\begin{aligned}
y_{tijk} &= \mu_{ij} + \theta_{ijk} + \eta_{tijk} \\
\theta_{ijk} &= f_k + a_{ik} + p_{jk} + d_{ijk}, \quad i \neq j \\
f_k &\sim \mathrm{N}(0, \sigma_f^2) \\
\begin{bmatrix} a_{ik} \\ p_{ik} \end{bmatrix} &\sim \mathrm{N}\left(\begin{bmatrix} 0 \\ 0 \end{bmatrix}, \begin{bmatrix} \sigma_{ai}^2 & \\ \rho_{api}\sigma_{ai}\sigma_{pi} & \sigma_{pi}^2 \end{bmatrix} \right) \\
\begin{bmatrix} d_{ijk} \\ d_{jik} \end{bmatrix} &\sim \mathrm{N}\left(\begin{bmatrix} 0 \\ 0 \end{bmatrix}, \begin{bmatrix} \sigma_{dij}^2 & \\ \rho_{dij}\sigma_{dij}\sigma d ji & \sigma_{dji}^2 \end{bmatrix} \right) \\
\begin{bmatrix} \eta_{tijk} \\ \eta_{tjik} \end{bmatrix} &\sim \mathrm{N}\left(\begin{bmatrix} 0 \\ 0 \end{bmatrix}, \begin{bmatrix} \sigma_{\eta ij}^2 & \\ \rho_{\eta ij}\sigma_{\eta ij}\sigma \eta ji & \sigma_{\eta ji}^2 \end{bmatrix} \right)
\end{aligned} \tag{S1}$$

where i indexes actors, j indexes partners, k indexes families, t indexes snapshots, and y_{tijk} is the degree of constructiveness shown at snapshot t by actor i in family k towards partner j in the same family. The actors and partners are indexed by family role, with 1 denoting the mother, 2 the father, 3 the younger child, and 4 the older child, rather than by a unique index for each individual in the dataset or even for each individual when acting towards each other in their family, since combining this index with the family index still allows each individual to be uniquely identified, and since in this and future models we will want to include parameters which are specific to a family role or combination of family roles rather than to an individual.

One such parameter is the intercepts μ_{ij}, of which there is one for each dyad type, capturing the average constructiveness across families and across time shown by individuals in role i towards individuals in role j. These intercepts

are the only fixed-part parameters.

In the random part, we have θ_{ijk}, the deviation (from the average constructiveness μ_{ij} shown, across time, across all families by individuals in role i towards individuals in role j) in the average constructiveness shown across time by the individual in role i towards the individual in role j in family k. (Note that this is a difference in notation from that used in the paper, where θ_{ijk} includes the intercepts μ_{ij} and thus does not represent the deviation in an individual's average constructiveness from the average across families. This is because in Section S4.3 and elsewhere we will find it useful to have a parameter collecting the random effects other than the innovations, but will still want to write out the intercepts and time trends.) This is decomposed into family effects f_k, actor effects a_{ik}, partner effects p_{jk}, and dyad-type effects d_{ijk}, with associated variances σ^2_f, σ^2_{ai}, σ^2_{pj}, and σ^2_{dij} respectively. There is just one family variance, but there is a separate actor variance and a separate partner variance for each family role, and a separate dyad-type variance for each dyad type. Actor and partner effects for the same family role are allowed to be correlated, with covariances σ_{api} (known as the generalised reciprocities), and dyad-type effects for dyad types involving the same two family roles (i.e. involving the same undirected dyad) also allowed to be correlated, with covariance σ_{dij} (known as the dyadic reciprocities).

At the lowest level, we have the innovations e_{tijk}, which are the deviation at snapshot t in the degree of constructiveness shown by the individual in role i towards the individual in role j in family k from the average across time for that pair of individuals, with associated variances σ^2_{eij}. There is a separate innovation variance for each dyad type. Innovations at the same snapshot for dyad types involving the same two family roles are allowed to be correlated, with correlations ρ_{eij}.

S3.2 Dynamic social relations model

The other continuous response model we fit is a social relations model that incorporates dynamics – i.e. a continuous version of our model of interest. This is given by

$$
\begin{aligned}
y_{tijk} &= \mu_{ij} + \theta_{ijk} + e_{tijk} \\
e_{tijk} &= \phi_{1ij} e_{(t-1)ijk} + \phi_{2ij} e_{(t-1)jik} + \eta_{tijk}, \quad \text{for } t > 1 \\
\theta_{ijk} &= f_k + a_{ik} + p_{jk} + d_{ijk}, \quad \text{for } i \neq j \\
f_k &\sim \mathrm{N}(0, \sigma^2_f) \\
\begin{bmatrix} a_{ik} \\ p_{ik} \end{bmatrix} &\sim \mathrm{N}\left(\begin{bmatrix} 0 \\ 0 \end{bmatrix}, \begin{bmatrix} \sigma^2_{ai} & \\ \rho_{api}\sigma_{ai}\sigma_{pi} & \sigma^2_{pi} \end{bmatrix} \right) \\
\begin{bmatrix} d_{ijk} \\ d_{jik} \end{bmatrix} &\sim \mathrm{N}\left(\begin{bmatrix} 0 \\ 0 \end{bmatrix}, \begin{bmatrix} \sigma^2_{dij} & \\ \rho_{dij}\sigma_{dij}\sigma_{dji} & \sigma^2_{dji} \end{bmatrix} \right) \\
\begin{bmatrix} e_{1ijk} \\ e_{1jik} \end{bmatrix} &\sim \mathrm{N}\left(\begin{bmatrix} 0 \\ 0 \end{bmatrix}, \begin{bmatrix} \sigma^2_{e1ij} & \\ \rho_{e1ij}\sigma_{e1ij}\sigma_{e1ji} & \sigma^2_{e1ji} \end{bmatrix} \right) \\
\begin{bmatrix} \eta_{tijk} \\ \eta_{tjik} \end{bmatrix} &\sim \mathrm{N}\left(\begin{bmatrix} 0 \\ 0 \end{bmatrix}, \begin{bmatrix} \sigma^2_{\eta ij} & \\ \rho_{\eta ij}\sigma_{\eta ij}\sigma\eta ji & \sigma^2_{\eta ji} \end{bmatrix} \right), \quad \text{for } t > 1
\end{aligned}
\tag{S2}
$$

This model allows constructiveness at the previous snapshot to influence constructiveness at the current snapshot – both the individual's own and their partner's. More precisely, it is the deviation from each individual's average when acting towards that other individual that is allowed to have an influence, rather than the absolute level of constructiveness – although as we showed in the paper, in fact we can interpret the coefficients equally well as the influence of the deviation or of the absolute level. This allows us to examine the extent to which an individual's level

of constructiveness persists from one snapshot to the next, and how they are influenced by their partner's behaviour at the previous snapshot.

At the first snapshot, the equation is identical to the longitudinal social relations model without dynamics. At subsequent snapshots, the equation is similar (and in particular still includes family, actor, partner and dyad-type effects), but the deviation e_{tijk} at each particular snapshot t from the average constructiveness shown by individual i towards individual j in family k is no longer simply the innovation (which is now denoted by η_{tijk}), but is now modelled as a function of individual i's deviation at snapshot $t-1$ from their average constructiveness towards individual j, individual j's deviation at snapshot $t-1$ from their average constructiveness towards individual i, and the innovation. The coefficients ϕ_{1ij} of individual i's deviation at the previous snapshot, which we refer to as the autoregressive effect (also known as the lag or self-lag), and the coefficients ϕ_{2ij} of individual j's deviation at the previous snapshot, which we refer to as the cross-lags or cross-lagged effects, are allowed to differ by dyad type (but are constant across families; they are fixed effects). The innovations η_{tijk} are modelled as having variances $\sigma^2_{\eta ij}$ that differ by dyad type and correlations ρ_{ij} between pairs of dyad types involving the same two family roles (i.e. between undirected dyad type), differing by those undirected dyad types. These variances and correlations are allowed to differ from the variances σ^2_{eij} and correlations ρ_{eij} between the innovations at the first snapshot, since we expect them to do so: some of what is counted as innovation variance at the first snapshot will be explained at subsequent snapshots by the individual's own and their partner's deviation in constructiveness at the previous snapshot.

S3.3 Treating the response as ordinal

The response is not measured on a continuous scale: there are only five categories available into which to classify participants' behaviour, and there is no guarantee that the difference between each category and the next is equivalent right across the scale. For example, it may be that moving from the second to the third category in some sense represents a greater or lesser increase in constructive behaviour than moving from the fourth to the fifth category. This implies we should model the response as ordinal, and indeed we do so in the model we focus on in the paper. Results for continuous models are also presented in this document, to allow comparison between the two approaches.

The ordinal version of the (longitudinal) social relations model without dynamics is given by

$$
\begin{aligned}
y_{tijk} &= \begin{cases} 2, & \phantom{\tau_3 <{}} y^*_{tijk} \le 0 \\ 3, & 0 < y^*_{tijk} \le \tau_2 \\ 4, & \tau_2 < y^*_{tijk} \le \tau_3 \\ 5, & \tau_3 < y^*_{tijk} \end{cases} \\
y^*_{tijk} &= \mu_{ij} + \theta_{ijk} + \eta_{tijk} \\
\theta_{ijk} &= f_k + a_{ik} + p_{jk} + d_{ijk}, \quad i \neq j \\
f_k &\sim \mathrm{N}(0, \sigma^2_f) \\
\begin{bmatrix} a_{ik} \\ p_{ik} \end{bmatrix} &\sim \mathrm{N}\left(\begin{bmatrix} 0 \\ 0 \end{bmatrix}, \begin{bmatrix} \sigma^2_{ai} & \\ \rho_{api}\sigma_{ai}\sigma_{pi} & \sigma^2_{pi} \end{bmatrix} \right) \\
\begin{bmatrix} d_{ijk} \\ d_{jik} \end{bmatrix} &\sim \mathrm{N}\left(\begin{bmatrix} 0 \\ 0 \end{bmatrix}, \begin{bmatrix} \sigma^2_{dij} & \\ \rho_{dij}\sigma_{dij}\sigma_{dji} & \sigma^2_{dji} \end{bmatrix} \right) \\
\begin{bmatrix} \eta_{tijk} \\ \eta_{tjik} \end{bmatrix} &\sim \mathrm{N}\left(\begin{bmatrix} 0 \\ 0 \end{bmatrix}, \begin{bmatrix} 1 & \\ \rho_{\eta ij} & 1 \end{bmatrix} \right)
\end{aligned}
\tag{S3}
$$

We conceptualise constructiveness as existing as a continuous variable y^* which we are unable to measure directly,

with our ordinal observed response y determined by the value of y^* via a set of thresholds: when the value of the unobserved y^* lies beneath the first threshold τ_1, our observed response y takes on its lowest value of 2; when the value of y^* lies between τ_1 and the second threshold τ_2, our observed response takes on the next highest value, 3; when the value of y^* lies between τ_2 and the third threshold τ_3, our observed response takes on the next highest value, 4; and when the value of y^* lies above τ_3, the highest threshold, our observed response takes on the next highest value, 5, which is also its highest value. The value of τ_2 and of τ_3 are estimated when we fit the model, but we fix the value of τ_1 at 0 in order to make sure the model is identified.

The unobserved response y^* is modelled exactly as our observed response is in (S1), except that we fix the innovation variances at 1, again to make sure the model is identified. We choose to fix the first threshold at 0 and the innovation variances at 1 because our model then corresponds to a probit link ordinal general linear model, allowing comparability between our results and those of other authors who use this model.

The ordinal version of the social relations model with dynamics, which is the model we focus on in the paper, is given by

$$
\begin{aligned}
y_{tijk} &= \begin{cases} 2, & y^*_{tijk} \leq 0 \\ 3, & 0 < y^*_{tijk} \leq \tau_2 \\ 4, & \tau_2 < y^*_{tijk} \leq \tau_3 \\ 5, & \tau_3 < y^*_{tijk} \end{cases} \\
y^*_{tijk} &= \mu_{ij} + \theta_{ijk} + e_{tijk} \\
e_{tijk} &= \phi_{1ij} e_{(t-1)ijk} + \phi_{2ij} e_{(t-1)jik} + \eta_{tijk}, \quad \text{for } t > 1 \\
\theta_{ijk} &= f_k + a_{ik} + p_{jk} + d_{ijk}, \quad \text{for } i \neq j \\
f_k &\sim \mathrm{N}(0, \sigma_f^2) \\
\begin{bmatrix} a_{ik} \\ p_{ik} \end{bmatrix} &\sim \mathrm{N}\left(\begin{bmatrix} 0 \\ 0 \end{bmatrix}, \begin{bmatrix} \sigma^2_{ai} & \\ \rho_{api}\sigma_{ai}\sigma_{pi} & \sigma^2_{pi} \end{bmatrix} \right) \\
\begin{bmatrix} d_{ijk} \\ d_{jik} \end{bmatrix} &\sim \mathrm{N}\left(\begin{bmatrix} 0 \\ 0 \end{bmatrix}, \begin{bmatrix} \sigma^2_{dij} & \\ \rho_{dij}\sigma_{dij}\sigma_{dji} & \sigma^2_{dji} \end{bmatrix} \right) \\
\begin{bmatrix} e_{1ijk} \\ e_{1jik} \end{bmatrix} &\sim \mathrm{N}\left(\begin{bmatrix} 0 \\ 0 \end{bmatrix}, \begin{bmatrix} 1 & \\ \rho_{e1ij} & 1 \end{bmatrix} \right) \\
\begin{bmatrix} \eta_{tijk} \\ \eta_{tjik} \end{bmatrix} &\sim \mathrm{N}\left(\begin{bmatrix} 0 \\ 0 \end{bmatrix}, \begin{bmatrix} 1 & \\ \rho_{\eta ij} & 1 \end{bmatrix} \right), \quad \text{for } t > 1
\end{aligned}
\tag{S4}
$$

It is a straightforward combination of the ordinal social relations model given by (S3) and the dynamic social relations model given by (S2): as in (S3), we conceptualise the existence of an unobserved continuous level of constructiveness y^* which is related to the observed level of constructiveness y in the same way as in (S3), again with the first threshold fixed to 0 and the other two to be estimated; and this unobserved continuous level of constructiveness y^* is modelled by the same equations as those for our observed response y in (S2), except that the innovation variances (both those for the first snapshot and those for subsequent snapshots) are fixed at 1.

We present the results of a further ordinal social relations model in this document: a model which includes autoregressive effects but not cross-lagged effects. This is so that we can see what difference to the other parameter estimates the autoregressive effects and cross-lagged effets make separately, rather than only being able to see what

difference they both make combined. This model is given by

$$
\begin{aligned}
y_{tijk} &= \begin{cases} 2, & \quad\quad\;\; y^*_{tijk} \leq 0 \\ 3, & 0 < y^*_{tijk} \leq \tau_2 \\ 4, & \tau_2 < y^*_{tijk} \leq \tau_3 \\ 5, & \tau_3 < y^*_{tijk} \end{cases} \\
y^*_{tijk} &= \mu_{ij} + \theta_{ijk} + e_{tijk} \\
e_{tijk} &= \phi_{1ij} e_{(t-1)ijk} + \eta_{tijk}, \quad \text{for } t > 1 \\
\theta_{ijk} &= f_k + a_{ik} + p_{jk} + d_{ijk}, \quad \text{for } i \neq j \\
f_k &\sim \mathrm{N}(0, \sigma^2_f) \\
\begin{bmatrix} a_{ik} \\ p_{ik} \end{bmatrix} &\sim \mathrm{N}\left(\begin{bmatrix} 0 \\ 0 \end{bmatrix}, \begin{bmatrix} \sigma^2_{ai} & \\ \rho_{api}\sigma_{ai}\sigma_{pi} & \sigma^2_{pi} \end{bmatrix} \right) \\
\begin{bmatrix} d_{ijk} \\ d_{jik} \end{bmatrix} &\sim \mathrm{N}\left(\begin{bmatrix} 0 \\ 0 \end{bmatrix}, \begin{bmatrix} \sigma^2_{dij} & \\ \rho_{dij}\sigma_{dij}\sigma_{dji} & \sigma^2_{dji} \end{bmatrix} \right) \\
\begin{bmatrix} e_{1ijk} \\ e_{1jik} \end{bmatrix} &\sim \mathrm{N}\left(\begin{bmatrix} 0 \\ 0 \end{bmatrix}, \begin{bmatrix} 1 & \\ \rho_{e1ij} & 1 \end{bmatrix} \right) \\
\begin{bmatrix} \eta_{tijk} \\ \eta_{tjik} \end{bmatrix} &\sim \mathrm{N}\left(\begin{bmatrix} 0 \\ 0 \end{bmatrix}, \begin{bmatrix} 1 & \\ \rho_{\eta ij} & 1 \end{bmatrix} \right), \quad \text{for } t > 1
\end{aligned}
\tag{S5}
$$

S3.4 Adding a (linear) time trend

The final model for which we present results in this document is one which adds linear time trends to the model we focus on in the paper (i.e. to (S4)). We fit this model because we cannot assume that the average level of constructiveness with which one individual behaves towards another remains constant throughout the duration of the task they work on: they may become less constructive as they become frustrated or tired, or more constructive as they feel they are making progress in the task. Since, however, it turns out that adding the time trends does not make much difference to the other parameter estimates, and it is those rather than the time trends themselves which are of interest, for simplicity we focus in the paper on the model which does not include time trends. The model with

time trends is given by:

$$
\begin{aligned}
y_{tijk} &= \begin{cases} 2, & y^*_{tijk} \leq 0 \\ 3, & 0 < y^*_{tijk} \leq \tau_2 \\ 4, & \tau_2 < y^*_{tijk} \leq \tau_3 \\ 5, & \tau_3 < y^*_{tijk} \end{cases} \\
y^*_{tijk} &= \mu_{ij} + \alpha_{2ij} t' + \theta_{ijk} + e_{tijk} \\
e_{tijk} &= \phi_{1ij} e_{(t-1)ijk} + \phi_{2ij} e_{(t-1)jik} + \eta_{tijk}, \quad \text{for } t > 0 \\
\theta_{ijk} &= f_k + a_{ik} + p_{jk} + d_{ijk}, \quad \text{for } i \neq j \\
f_k &\sim \mathrm{N}(0, \sigma^2_f) \qquad \text{(S6)} \\
\begin{bmatrix} a_{ik} \\ p_{ik} \end{bmatrix} &\sim \mathrm{N}\left(\begin{bmatrix} 0 \\ 0 \end{bmatrix}, \begin{bmatrix} \sigma^2_{ai} & \\ \rho_{api}\sigma_{ai}\sigma_{pi} & \sigma^2_{pi} \end{bmatrix} \right) \\
\begin{bmatrix} d_{ijk} \\ d_{jik} \end{bmatrix} &\sim \mathrm{N}\left(\begin{bmatrix} 0 \\ 0 \end{bmatrix}, \begin{bmatrix} \sigma^2_{dij} & \\ \rho_{dij}\sigma_{dij}\sigma_{dji} & \sigma^2_{dji} \end{bmatrix} \right) \\
\begin{bmatrix} e_{1ijk} \\ e_{1jik} \end{bmatrix} &\sim \mathrm{N}\left(\begin{bmatrix} 0 \\ 0 \end{bmatrix}, \begin{bmatrix} 1 & \\ \rho_{e1ij} & 1 \end{bmatrix} \right) \\
\begin{bmatrix} \eta_{tijk} \\ \eta_{tjik} \end{bmatrix} &\sim \mathrm{N}\left(\begin{bmatrix} 0 \\ 0 \end{bmatrix}, \begin{bmatrix} 1 & \\ \rho_{\eta ij} & 1 \end{bmatrix} \right), \quad \text{for } t > 0
\end{aligned}
$$

We have recentred time to create the new variable t', with $t' = 0$ at the first snapshot, so that the intercepts are interpretable as the average constructiveness at the first snapshot rather than at the snapshot immediately before the participants begin the task. However, we still use our original uncentred time variable t, with $t = 1$ for the first snapshot, to index the snapshots. The coefficient of time, α_{2ij}, is allowed to differ by dyad type, but not by individual dyad. (We label this α_{2ij}, rather than α_{0ij} or α_{1ij}, because we wish to reserve the earlier numbers for another use which will be discussed later.) While it would be interesting to fit a random slope model which allows a different trajectory for each individual dyad, varying around an average trajectory for each dyad type, this would greatly complicate the estimation due to the need to reparameterise the dynamic models in order to fit them (as explained in Section S4), a task which becomes prohibitively complex for a growth trajectory model. Similarly, while it might be interesting, and plausible on substantive grounds, to fit higher order polynomial trajectories, this also greatly complicates the reparameterisation. We thus simply estimate the average linear increase or decrease in constructiveness from one snapshot to the next across all dyads of a particular type.

S3.5 Calculating additional results

Since we use MCMC estimation, we have a chain of estimates for each parameter, and thus we can, using these chains, find a credible interval as well as a point estimate for any function of the parameters which may be of interest, by evaluating this function at every stored iteration and then taking quantiles of the resulting chain of function evaluations. We could find a point estimate by evaluating the function using the point estimate of each parameter, but instead do so by taking the mean of the chain of function evaluations.

In the article, we present the proportions of the total dyad type variances accounted for by each component of variation. The variance partitioning coefficients, the total dyad type variances themselves, the correlations between each pair of dyad types, the correlation between the response at two consecutive snapshots (both for the same dyad type and for a pair of individuals with actor and partner roles reversed at one snapshot compared to the next), and the differences between various model parameters are also of interest and are presented in Section S5.3 for the model

we focus on in the paper (i.e. the ordinal dynamic social relations model, given by (S4)). These are all calculated using the saved parameter chains.

The variance partitioning coefficients are the proportion of the (unexplained) variance in the response accounted for by each component of variation. For the model we focus on in the paper, we calculate these for the underlying continuous response y^* rather than for the observed response y, and only at snapshots subsequent to the first. The total unexplained variance in y^*_{tijk} (at snapshots after the first) is given by

$$\sigma^2_f + \sigma^2_{ai} + \sigma^2_{pj} + \sigma^2_{dij} + \sigma^2_{\eta ij}$$

Since this is a function of i and j, it will differ by dyad type, as do the VPCs, given by

$$\frac{\sigma^2_f}{\sigma^2_f + \sigma^2_{ai} + \sigma^2_{pj} + \sigma^2_{dij} + \sigma^2_{\eta ij}}$$

for the family component,

$$\frac{\sigma^2_{ai}}{\sigma^2_f + \sigma^2_{ai} + \sigma^2_{pj} + \sigma^2_{dij} + \sigma^2_{\eta ij}}$$

for the actor component,

$$\frac{\sigma^2_{pj}}{\sigma^2_f + \sigma^2_{ai} + \sigma^2_{pj} + \sigma^2_{dij} + \sigma^2_{\eta ij}}$$

for the partner component,

$$\frac{\sigma^2_{dij}}{\sigma^2_f + \sigma^2_{ai} + \sigma^2_{pj} + \sigma^2_{dij} + \sigma^2_{\eta ij}}$$

for the dyad-type (or relationship) component, and

$$\frac{\sigma^2_{\eta ij}}{\sigma^2_f + \sigma^2_{ai} + \sigma^2_{pj} + \sigma^2_{dij} + \sigma^2_{\eta ij}}$$

for the innovation component.

The total (unexplained) dyad type variance is the variance of θ_{ijk}, and is given by

$$\sigma^2_f + \sigma^2_{ai} + \sigma^2_{pj} + \sigma^2_{dij}$$

The proportion accounted for by the family is given by

$$\frac{\sigma^2_f}{\sigma^2_f + \sigma^2_{ai} + \sigma^2_{pj} + \sigma^2_{dij}}$$

the proportion accounted for the actor by

$$\frac{\sigma^2_{ai}}{\sigma^2_f + \sigma^2_{ai} + \sigma^2_{pj} + \sigma^2_{dij}}$$

the proportion accounted for by the partner by

$$\frac{\sigma^2_{pj}}{\sigma^2_f + \sigma^2_{ai} + \sigma^2_{pj} + \sigma^2_{dij}}$$

and the proportion accounted for by the dyad type (or relationship) by

$$\frac{\sigma^2_{dij}}{\sigma^2_f + \sigma^2_{ai} + \sigma^2_{pj} + \sigma^2_{dij}}$$

Table S2: Different types of within-family covariance for the longitudinal social relations model

Type	Description	Covariance	Example $(ij, i'j')$
A	Within-person at different t<br>$i' = i,\ j' = j$	$\sigma_f^2 + \sigma_{ai}^2 + \sigma_{pj}^2 + \sigma_{dij}^2$	(MF, MF)
B1	Same dyad with actor-partner roles reversed at same t<br>$i' = j,\ j' = i$	$\sigma_f^2 + \rho_{api}\sigma_{ai}\sigma_{pi} + \rho_{apj}\sigma_{aj}\sigma_{pj} + \rho_{dij}\sigma_{dij}\sigma_{dji} + \rho_{\eta ij}\sigma_{\eta ij}\sigma\eta ji$	(MF, FM)
B2	Same dyad with actor-partner roles reversed at different t<br>$i' = j,\ j' = i$	$\sigma_f^2 + \rho_{api}\sigma_{ai}\sigma_{pi} + \rho_{apj}\sigma_{aj}\sigma_{pj} + \rho_{dij}\sigma_{dij}\sigma_{dji}$	(MF, FM)
C	Different dyads with same actor<br>$i' = i,\ j' \neq j$	$\sigma_f^2 + \sigma_{ai}^2$	(MF, MS_1)
D	Different dyad with same partner<br>$i' \neq i,\ j' = j$	$\sigma_f^2 + \sigma_{pj}^2$	(MF, S_1F)
E	Different dyads sharing one person who is actor in one and partner in the other<br>$i' \neq i,\ j' = i$	$\sigma_f^2 + \rho_{api}\sigma_{ai}\sigma_{pi}$	(MF, S_1M)
F	Different dyads with different people<br>$i' \neq i,\ i' \neq j,\ j' \neq i,\ j' \neq j$	σ_f^2	(MF, S_1S_2)

For the longitudinal social relations model (without dynamics), the within-family covariances $\text{cov}(y_{tijk}, y_{t'i'j'k})$ are given in Table S2. For the dynamic social relations model, covariances A and C–F are the same as shown in Table S2, but for type B, the expressions are more complicated and involve ϕ_{1ij}, ϕ_{2ij}, ϕ_{1ji}, and ϕ_{2ji}.

The correlation between the responses at consecutive snapshots is given by

$$\frac{\sigma_f^2 + \sigma_{ai}^2 + \sigma_{pj}^2 + \sigma_{dij}^2 + \phi_{1ij}\frac{\sigma_{\eta ij}^2}{1-\phi_{1ij}^2-\phi_{2ij}^2}}{\sigma_f^2 + \sigma_{ai}^2 + \sigma_{pj}^2 + \sigma_{dij}^2 + \frac{\sigma_{\eta ij}^2}{1-\phi_{1ij}^2-\phi_{2ij}^2}}$$

S4 Estimation

All models are fitted in JAGS, called from R using runjags (ordinal response models) or rjags (continuous response models). Data preparation and preparation of results (including calculation of additional results as described in Section S3.5) are carried out in R.

S4.1 Parameterisation for the continuous models

As shown in the paper, the dynamic social relations model can be rewritten as a dynamic panel model:

$$
\begin{aligned}
y_{1ijk} &= \mu_{ij} + f_k + a_{ik} + p_{jk} + d_{ijk} + e_{1ijk} \\
y_{tijk} &= \beta_{0ij} + \beta_{1ij} y_{(t-1)ijk} + \beta_{2ij} y_{(t-1)jik} + \bar{f}_{ijk} + \bar{a}_{ijk} + \bar{p}_{jik} + \bar{d}_{ijk} + \epsilon_{tijk}, \quad \text{for } t > 1
\end{aligned}
$$

$$
\begin{aligned}
\beta_{0ij} &= (1 - \phi_{1ij})\mu_{ij} - \phi_{2ij}\mu_{ji} \\
\beta_{1ij} &= \phi_{1ij} \\
\beta_{2ij} &= \phi_{2ij}
\end{aligned}
$$

$$
\begin{aligned}
\bar{f}_{ijk} &= (1 - \phi_{1ij} - \phi_{2ij}) f_k \\
\bar{a}_{ijk} &= (1 - \phi_{1ij}) a_{ik} - \phi_{2ij} a_{jk} \\
\bar{p}_{ijk} &= (1 - \phi_{1ji}) p_{ik} - \phi_{2ji} p_{ik} \\
\bar{d}_{ijk} &= (1 - \phi_{1ij}) d_{ijk} - \phi_{2ij} d_{jik} \\
\epsilon_{tijk} &= \eta_{tijk}
\end{aligned}
$$

It is ultimately the parameters of the dynamic social relations model that we model: we specify distributions with associated parameters to be estimated (as in the equation shown above for the dynamic social relations model) for f_k, a_{ik}, p_{ik}, d_{ijk} and d_{jik} rather than for $\bar{f}_{ijk}$, $\bar{a}_{ijk}$, $\bar{p}_{ijk}$, and $\bar{d}_{ijk}$, and we specify priors for the parameters of the distributions of these random effects, and for μ_{ij} rather than β_{0ij}. (As β_{1ij}, β_{2ij}, ϵ_{tijk} and ϵ_{tjik} are identical to ϕ_{1ij}, ϕ_{2ij}, η_{tijk} and η_{tjik} respectively, it is ambiguous whether we are modelling the distribution of ϵ_{tijk} and ϵ_{tjik} or of η_{tijk} and η_{tjik}, and whether we are specifying priors for the parameters of the distribution of ϵ_{tijk} and ϵ_{tjik} or the parameters of the distribution of η_{tijk} and η_{tjik}, for β_{1ij} or ϕ_{1ij}, and for β_{2ij} or ϕ_{2ij}.) However, rather than specifying y_{tijk} (for $t > 1$) directly as a function of μ_{ij}, f_k, a_{ik}, p_{jk} and d_{ijk} (and ϕ_{1ij}, ϕ_{2ij} and η_{tijk}) as in the equation for the dynamic social relations model, we specify y_{tijk} as a function of β_{0ij}, $\bar{f}_{ijk}$, $\bar{a}_{ijk}$, $\bar{p}_{ijk}$ and $\bar{d}_{ijk}$ (and β_{1ij} aka ϕ_{1ij}, β_{2ij} aka ϕ_{2ij} and ϵ_{tijk} aka η_{tijk}), and specify these in turn as functions of μ_{ij}, μ_{ji}, f_k, a_{ik}, a_{jk}, p_{jk}, p_{ik}, d_{ijk} and d_{jik} (and ϕ_{1ij} aka β_{1ij} and ϕ_{2ij} aka β_{2ij}), as shown in the equation for the dynamic panel model. We do this because JAGS can better handle a reference in the equation for the response to responses at the previous snapshot (datapoints) than to innovations at the previous snapshot (model estimates).

The equation for the dynamic social relations model shows variances and covariances as the parameters to be estimated for the distributions of f_k, a_{ik}, a_{jk}, p_{jk}, p_{ik}, d_{ijk}, d_{jik}, η_{tijk} and η_{tjik}. However, due to the way that JAGS works, we instead estimate a precision matrix for each distribution, then invert these to get the variances and covariances.

We specify y_{1ijk} as shown in the first line of the equation for the dynamic panel model, so unlike for $t > 1$, this is specified directly in terms of μ_{ij}, f_k, a_{ik}, p_{jk} and d_{ijk}. The precision matrix (and thus also the variance-covariance matrix) for the distribution of the η_{1ijk} and η_{1jik} is allowed to differ from that for η_{tijk} and η_{tjik} when $t > 1$.

The parameterisation for the non-dynamic social relations model at all snapshots is as for the dynamic social relations model at $t = 1$: y_{tijk} is specified directly in terms of μ_{ij}, f_k, a_{ik}, p_{jk} and d_{ijk} (and η_{tijk}); and we estimate precision matrices for the distributions of the random effects with the variances and covariances derived by inverting these.

S4.2 Parameterisation for the ordinal models

As with the dynamic continuous model, we reparameterise the dynamic ordinal models as dynamic panel models to fit them in JAGS. The relationship between the parameters of the dynamic panel model and those of the dynamic social relations model is exactly the same as for the dynamic continuous model except for in the model with linear time trends. We provide the details of the parameterisation of the latter as a dynamic panel model in Section S4.3.

As described in Section S3.3, in order to treat the response as ordinal, we specify a model with an unobserved continuous response, y^*_{tijk}, and a relation between this and the observed y_{tijk} whereby the value of y_{tijk} is determined by which pair of consecutive thresholds τ_c and τ_{c+1} from among a set to be estimated y^*_{tijk} falls between, or whether it falls below the first or above the last. In order to render the model identifiable, the first threshold is set at 0 and the variance of each η_{tijk} is fixed at 1 (with correlations between ηs for dyads involving the same individuals in reversed roles still freely estimated); and in order to make sure the thresholds are correctly ordered (i.e. we always have $\tau_2 \geq \tau_1$ and $\tau_3 \geq \tau 2$) we specify that τ_2 is the sum of τ_1 and a parameter $\delta_{\tau 2}$ specified as having an exponential prior distribution so that it is constrained to be positive, and similarly τ_3 is the sum of τ_2 and a parameter $\delta_{\tau 3}$ with an exponential prior distribution. In contrast to when we treat the response as continuous, we calculate the precision matrices of the distributions of η_{tijk} and η_{tjik} as the inverses of their variance-covariance matrices, rather than calculating their variance-covariance matrices as the inverses of their precision matrices. This is so that we can estimate the correlations while fixing the variances to 1: we specify that the covariances (which are the correlations, since the variances are set to 1) are a hyperbolic tanh (aka Fisher transformation) of a parameter ψ_1 (for $t = 1$) or ψ_2 (for $t > 1$) to be estimated (with a Normal prior), which means that they are constrained to lie between -1 and 1 as they should. As for the continuous models, we allow the covariances between η_{tijk} and η_{tjik} to differ between $t = 1$ and $t >$ (the variances are of course equal for all t, being set to 1).

We initially fitted a model which was the same as the dynamic social relations model we fitted, except for the changes just described in estimating the parameters for the distributions of the η_{tijk} and η_{tjik}, and for y_{tijk} being replaced by y^*_{tijk}, with the relation between y_{tijk} and y^*_{tijk} as an additional part of the model (recall that, as the category 1 was not used at all by raters, y_{tijk} ranges[1] from 2 to 5):

[1]We show in this equation the mapping between the original values of y_{tijk} in the dataset, which range from 2 to 5, and y^*_{tijk}, but in fact we used a transformed version of y_{tijk} in the modelling, which was created by subtracting 2 from the values of y_{tijk} and which accordingly ranged from 0 to 3. This was because JAGS requires the categories of an ordinal response to be consecutively numbered starting at 0. Throughout the document (e.g. in Table S6) we give details in terms of the orignal values of y_{tijk}.

$$y_{tijk} = \begin{cases} 2, & y^*_{tijk} \leq 0 \\ 3, & 0 < y^*_{tijk} \leq \tau_2 \\ 4, & \tau_2 < y^*_{tijk} \leq \tau_3 \\ 5, & \tau_3 < y^*_{tijk} \end{cases}$$

$$\begin{aligned} y^*_{1ijk} &= \mu_{ij} + f_k + a_{ik} + p_{jk} + d_{ijk} + e_{1ijk} \\ y^*_{tijk} &= \beta_{0ij} + \beta_{1ij} y^*_{(t-1)ijk} + \beta_{2ij} y^*_{(t-1)jik} + \bar{f}_{ijk} + \bar{a}_{ijk} + \bar{p}_{jik} + \bar{d}_{ijk} + \epsilon_{tijk}, \quad \text{for } t > 1 \end{aligned}$$

$$\begin{aligned} \beta_{0ij} &= (1 - \phi_{1ij})\mu_{ij} - \phi_{2ij}\mu_{ji} \\ \beta_{1ij} &= \phi_{1ij} \\ \beta_{2ij} &= \phi_{2ij} \\ \bar{f}_{ijk} &= (1 - \phi_{1ij} - \phi_{2ij}) f_k \\ \bar{a}_{ijk} &= (1 - \phi_{1ij}) a_{ik} - \phi_{2ij} a_{jk} \\ \bar{p}_{ijk} &= (1 - \phi_{1ji}) p_{jk} - \phi_{2ji} p_{ik} \\ \bar{d}_{ijk} &= (1 - \phi_{1ij}) d_{ijk} - \phi_{2ij} d_{jik} \\ \epsilon_{tijk} &= \eta_{tijk}, \quad \text{for } t > 1 \end{aligned}$$

$$\begin{aligned} f_k &\sim \mathrm{N}(0, \sigma^2_f) \\ \begin{bmatrix} a_{ik} \\ p_{ik} \end{bmatrix} &\sim \mathrm{N}\left(\begin{bmatrix} 0 \\ 0 \end{bmatrix}, \begin{bmatrix} \sigma^2_{ai} & \\ \rho_{api}\sigma_{ai}\sigma_{pi} & \sigma^2_{pi} \end{bmatrix} \right) \\ \begin{bmatrix} d_{ijk} \\ d_{jik} \end{bmatrix} &\sim \mathrm{N}\left(\begin{bmatrix} 0 \\ 0 \end{bmatrix}, \begin{bmatrix} \sigma^2_{dij} & \\ \rho_{dij}\sigma_{dij}\sigma_{dji} & \sigma^2_{dji} \end{bmatrix} \right) \\ \begin{bmatrix} e_{1ijk} \\ e_{1jik} \end{bmatrix} &\sim \mathrm{N}\left(\begin{bmatrix} 0 \\ 0 \end{bmatrix}, \begin{bmatrix} 1 & \\ \rho_{e1ij} & 1 \end{bmatrix} \right) \\ \begin{bmatrix} \eta_{tijk} \\ \eta_{tjik} \end{bmatrix} &\sim \mathrm{N}\left(\begin{bmatrix} 0 \\ 0 \end{bmatrix}, \begin{bmatrix} 1 & \\ \rho_{\eta ij} & 1 \end{bmatrix} \right), \quad \text{for } t > 1 \end{aligned}$$

However, the chains showed poor mixing for the fixed intercept and threshold parameters. Accordingly, in the model whose results we present in the paper, we reparameterised the fixed intercepts μ_{ij}, so that instead of estimating a completely separate parameter for each dyad type, we estimate an overall intercept α_0 common to all dyad types and a deviation α_{1ij} from this for each dyad type (other than mothers acting towards fathers, which is set as the reference dyad type), with μ_{ij} then being calculated as $\alpha_0 + \alpha_{1ij}$. (We report estimates for μ_{ij} rather than for α_0 and α_{1ij} in the paper.) We kept the same parameterisation for the thresholds, but additionally calculated from these and the overall intercept thresholds κ_c of an alternative parameterisation, in which $\kappa_c = \tau_c - \alpha_0$, with the idea that if, as proved to be the case, the chains for the κ_c showed good mixing, then it wouldn't matter if those of the τ_c still showed poor mixing.

S4.3 Parameterisation for the model with a linear time trend

As mentioned in Section S3.4, we take $t' = t - 1$, rather than t, as our time variable for the linear trend, so that time is centred at the first snapshot and the intercepts can be interpreted as referring to constructiveness at this time. However, we do not actually create the variable t': we just put `(t-1)` rather than `t` into the model syntax in the time trend. (We still use t, not t', to index the observations.) Since we specify y^*_{1ijk} and y^*_{tijk} for $t > 1$ separately, and since $\alpha_{2ij}t'$ will in all cases equal 0 when $t = 1$ (since then $t' = 0$), for simplicity we only include the time trend

in the specification for y^*_{tijk} for $t > 1$, not in the specification for y^*_{1ijk}.

In order to reparameterise this model as a dynamic panel model, we note that (for $t > 1$)

$$e_{tijk} = y^*_{tijk} - \mu_{ij} - \alpha_{2ij}t' - \theta_{ijk}$$

and that

$$y^*_{tijk} = \mu_{ij} + \alpha'_{2ij} + \phi_{1ij}e_{(t-1)ijk} + \phi_{2ij}e_{(t-1)jik} + \theta_{ijk} + \eta_{tijk}$$

which means that

$$\begin{aligned} y^*_{tijk} &= \mu_{ij} + \alpha'_{2ij} + \phi_{1ij}(y^*_{(t-1)ijk} - \mu_{ij} - \alpha_{2ij}(t'-1) - \theta_{ijk}) \\ &\quad + \phi_{2ij}(y^*_{(t-1)jik} - \mu_{ji} - \alpha_{2ji}(t'-1) - \theta_{jik}) + \theta_{ijk} + \eta_{tijk} \\ \\ &= (1-\phi_{1ij})\mu_{ij} - \phi_{2ij}\mu_{ji} + \phi_{1ij}\alpha_{2ij} + \phi_{2ij}\alpha_{2ji} + ((1-\phi_{1ij})\alpha_{2ij} - \phi_{2ij}\alpha_{2ji})t' \\ &\quad + \phi_{1ij}y^*_{(t-1)ijk} + \phi_{2ij}y^*_{(t-1)ijk} + (1-\phi_{1ij})\theta_{ijk} - \phi_{2ij}\theta_{jik} + \eta_{tijk} \end{aligned}$$

This (together with the parameterisation of μ_{ij} as $\alpha_0 + \alpha_{1ij}$ which we retain in this model) gives us the following as the model we fit:

$$y_{tijk} = \begin{cases} 2, & y^*_{tijk} \le 0 \\ 3, & 0 < y^*_{tijk} \le \tau_2 \\ 4, & \tau_2 < y^*_{tijk} \le \tau_3 \\ 5, & \tau_3 < y^*_{tijk} \end{cases}$$

$$\begin{aligned} y^*_{1ijk} &= \mu_{ij} + f_k + a_{ik} + p_{jk} + d_{ijk} + e_{1ijk} \\ y^*_{tijk} &= \beta_{0ij} + \beta_{1ij}y^*_{(t-1)ijk} + \beta_{2ij}y^*_{(t-1)jik} + \beta_{3ij}(t-1) + \bar{f}_{ijk} + \bar{a}_{ijk} + \bar{p}_{jik} + \bar{d}_{ijk} + \epsilon_{tijk}, \quad \text{for } t > 1 \end{aligned}$$

$$\begin{aligned} \mu_{ij} &= \alpha_0 + \alpha_{1ij} \\ \beta_{0ij} &= (1-\phi_{1ij})\mu_{ij} + \phi_{2ij}\mu_{ji} + \phi_{1ij}\alpha_{2ij} + \phi_{2ij}\alpha_{2ji} \\ \beta_{1ij} &= \phi_{1ij} \\ \beta_{2ij} &= \phi_{2ij} \\ \beta_{3ij} &= (1-\phi_{1ij})\alpha_{2ij} - \phi_{2ij}\alpha_{2ji} \\ \bar{f}_{ijk} &= (1-\phi_{1ij}-\phi_{2ij})f_k \\ \bar{a}_{ijk} &= (1-\phi_{1ij})a_{ik} - \phi_{2ij}a_{jk} \\ \bar{p}_{ijk} &= (1-\phi_{1ji})p_{jk} - \phi_{2ji}p_{ik} \\ \bar{d}_{ijk} &= (1-\phi_{1ij})d_{ijk} - \phi_{2ij}d_{jik} \\ \epsilon_{tijk} &= \eta_{tijk} \end{aligned}$$

$$\begin{aligned} f_k &\sim \mathrm{N}(0, \sigma_f^2) \\ \begin{bmatrix} a_{ik} \\ p_{ik} \end{bmatrix} &\sim \mathrm{N}\left(\begin{bmatrix} 0 \\ 0 \end{bmatrix}, \begin{bmatrix} \sigma^2_{ai} & \\ \rho_{api}\sigma_{ai}\sigma_{pi} & \sigma^2_{pi} \end{bmatrix} \right) \\ \begin{bmatrix} d_{ijk} \\ d_{jik} \end{bmatrix} &\sim \mathrm{N}\left(\begin{bmatrix} 0 \\ 0 \end{bmatrix}, \begin{bmatrix} \sigma^2_{dij} & \\ \rho_{dij}\sigma_{dij}\sigma_{dji} & \sigma^2_{dji} \end{bmatrix} \right) \\ \begin{bmatrix} e_{1ijk} \\ e_{1jik} \end{bmatrix} &\sim \mathrm{N}\left(\begin{bmatrix} 0 \\ 0 \end{bmatrix}, \begin{bmatrix} 1 & \\ \rho_{e1ij} & 1 \end{bmatrix} \right) \\ \begin{bmatrix} \eta_{tijk} \\ \eta_{tjik} \end{bmatrix} &\sim \mathrm{N}\left(\begin{bmatrix} 0 \\ 0 \end{bmatrix}, \begin{bmatrix} 1 & \\ \rho_{\eta ij} & 1 \end{bmatrix} \right), \quad \text{for } t > 1 \end{aligned}$$

S4.4 Priors

Table S3 shows the prior used for each parameter (in models where that parameter appears and has a prior specified). "Parameter" in the first column refers to each model parameter which has a prior distribution specified, "Value 1" in the third column refers to the first (or only) parameter of the distribution (mean for Normal distributions; rate for exponential distributions; shape for gamma distributions; scale matrix for Wishart distributions), and "Value 2" in the fourth column refers to the second parameter of the distribution for distributions which have a second parameter (precision for Normal distributions; rate for gamma distributions; degrees of freedom for Wishart distributions). The priors do not specify correlation between any of the parameters to be estimated: they are independent distributions.

Table S3: Prior distributions specified in models

Parameter	Distribution	Value 1	Value 2
$\delta_{\tau 2}$	Exponential	1	
$\delta_{\tau 3}$	Exponential	1	
α_0	Normal	0	0.001
α_{1ij}	Normal	0	0.001
μ_{ij}	Normal	0	0.0001
α_{2ij}	Normal	0	0.001
ϕ_{1ij}	Normal	0	0.001
ϕ_{2ij}	Normal	0	0.001
Precision of f_k	Gamma	0.001	0.001
Precision matrix for a_{ik} and p_{ik}	Wishart	$\begin{bmatrix} 1 & 0 \\ 0 & 1 \end{bmatrix}$	2
Precision matrix for d_{ijk} and d_{jik}	Wishart	$\begin{bmatrix} 1 & 0 \\ 0 & 1 \end{bmatrix}$	2
Precision matrix for η_{1ijk} and η_{1jik}	Wishart	$\begin{bmatrix} 1 & 0 \\ 0 & 1 \end{bmatrix}$	2
Precision matrix for η_{tijk} and η_{tijk}, $t > 1$	Wishart	$\begin{bmatrix} 1 & 0 \\ 0 & 1 \end{bmatrix}$	2
ψ_1	Normal	0	0.001
ψ_2	Normal	0	0.001

S4.5 Starting values

For the continuous models, we just run one chain (for each parameter) per model, with starting values that we chose. This was felt to be enough since they were being run only as robustness checks, not as the model of interest for the paper. For the ordinal response models, we ran five chains, with starting values randomly selected for most parameters, so that we could be confident that we had explored the range of values for each parameter with greatest probability density.

For the continuous response models, the starting values for each paramter (for models including that parameter) were as follows:

Table S4: Starting values specified in continuous response models

Parameter	Starting value
μ_{ij}	0
ϕ_{1ij}	0
ϕ_{2ij}	0
Precision of f_k	2
Precision matrix for a_{ik} and p_{ik}	$\begin{bmatrix} 2 & 0 \\ 0 & 2 \end{bmatrix}$
Precision matrix for d_{ijk} and d_{jik}	$\begin{bmatrix} 2 & 0 \\ 0 & 2 \end{bmatrix}$
Precision matrix for η_{1ijk} and η_{1jik}	$\begin{bmatrix} 2 & 0 \\ 0 & 2 \end{bmatrix}$
Precision matrix for η_{tijk} and η_{tijk}, $t > 1$	$\begin{bmatrix} 2 & 0 \\ 0 & 2 \end{bmatrix}$

For the ordinal response models, Table S5 gives the details of how starting values were randomly drawn for each parameter. For the precision matrices for a_{ik} and p_{ik} and for d_{ijk} and d_{jik}, the off-diagonal elements were fixed at 0, and the diagonal elements were determined, independently from each other, by drawing a value which was then multiplied by 2 (hence why the corresponding table entries are listed as "half of diagonal elements" of these matrices).

Table S5: Details of the drawing of starting values for the ordinal response models

Parameter	Distribution	Value 1	Value 2
$\delta_{\tau 2}$	Normal	1.6	0.01
$\delta_{\tau 3}$	Normal	2.2	0.01
α_0	Normal	2	0.2
α_{1ij}	Normal	0	0.5
α_{2ij}	Normal	0	0.001
ϕ_{1ij}	Normal	0	0.2
ϕ_{2ij}	Normal	0	0.2
Precision of f_k	Gamma	5	1
Half of diagonal elements of precision matrix for a_{ik} and p_{ik}	Gamma	5	1
Half of diagonal elements of precision matrix for d_{ijk} and d_{jik}	Gamma	5	1
ψ_1	Normal	0	0.2
ψ_2	Normal	0	0.2

Besides these random drawings, fixed starting values were specified for the latent response y^*_{tijk}, based deterministically on y_{tijk}:

Table S6: Starting values for y^*_{tijk}: correspondence with y_{tijk}

y_{tijk}	Starting value of y^*_{tijk}
2	-0.5
3	1
4	2.5
5	4

S4.6 Judging convergence

Stochastic convergence was judged by examining trajectory plots for signs of trending or autocorrelation, looking at effective sample sizes (ESSs), and (for the ordinal response models) checking that Gelman-Rubin potential scale reduction factors (PSRFs) were close to 1. (We could not calculate PSRFs for the continuous response models, because we only ran one chain for these.)

For the model of interest (i.e. the ordinal response dynamic social relations model presented in the paper), we specified a relatively short burn-in when running the model, of $50,000$ iterations (following an adaptation phase of $1,000$ iterations), followed by $200,000$ monitoring iterations for each chain. However, on examining the trajectories, it was apparent that for the thresholds τ_2 and τ_3 there was an upwards trend as iteration proceeded, with the increase relatively steep to start with and becoming shallower and shallower until by the end of the monitoring chain it was almost flat. Accordingly, we increased the burn-in by discarding the first $150,000$ monitoring iterations of each chain, to give a burn-in of $200,000$ iterations and a monitoring chain of $50,000$ iterations. Although τ_2 and τ_3 showed a very slight upward trend even over the course of these iterations at the end of the original chains that we kept, and still had low ESSs (of 16 each) and large PSRFs (of3.5 and 1.6 respectively) we considered that this was acceptable given that the κ_c of the alternative threshold parameterisation did not show any trend, and that the τ_c were not parameters of interest (and are not included in the results table in the paper).

Since the trajectories showed similar behaviour for the other ordinal response models, we did the same for them, also taking a monitoring chain of $50,000$ iterations after a burn-in of $200,000$ iterations and an adaptation phase of $1,000$ iterations.

Mixing was much better for the continuous response models. We ran the non-dynamic continuous response model for $40,000$ monitoring iterations and the dynamic continuous response model for $150,000$ iterations, both with a burn-in of $5,000$ iterations.

Table S7 shows the smallest ESS and (for the ordinal response models) the PSRF that differs most from 1 among those for all parameters in the JAGS output (other than τ_2 and τ_3) for each model. (The JAGS output includes all parameters appearing in Tables S8 or S37, provided they are included in the model in question, and additionally, for models that include them, α_0, α_{1ij}, and β_{0ij}.)

Table S7: Minimum ESSs and PSRFs furthest from 1 for each model

	ESS	Parameter (ESS)	PSRF	Parameter(PSRF)
Continuous non-dynamic	376	σ_f^2		
Continuous dynamic	1,358	σ_f^2		
Ordinal non-dynamic	5,280	κ_3	1.045	μ (S_2▸F)
Ordinal dynamic	1,588	κ_3	1.042	κ_3
Ordinal dynamic with time trend	7,438	κ_3	1.052	μ (M▸S_2)

S5 Results

S5.1 Treatment of the response as continuous or ordinal

In this section, we present results for continuous and ordinal social relations models, with and without dynamics, side by side to allow an examination of how similar the results are when treating the response in each way.

Table S8: Results of Continuous SRM, Ordinal SRM, Continuous DSRM, and Ordinal DSRM

	Continuous SRM			Ordinal SRM			Continuous DSRM			Ordinal DSRM		
	Mean	Quantiles		Mean	Quantiles		Mean	Quantiles		Mean	Quantiles	
		2.5%	97.5%		2.5%	97.5%		2.5%	97.5%		2.5%	97.5%
τ_1				0.000	0.000	0.000				0.000	0.000	0.000
τ_2				1.808	1.775	1.840				1.791	1.759	1.812
τ_3				4.429	4.376	4.481				4.386	4.322	4.443
μ (M▸F)	3.660	3.597	3.723	2.266	2.138	2.395	3.663	3.600	3.726	2.244	2.115	2.372
μ (F▸M)	3.558	3.493	3.623	2.032	1.906	2.161	3.558	3.494	3.622	2.015	1.886	2.146
μ (M▸S_1)	3.786	3.725	3.848	2.621	2.483	2.757	3.788	3.725	3.850	2.596	2.460	2.735
μ (S_1▸M)	3.131	3.068	3.195	1.151	1.029	1.273	3.129	3.064	3.193	1.139	1.017	1.260
μ (M▸S_2)	3.788	3.729	3.846	2.616	2.488	2.746	3.787	3.729	3.846	2.589	2.460	2.719
μ (S_2▸M)	3.317	3.256	3.377	1.531	1.413	1.650	3.315	3.256	3.375	1.516	1.400	1.632
μ (F▸S_1)	3.863	3.795	3.930	2.803	2.653	2.953	3.863	3.793	3.931	2.774	2.625	2.923
μ (S_1▸F)	3.296	3.232	3.361	1.480	1.363	1.595	3.297	3.231	3.361	1.471	1.352	1.590
μ (F▸S_2)	3.700	3.633	3.765	2.391	2.254	2.527	3.699	3.632	3.766	2.369	2.234	2.505
μ (S_2▸F)	3.333	3.272	3.394	1.548	1.439	1.659	3.331	3.269	3.393	1.531	1.420	1.643
μ (S_1▸S_2)	3.269	3.213	3.325	1.421	1.316	1.526	3.275	3.217	3.334	1.416	1.307	1.525
μ (S_2▸S_1)	3.381	3.320	3.443	1.658	1.540	1.778	3.381	3.320	3.444	1.648	1.529	1.766
ϕ_1 (M▸F)							0.011	−0.047	0.069	0.068	−0.025	0.162
ϕ_1 (F▸M)							0.120	0.061	0.178	0.202	0.112	0.293
ϕ_1 (M▸S_1)							0.089	0.039	0.139	0.095	0.029	0.161
ϕ_1 (S_1▸M)							0.145	0.096	0.195	0.224	0.150	0.297
ϕ_1 (M▸S_2)							0.091	0.043	0.139	0.102	0.033	0.171
ϕ_1 (S_2▸M)							0.162	0.114	0.210	0.237	0.168	0.307
ϕ_1 (F▸S_1)							0.058	−0.002	0.119	0.085	−0.006	0.175
ϕ_1 (S_1▸F)							0.135	0.076	0.194	0.208	0.121	0.296
ϕ_1 (F▸S_2)							0.073	0.015	0.131	0.108	0.023	0.192
ϕ_1 (S_2▸F)							0.051	−0.006	0.109	0.120	0.030	0.209
ϕ_1 (S_1▸S_2)							0.089	0.024	0.151	0.143	0.051	0.233
ϕ_1 (S_2▸S_1)							0.094	0.030	0.158	0.164	0.067	0.262
ϕ_2 (M▸F)							−0.023	−0.078	0.032	−0.024	−0.113	0.066
ϕ_2 (F▸M)							0.010	−0.046	0.067	0.007	−0.078	0.092
ϕ_2 (M▸S_1)							−0.026	−0.078	0.027	−0.054	−0.135	0.027
ϕ_2 (S_1▸M)							0.001	−0.041	0.045	−0.009	−0.065	0.046

(cont.)

(cont.)

	Continuous SRM			Ordinal SRM			Continuous DSRM			Ordinal DSRM		
	Mean	Quantiles		Mean	Quantiles		Mean	Quantiles		Mean	Quantiles	
		2.5%	97.5%		2.5%	97.5%		2.5%	97.5%		2.5%	97.5%
ϕ_2 (M▸S_2)							0.035	−0.011	0.080	0.056	−0.014	0.127
ϕ_2 (S_2▸M)							0.048	0.000	0.094	0.045	−0.015	0.106
ϕ_2 (F▸S_1)							0.011	−0.048	0.067	0.016	−0.080	0.114
ϕ_2 (S_1▸F)							0.018	−0.040	0.076	0.001	−0.073	0.075
ϕ_2 (F▸S_2)							−0.010	−0.071	0.052	−0.016	−0.115	0.080
ϕ_2 (S_2▸F)							0.006	−0.043	0.057	0.002	−0.068	0.073
ϕ_2 (S_1▸S_2)							0.080	0.022	0.137	0.099	0.015	0.180
ϕ_2 (S_2▸S_1)							0.021	−0.042	0.085	0.028	−0.067	0.123
σ_f^2	0.002	0.000	0.006	0.013	0.000	0.057	0.002	0.000	0.006	0.031	0.001	0.111
σ_a^2 (M)	0.048	0.033	0.067	0.208	0.127	0.305	0.048	0.033	0.065	0.200	0.121	0.294
σ_a^2 (F)	0.045	0.031	0.063	0.195	0.123	0.286	0.045	0.031	0.062	0.183	0.110	0.273
σ_a^2 (S_1)	0.045	0.031	0.062	0.135	0.083	0.197	0.044	0.031	0.061	0.121	0.072	0.182
σ_a^2 (S_2)	0.040	0.028	0.054	0.111	0.066	0.167	0.039	0.027	0.054	0.101	0.059	0.157
σ_p^2 (M)	0.035	0.024	0.048	0.088	0.052	0.137	0.034	0.024	0.048	0.084	0.049	0.132
σ_p^2 (F)	0.030	0.021	0.041	0.064	0.039	0.098	0.029	0.021	0.041	0.061	0.037	0.094
σ_p^2 (S_1)	0.037	0.026	0.051	0.121	0.070	0.188	0.037	0.026	0.052	0.115	0.066	0.180
σ_p^2 (S_2)	0.034	0.024	0.046	0.092	0.054	0.142	0.034	0.024	0.047	0.087	0.050	0.136
σ_d^2 (M▸F)	0.056	0.037	0.082	0.168	0.088	0.281	0.055	0.036	0.080	0.157	0.081	0.266
σ_d^2 (F▸M)	0.052	0.034	0.076	0.134	0.070	0.225	0.051	0.033	0.076	0.122	0.063	0.210
σ_d^2 (M▸S_1)	0.083	0.056	0.117	0.488	0.329	0.674	0.080	0.053	0.113	0.451	0.300	0.630
σ_d^2 (S_1▸M)	0.107	0.076	0.146	0.408	0.277	0.568	0.098	0.067	0.138	0.350	0.223	0.504
σ_d^2 (M▸S_2)	0.076	0.051	0.107	0.421	0.278	0.591	0.073	0.048	0.103	0.386	0.243	0.557
σ_d^2 (S_2▸M)	0.093	0.064	0.127	0.390	0.263	0.542	0.084	0.056	0.118	0.315	0.193	0.463
σ_d^2 (F▸S_1)	0.066	0.042	0.097	0.332	0.190	0.513	0.065	0.041	0.096	0.296	0.161	0.472
σ_d^2 (S_1▸F)	0.055	0.035	0.081	0.139	0.073	0.233	0.053	0.034	0.078	0.119	0.063	0.204
σ_d^2 (F▸S_2)	0.060	0.039	0.089	0.249	0.134	0.400	0.058	0.037	0.087	0.218	0.110	0.362
σ_d^2 (S_2▸F)	0.052	0.034	0.076	0.122	0.067	0.202	0.051	0.034	0.075	0.115	0.062	0.191
σ_d^2 (S_1▸S_2)	0.048	0.031	0.069	0.108	0.059	0.178	0.047	0.031	0.068	0.096	0.052	0.161
σ_d^2 (S_2▸S_1)	0.072	0.047	0.103	0.247	0.141	0.377	0.067	0.044	0.096	0.201	0.106	0.327
ρ_{ap} (M)	0.156	−0.088	0.385	0.195	−0.149	0.503	0.161	−0.082	0.393	0.171	−0.188	0.485
ρ_{ap} (F)	0.080	−0.166	0.319	0.075	−0.265	0.400	0.076	−0.170	0.315	0.041	−0.301	0.374
ρ_{ap} (S_1)	0.223	−0.011	0.438	0.326	0.009	0.589	0.225	−0.009	0.442	0.302	−0.039	0.580

(cont.)

(cont.)

	Continuous SRM			Ordinal SRM			Continuous DSRM			Ordinal DSRM		
	Mean	Quantiles		Mean	Quantiles		Mean	Quantiles		Mean	Quantiles	
		2.5%	97.5%		2.5%	97.5%		2.5%	97.5%		2.5%	97.5%
ρ_{ap} (S_2)	0.276	0.052	0.477	0.407	0.105	0.646	0.268	0.042	0.476	0.360	0.025	0.622
ρ_d (M&F)	0.117	−0.166	0.387	0.177	−0.240	0.544	0.118	−0.176	0.387	0.173	−0.254	0.550
ρ_d (M&S_1)	0.376	0.150	0.573	0.446	0.223	0.644	0.385	0.147	0.587	0.503	0.257	0.709
ρ_d (M&S_2)	0.254	0.008	0.473	0.299	0.049	0.521	0.242	−0.019	0.476	0.281	−0.009	0.534
ρ_d (F&S_1)	0.096	−0.199	0.374	0.071	−0.321	0.436	0.089	−0.207	0.376	0.050	−0.364	0.443
ρ_d (F&S_2)	0.145	−0.143	0.417	0.163	−0.236	0.519	0.142	−0.143	0.411	0.151	−0.268	0.515
ρ_d (S_1&S_2)	0.262	−0.011	0.497	0.399	0.043	0.673	0.237	−0.038	0.483	0.321	−0.071	0.633
σ^2_{e1} (M▸F)							0.291	0.227	0.375			
σ^2_{e1} (F▸M)							0.219	0.167	0.283			
σ^2_{e1} (M▸S_1)							0.444	0.362	0.544			
σ^2_{e1} (S_1▸M)							0.348	0.282	0.427			
σ^2_{e1} (M▸S_2)							0.327	0.265	0.402			
σ^2_{e1} (S_2▸M)							0.387	0.314	0.473			
σ^2_{e1} (F▸S_1)							0.376	0.294	0.483			
σ^2_{e1} (S_1▸F)							0.321	0.250	0.409			
σ^2_{e1} (F▸S_2)							0.362	0.282	0.462			
σ^2_{e1} (S_2▸F)							0.290	0.227	0.369			
σ^2_{e1} (S_1▸S_2)							0.216	0.171	0.271			
σ^2_{e1} (S_2▸S_1)							0.353	0.282	0.438			
σ^2_{η} (M▸F)	0.217	0.202	0.234				0.211	0.195	0.229			
σ^2_{η} (F▸M)	0.218	0.202	0.234				0.221	0.205	0.238			
σ^2_{η} (M▸S_1)	0.360	0.338	0.383				0.355	0.332	0.380			
σ^2_{η} (S_1▸M)	0.294	0.276	0.313				0.293	0.274	0.313			
σ^2_{η} (M▸S_2)	0.300	0.282	0.319				0.301	0.282	0.320			
σ^2_{η} (S_2▸M)	0.319	0.301	0.339				0.316	0.296	0.338			
σ^2_{η} (F▸S_1)	0.290	0.269	0.313				0.285	0.262	0.309			
σ^2_{η} (S_1▸F)	0.283	0.262	0.306				0.283	0.262	0.307			
σ^2_{η} (F▸S_2)	0.294	0.273	0.317				0.290	0.268	0.314			
σ^2_{η} (S_2▸F)	0.240	0.223	0.259				0.238	0.220	0.257			
σ^2_{η} (S_1▸S_2)	0.269	0.250	0.290				0.279	0.258	0.303			
σ^2_{η} (S_2▸S_1)	0.298	0.276	0.321				0.296	0.272	0.321			
ρ_{e1} (M&F)							−0.078	−0.257	0.100	−0.139	−0.436	0.181

(cont.)

(cont.)

	Continuous SRM			Ordinal SRM			Continuous DSRM			Ordinal DSRM		
	Mean	Quantiles		Mean	Quantiles		Mean	Quantiles		Mean	Quantiles	
		2.5%	97.5%		2.5%	97.5%		2.5%	97.5%		2.5%	97.5%
ρ_{e1} (M&S$_1$)							0.033	−0.115	0.176	0.044	−0.119	0.203
ρ_{e1} (M&S$_2$)							0.221	0.082	0.356	0.289	0.110	0.453
ρ_{e1} (F&S$_1$)							0.319	0.156	0.469	0.398	0.199	0.580
ρ_{e1} (F&S$_2$)							0.208	0.039	0.370	0.241	0.016	0.447
ρ_{e1} (S$_1$&S$_2$)							0.098	−0.060	0.252	0.158	−0.086	0.386
ρ_η (M&F)	−0.026	−0.078	0.026	−0.041	−0.145	0.063	−0.018	−0.074	0.037	−0.021	−0.133	0.093
ρ_η (M&S$_1$)	0.067	0.022	0.112	0.080	0.025	0.136	0.073	0.027	0.121	0.089	0.029	0.149
ρ_η (M&S$_2$)	0.100	0.057	0.142	0.128	0.071	0.184	0.085	0.039	0.130	0.104	0.042	0.165
ρ_η (F&S$_1$)	0.159	0.107	0.211	0.213	0.138	0.283	0.139	0.083	0.196	0.184	0.104	0.263
ρ_η (F&S$_2$)	0.084	0.031	0.136	0.125	0.044	0.206	0.067	0.010	0.122	0.108	0.019	0.195
ρ_η (S$_1$&S$_2$)	0.188	0.137	0.239	0.301	0.222	0.376	0.206	0.150	0.259	0.326	0.241	0.407

Table S9: Approximate scales for parameters in the ordinal models as a multiple of those in the continuous models, based on estimated innovation variances

	Non-dynamic	Dynamic	
	Square	Linear	Square
M ▸ F	4.6	2.2	4.7
F ▸ M	4.6	2.1	4.5
M ▸ S1	2.8	1.7	2.8
S1 ▸ M	3.4	1.8	3.4
M ▸ S2	3.3	1.8	3.3
S2 ▸ M	3.1	1.8	3.2
F ▸ S1	3.4	1.9	3.5
S1 ▸ F	3.5	1.9	3.5
F ▸ S2	3.4	1.9	3.4
S2 ▸ F	4.2	2.0	4.2
S1 ▸ S2	3.7	1.9	3.6
S2 ▸ S1	3.4	1.8	3.4

Table S8 shows the results for models without dynamics, treating the response as continuous and treating it as ordinal, and models with full dynamics (i.e. both autoregressive effects and cross-lagged effects), again treating the response as continuous and treating it as ordinal. The results are not directly comparable between continuous and ordinal models, because the scale of the response is different in each. Nevertheless, there are a couple of ways in which we can get an approximate idea of how the scales correspond. One is by comparing the increase in the response for each model that would correspond to an increase of 1 category on the five point scale used to measure constructiveness. For the models treating the response as continuous, this would be an increase of 1. For the models treating the response as ordinal, it would be any increase in the underlying unobserved response y^* that takes it across exactly one of the thresholds. We can therefore regard a distance of 1 on the scale of y in the continuous models as approximately corresponding to the distance between each pair of thresholds in the ordinal models, i.e. to roughly 2 to 2.5, and can thus expect the autoregressive effects and cross-lagged effects to be approximately twice to two and a half times as big in the ordinal dynamic model as in the continuous dynamic model, and the variance parameters to be four to six and a half times as big in each ordinal model as in the corresponding continuous model.

The other approach is to use the innovations, regarding their standard deviation as equal but measured on different scales, since it is the fixing of the innovations that determines the scale of the ordinal models. For the ordinal models, all innovation variances and hence their standard deviations are of course 1. For the continuous model, the point estimates of the innovation variances range (across dyad types, and considering for the dynamic model only the innovations for snapshots after the first) from around 2 to around 3.5, implying standard deviations ranging from around 0.5 to 0.6, and thus that we should expect the autoregressive effects and cross-lagged effects to be between 1.7 and 2 times as big in the ordinal dynamic model as in the continuous dynamic model, and the variances to be 2.8 to 4 times as big in each ordinal model as in the corresponding continuous model. Table S9 shows for each dyad type (in the Linear column) how many times bigger we would expect the autoregressive effects and cross-lagged effects to be in the ordinal dynamic model than in the continuous dynamic model (based on the innovations for snapshots after the first), and (in the Square column) how many times bigger we would expect the variances to be in the ordinal model than in the continuous model for both the non-dynamic models (based on the innovations at all snapshots) and the dynamic models (again based on the innovations for snapshots after the first). We can see that for some dyad types, the (linear) scale factor lies between the size of the gap between the first two thresholds and the size of the gap between the second two thresholds (albeit for most types closer to the size of the first gap than the second), indicating that both approaches agree on the approximate conversion between the scale of the ordinal model and the continuous model, but for others the scale factor is somewhat smaller than the size of the first gap implying some minor disagreement between the two.

In addition, the fact that the continuous models parameter estimates are based on Pearson correlations while those of

the ordinal models are based on polychoric correlations means that we would expect larger estimates in each ordinal than in the corresponding continuous model of the autoregressive effects, cross-lagged effects, and actor-partner, dyad type, and innovation correlations even if the two models were on the same scale.

The autoregressive effects show almost the same pattern of significance in the ordinal dynamic model as in the continuous dynamic model, with all autoregressive effects being either significant in both models or significant in neither except for that for older children acting towards fathers, which is significant in the ordinal but not in the continuous model. Considering the point estimates of only those autoregressive effects which are significant in both models, each is rather less than twice as large in the ordinal model as it is in the continuous model, and the ratios of the point estimates in the ordinal model to those in the continuous model are also lower than the predicted ratios shown in Table S9, apart from that for older children acting towards younger children which matches the predicted value (to 1d.p.). Thus it seems that the ordinal model may find less persistence in an individual's constructiveness than the continuous model, although the credible intervals in both models are quite wide and so the differences may just be due to estimation variance.

The cross-lagged effects also show almost the same pattern of significance in both models, with the exception in this case being that the cross-lagged effects for older children acting towards mothers is just significant in the continuous model but not significant in the ordinal model. Only one cross-lagged effect is significant in both models, that for younger siblings acting towards older siblings, and its point estimate is 1.2 times as large in the ordinal model as in the continuous model, as against the 1.9 times predicted based on the innovation variance in the continuous model. Thus this effect too seems to be estimated as less strong in the ordinal model, although again the credible intervals are wide and we cannot definitely conclude any real difference.

The point estimate of the family variance is 6.5 times greater in the ordinal non-dynamic model than in the continuous non-dynamic model, or at the top end of what we might expect using the distance between thresholds as our basis for an approximation scale and rather bigger than what we might expect for any dyad type using the innovation variances. For the dynamic models, the point estimate is 15.5 times greater in the ordinal model than in the continuous model, i.e. considerably greater than we would expect on either basis. However, in both ordinal models the credible intervals are wide (and indeed for all four models they stretch down to zero or almost zero at the lower end), so while there is some suggestion that the family may be considered more important in the ordinal dynamic and perhaps also non-dynamic model, we once again cannot conclude there definitely is any real difference.

The point estimates of the mother actor variance are 4.5 times greater in each ordinal model than in the corresponding continuous model, and the point estimate of the father actor variance is also 4.5 times greater in the ordinal non-dynamic model than in the continuous non-dynamic model while for the dynamic models it is 4.3 times greater. This is at the lower end of what we might expect based on the thresholds and at the upper end of what we might expect based on the innovation variances. The point estimate of the younger child actor variance is 3.2 times greater in the ordinal non-dynamic model than in the continuous non-dynamic model and 3 times greater in the ordinal dynamic model than in the continuous dynamic model; and the point estimate of the older child actor variance is 3 times greater in the ordinal non-dynamic model than in the continuous non-dynamic model and 2.8 times greater in the ordinal dynamic model than in the continuous dynamic model. This is below what would be expected based on the thresholds, and also somewhat below what would be expected based on the innovations. Thus the mother's and father's influence as actors seems to be rated about equally in continuous and ordinal models, but the ordinal models may estimate the children's influence as less than the continuous models do. Once again, the credible intervals for the ordinal model are wide so we cannot conclude there is any real difference for the children.

The point estimates of the mother partner variance are 2.6 times greater in each ordinal model than in the corresponding continuous model, and the point estimates of the father partner variance are 2.2 times greater. The point estimate of the younger child partner variance is 3.4 times greater in the ordinal non-dynamic model than in the continuous non-dynamic model, and 3.2 times greater in the ordinal dynamic model than in the continuous dynamic model, while the point estimate of the older child partner variance is 2.9 times greater in the ordinal non-dynamic model than in the continuous non-dynamic model and 2.7 times greater in the ordinal dynamic model than in the continuous dynamic model. These are all lower than would be expected according to either approximate conversion, suggesting that the influence of each partner is estimated to be less important by the ordinal models than by

the continuous model – although once again the credible intervals for the ordinal model are wide and we cannot conclude this is definitely the case.

The point estimates of the dyad type variances are between 2.4 and 6.1 times bigger (depending on the dyad type) in the ordinal non-dynamic model than in the continuous non-dynamic model, and between 2.3 and 6.1 times bigger in the ordinal dynamic model than in the continuous dynamic model. The lower end of this range is smaller than we might expect going by the approximate scale conversion based on the thresholds, but the mid to upper portion is consistent with it. Going by the approximate scale conversion based on the innovation variances, in five dyad types the ratio of the ordinal point estimate to the continuous point estimate is quite a bit smaller than we would expect for both dynamic and non-dynamic models, in six dyad types it is quite a bit larger than we would expect, and in one dyad type it is close to what we would expect but slightly larger than we would expect in the non-dynamic models and slightly smaller than we would expect in the dynamic models. Again the credible intervals are wide, particularly for the ordinal models, so although there is some suggestion that the ordinal and continuous models may not agree on which dyad types the dyad type variance is relatively more important in, we cannot conclude there is any real difference.

To compare intercepts between the continuous and ordinal models, we use a different approach, as the correspondence between the two that we are interested in here relates to location rather than scale. We note that the assumption of a symmetric distribution with a mean of 0 for each kind of random effect (including the innovations) means that an intercept in the ordinal model exactly equal to the second threshold implies at the first snapshot an equal probability of the observed response being 3 or less and of its being 4 or more; and more generally this is also the case at any snapshot for a predicted value of y^* exactly equal to the second threshold. We can thus regard the estimate of the second threshold in the ordinal model as corresponding to a value of 3.5 in the continuous model, since that too, as an intercept for the first snapshot or as a predicted value more generally, implies an equal probability of the actual response being 3 or less or being 4 or more. Similarly, a predicted value in the ordinal model exactly equal to the first threshold implies an equal probability of the observed response being 2 or being 3 or more, corresponding to a value of 2.5 in the continuous model, for which this is also true; and a predicted value in the ordinal model exactly equal to the third threshold implies an equal probability of the observed response being 4 or less or being 5, corresponding to a value of 4.5 in the continuous model.

With this in mind, we observe that, for both dynamic and non-dynamic models, all the intercepts with point estimates between 2.5 and 3.5 in the continuous model have point estimates between the first and second thresholds in the corresponding ordinal model, and all the intercepts with point estimates between 3.5 and 4.5 in the continuous model have point estimates between the second and third thresholds in the corresponding ordinal model. Moreover, all four models agree on the ordering of the point estimates across dyad types.

Correlations are always on the same scale regardless of the model they appear in, but nevertheless are expected to be larger in each ordinal than in the corresponding continuous model since, as mentioned above, those in the continuous models are Pearson correlations while those in the ordinal models are polychoric correlations.

The actor-partner correlations for mothers and for fathers are non-significant in all models, and that for older children is significant in all models, but that for younger children is significant in the ordinal non-dynamic model and (just) in the continuous dynamic model but not in the continuous non-dynamic or ordinal dynamic models. As expected, the point estimate of the correlation for older children is larger in the ordinal models than in the continuous models (though the credible intervals are wide).

In the non-dynamic models, the dyad type correlations are non-significant in both models for mothers and fathers, fathers and younger children, and fathers and older children, and significant in both models for mothers and younger children and mothers and older children. The dyad type correlation for older and younger siblings is significant in the ordinal but not in the continuous model. The point estimates of the correlations for mothers and younger children and for mothers and older children are, as expected, larger in the ordinal model than in the continuous model (though again the confidence intervals are large). In the dynamic models, the pattern of significance is the same between the continuous and the ordinal model, with the correlation for mothers and younger children being significant in both models and the correlations for other dyad types being significant in neither, and again, as expected, the point

estimate of the correlation for mothers and younger children is larger in the ordinal than in the continuous model (although once again the credible intervals are large).

The pattern of significance for the innovation correlations is the same across all four models, with that for mothers and fathers not being significant in any model and those for other dyad types being significant in all four models. For all dyad types where the correlation is significant, the point estimate is larger in each ordinal model than in the corresponding continuous model (though the credible intervals are fairly wide).

Table S10: Proportions of total variance attributable to each component

	Continuous SRM			Ordinal SRM			Continuous DSRM			Ordinal DSRM		
	Mean	Quantiles		Mean	Quantiles		Mean	Quantiles		Mean	Quantiles	
		2.5%	97.5%		2.5%	97.5%		2.5%	97.5%		2.5%	97.5%
Family (M▶F)	0.006	0.001	0.016	0.009	0.000	0.038	0.006	0.001	0.018	0.021	0.000	0.073
Family (F▶M)	0.006	0.001	0.016	0.009	0.000	0.039	0.006	0.001	0.017	0.021	0.000	0.075
Family (M▶S_1)	0.004	0.001	0.011	0.007	0.000	0.031	0.004	0.001	0.012	0.017	0.000	0.060
Family (S_1▶M)	0.004	0.001	0.012	0.008	0.000	0.034	0.004	0.001	0.013	0.019	0.000	0.067
Family (M▶S_2)	0.004	0.001	0.012	0.007	0.000	0.032	0.004	0.001	0.013	0.018	0.000	0.063
Family (S_2▶M)	0.004	0.001	0.012	0.008	0.000	0.035	0.004	0.001	0.013	0.020	0.000	0.070
Family (F▶S_1)	0.004	0.001	0.013	0.008	0.000	0.034	0.005	0.001	0.014	0.019	0.000	0.066
Family (S_1▶F)	0.005	0.001	0.014	0.009	0.000	0.041	0.005	0.001	0.015	0.023	0.000	0.079
Family (F▶S_2)	0.004	0.001	0.013	0.008	0.000	0.036	0.005	0.001	0.014	0.020	0.000	0.070
Family (S_2▶F)	0.005	0.001	0.016	0.010	0.000	0.042	0.006	0.001	0.017	0.023	0.000	0.080
Family (S_1▶S_2)	0.005	0.001	0.014	0.009	0.000	0.041	0.005	0.001	0.015	0.023	0.000	0.079
Family (S_2▶S_1)	0.004	0.001	0.013	0.008	0.000	0.038	0.005	0.001	0.014	0.021	0.000	0.074
Actor (M▶F)	0.136	0.098	0.182	0.143	0.091	0.199	0.138	0.099	0.182	0.137	0.087	0.193
Actor (F▶M)	0.127	0.091	0.171	0.136	0.090	0.190	0.126	0.090	0.169	0.128	0.080	0.184
Actor (M▶S_1)	0.091	0.064	0.123	0.114	0.071	0.162	0.091	0.064	0.123	0.111	0.069	0.160
Actor (S_1▶M)	0.092	0.065	0.126	0.082	0.051	0.119	0.093	0.066	0.127	0.076	0.046	0.114
Actor (M▶S_2)	0.105	0.074	0.142	0.120	0.074	0.173	0.104	0.073	0.141	0.117	0.072	0.170
Actor (S_2▶M)	0.081	0.057	0.110	0.069	0.041	0.104	0.082	0.058	0.113	0.066	0.038	0.102
Actor (F▶S_1)	0.102	0.071	0.139	0.117	0.075	0.167	0.103	0.073	0.140	0.113	0.068	0.164
Actor (S_1▶F)	0.107	0.076	0.145	0.100	0.062	0.142	0.107	0.076	0.144	0.091	0.055	0.133
Actor (F▶S_2)	0.103	0.072	0.141	0.126	0.081	0.179	0.104	0.074	0.141	0.120	0.073	0.175
Actor (S_2▶F)	0.109	0.078	0.145	0.084	0.052	0.123	0.109	0.078	0.146	0.077	0.045	0.116
Actor (S_1▶S_2)	0.112	0.080	0.150	0.100	0.063	0.141	0.108	0.078	0.145	0.090	0.055	0.132
Actor (S_2▶S_1)	0.088	0.063	0.119	0.074	0.045	0.110	0.089	0.063	0.121	0.070	0.041	0.106
Partner (M▶F)	0.084	0.060	0.114	0.044	0.027	0.067	0.085	0.061	0.115	0.042	0.026	0.064
Partner (F▶M)	0.098	0.070	0.133	0.061	0.037	0.094	0.097	0.069	0.132	0.059	0.035	0.091
Partner (M▶S_1)	0.070	0.049	0.095	0.066	0.038	0.102	0.071	0.050	0.098	0.064	0.037	0.099
Partner (S_1▶M)	0.072	0.050	0.098	0.054	0.032	0.083	0.073	0.051	0.100	0.053	0.031	0.082
Partner (M▶S_2)	0.074	0.052	0.100	0.053	0.031	0.082	0.074	0.052	0.101	0.051	0.029	0.079

(cont.)

(cont.)

	Continuous SRM			Ordinal SRM			Continuous DSRM			Ordinal DSRM		
	Mean	Quantiles		Mean	Quantiles		Mean	Quantiles		Mean	Quantiles	
		2.5%	97.5%		2.5%	97.5%		2.5%	97.5%		2.5%	97.5%
Partner (S_2▶M)	0.071	0.050	0.097	0.055	0.032	0.085	0.072	0.051	0.100	0.055	0.032	0.086
Partner (F▶S_1)	0.084	0.060	0.115	0.073	0.042	0.112	0.086	0.060	0.117	0.071	0.040	0.109
Partner (S_1▶F)	0.072	0.051	0.098	0.047	0.029	0.072	0.071	0.051	0.097	0.046	0.028	0.069
Partner (F▶S_2)	0.078	0.055	0.105	0.059	0.035	0.091	0.079	0.056	0.107	0.057	0.033	0.088
Partner (S_2▶F)	0.081	0.058	0.111	0.049	0.030	0.074	0.081	0.058	0.111	0.046	0.029	0.070
Partner (S_1▶S_2)	0.085	0.061	0.114	0.068	0.041	0.103	0.083	0.059	0.113	0.065	0.038	0.099
Partner (S_2▶S_1)	0.083	0.059	0.112	0.081	0.047	0.123	0.084	0.059	0.114	0.079	0.046	0.121
Dyad type (M▶F)	0.158	0.109	0.217	0.115	0.063	0.181	0.160	0.110	0.219	0.108	0.059	0.172
Dyad type (F▶M)	0.148	0.101	0.206	0.093	0.051	0.149	0.144	0.098	0.202	0.086	0.046	0.140
Dyad type (M▶S_1)	0.156	0.110	0.209	0.265	0.193	0.340	0.153	0.106	0.206	0.250	0.178	0.325
Dyad type (S_1▶M)	0.221	0.165	0.283	0.247	0.180	0.318	0.208	0.150	0.272	0.219	0.151	0.293
Dyad type (M▶S_2)	0.165	0.115	0.221	0.242	0.170	0.317	0.159	0.110	0.216	0.225	0.152	0.304
Dyad type (S_2▶M)	0.189	0.137	0.246	0.242	0.175	0.313	0.175	0.122	0.235	0.204	0.134	0.280
Dyad type (F▶S_1)	0.149	0.100	0.208	0.198	0.123	0.282	0.149	0.098	0.210	0.181	0.106	0.265
Dyad type (S_1▶F)	0.132	0.089	0.186	0.102	0.056	0.163	0.128	0.086	0.180	0.089	0.049	0.146
Dyad type (F▶S_2)	0.138	0.093	0.194	0.160	0.092	0.240	0.135	0.090	0.191	0.142	0.076	0.221
Dyad type (S_2▶F)	0.143	0.099	0.198	0.093	0.053	0.146	0.142	0.098	0.196	0.087	0.049	0.139
Dyad type (S_1▶S_2)	0.120	0.082	0.166	0.080	0.045	0.126	0.115	0.078	0.160	0.072	0.040	0.116
Dyad type (S_2▶S_1)	0.160	0.110	0.217	0.164	0.100	0.235	0.152	0.103	0.208	0.138	0.078	0.210
Innovation (M▶F)	0.616	0.559	0.670	0.690	0.625	0.750	0.612	0.554	0.665	0.692	0.624	0.755
Innovation (F▶M)	0.621	0.565	0.674	0.701	0.641	0.756	0.626	0.570	0.678	0.706	0.643	0.763
Innovation (M▶S_1)	0.679	0.632	0.724	0.548	0.494	0.600	0.681	0.632	0.727	0.558	0.502	0.614
Innovation (S_1▶M)	0.611	0.560	0.660	0.610	0.554	0.664	0.622	0.568	0.673	0.632	0.571	0.691
Innovation (M▶S_2)	0.653	0.605	0.697	0.578	0.525	0.629	0.658	0.610	0.702	0.589	0.533	0.643
Innovation (S_2▶M)	0.655	0.606	0.700	0.626	0.571	0.677	0.666	0.614	0.714	0.655	0.594	0.712
Innovation (F▶S_1)	0.661	0.606	0.711	0.604	0.539	0.664	0.658	0.601	0.710	0.617	0.551	0.680
Innovation (S_1▶F)	0.684	0.633	0.731	0.742	0.688	0.790	0.690	0.639	0.736	0.752	0.691	0.805
Innovation (F▶S_2)	0.677	0.625	0.726	0.647	0.585	0.705	0.677	0.624	0.726	0.660	0.596	0.719
Innovation (S_2▶F)	0.661	0.608	0.711	0.765	0.709	0.814	0.662	0.608	0.712	0.766	0.705	0.818
Innovation (S_1▶S_2)	0.678	0.628	0.725	0.743	0.691	0.791	0.689	0.639	0.735	0.751	0.693	0.803
Innovation (S_2▶S_1)	0.665	0.612	0.715	0.672	0.612	0.728	0.671	0.617	0.721	0.692	0.627	0.753

Table S11: Proportions of total dyad type variance attributable to each component

	Continuous SRM			Ordinal SRM			Continuous DSRM			Ordinal DSRM		
	Mean	Quantiles		Mean	Quantiles		Mean	Quantiles		Mean	Quantiles	
		2.5%	97.5%		2.5%	97.5%		2.5%	97.5%		2.5%	97.5%
Family (M▸F)	0.014	0.003	0.042	0.027	0.001	0.119	0.015	0.003	0.046	0.066	0.001	0.219
Family (F▸M)	0.015	0.003	0.043	0.029	0.001	0.129	0.015	0.003	0.046	0.071	0.001	0.239
Family (M▸S_1)	0.012	0.002	0.034	0.015	0.000	0.068	0.012	0.002	0.037	0.038	0.001	0.132
Family (S_1▸M)	0.010	0.002	0.031	0.020	0.001	0.087	0.011	0.002	0.035	0.052	0.001	0.175
Family (M▸S_2)	0.012	0.002	0.036	0.017	0.001	0.076	0.013	0.002	0.039	0.043	0.001	0.148
Family (S_2▸M)	0.012	0.002	0.034	0.021	0.001	0.094	0.013	0.002	0.039	0.057	0.001	0.193
Family (F▸S_1)	0.013	0.002	0.039	0.019	0.001	0.086	0.014	0.002	0.042	0.049	0.001	0.170
Family (S_1▸F)	0.015	0.003	0.044	0.035	0.001	0.155	0.016	0.003	0.047	0.088	0.002	0.284
Family (F▸S_2)	0.014	0.003	0.041	0.023	0.001	0.102	0.015	0.003	0.045	0.058	0.001	0.198
Family (S_2▸F)	0.016	0.003	0.047	0.040	0.001	0.170	0.017	0.003	0.051	0.095	0.002	0.302
Family (S_1▸S_2)	0.015	0.003	0.045	0.036	0.001	0.159	0.016	0.003	0.048	0.088	0.002	0.287
Family (S_2▸S_1)	0.013	0.002	0.038	0.025	0.001	0.114	0.014	0.002	0.042	0.067	0.001	0.226
Actor (M▸F)	0.356	0.257	0.461	0.461	0.304	0.614	0.356	0.258	0.458	0.448	0.288	0.609
Actor (F▸M)	0.336	0.242	0.439	0.455	0.311	0.599	0.339	0.244	0.441	0.437	0.278	0.592
Actor (M▸S_1)	0.284	0.199	0.379	0.252	0.155	0.362	0.287	0.199	0.383	0.252	0.155	0.364
Actor (S_1▸M)	0.238	0.163	0.326	0.211	0.127	0.312	0.248	0.170	0.340	0.209	0.120	0.320
Actor (M▸S_2)	0.302	0.211	0.406	0.286	0.174	0.412	0.306	0.213	0.410	0.286	0.172	0.418
Actor (S_2▸M)	0.236	0.162	0.325	0.186	0.106	0.286	0.247	0.169	0.341	0.193	0.107	0.308
Actor (F▸S_1)	0.300	0.210	0.403	0.298	0.185	0.425	0.302	0.211	0.405	0.296	0.175	0.433
Actor (S_1▸F)	0.341	0.243	0.449	0.388	0.239	0.545	0.345	0.246	0.453	0.368	0.217	0.533
Actor (F▸S_2)	0.319	0.224	0.425	0.358	0.224	0.510	0.323	0.229	0.429	0.356	0.209	0.516
Actor (S_2▸F)	0.321	0.232	0.419	0.359	0.222	0.507	0.323	0.232	0.422	0.332	0.192	0.492
Actor (S_1▸S_2)	0.348	0.256	0.448	0.389	0.255	0.526	0.348	0.255	0.448	0.363	0.222	0.514
Actor (S_2▸S_1)	0.265	0.185	0.356	0.227	0.133	0.344	0.270	0.190	0.364	0.229	0.128	0.355
Partner (M▸F)	0.220	0.155	0.298	0.142	0.084	0.221	0.219	0.155	0.297	0.138	0.080	0.216
Partner (F▸M)	0.260	0.185	0.348	0.206	0.120	0.314	0.261	0.184	0.348	0.202	0.115	0.314
Partner (M▸S_1)	0.219	0.152	0.300	0.147	0.083	0.231	0.224	0.154	0.307	0.145	0.081	0.229
Partner (S_1▸M)	0.185	0.128	0.255	0.138	0.079	0.216	0.193	0.132	0.268	0.145	0.081	0.231

(cont.)

(cont.)

	Continuous SRM			Ordinal SRM			Continuous DSRM			Ordinal DSRM		
	Mean	Quantiles		Mean	Quantiles		Mean	Quantiles		Mean	Quantiles	
		2.5%	97.5%		2.5%	97.5%		2.5%	97.5%		2.5%	97.5%
Partner (M▸S_2)	0.212	0.147	0.290	0.126	0.072	0.199	0.217	0.150	0.298	0.124	0.070	0.199
Partner (S_2▸M)	0.206	0.142	0.283	0.148	0.084	0.233	0.217	0.149	0.301	0.160	0.089	0.258
Partner (F▸S_1)	0.250	0.173	0.340	0.186	0.103	0.292	0.252	0.174	0.343	0.186	0.101	0.297
Partner (S_1▸F)	0.227	0.158	0.309	0.184	0.109	0.280	0.229	0.161	0.311	0.186	0.109	0.287
Partner (F▸S_2)	0.241	0.168	0.327	0.169	0.095	0.264	0.244	0.171	0.330	0.169	0.094	0.269
Partner (S_2▸F)	0.241	0.169	0.327	0.208	0.123	0.319	0.241	0.170	0.327	0.201	0.118	0.310
Partner (S_1▸S_2)	0.265	0.189	0.351	0.265	0.159	0.391	0.268	0.192	0.354	0.261	0.152	0.391
Partner (S_2▸S_1)	0.248	0.174	0.334	0.249	0.143	0.376	0.257	0.179	0.347	0.259	0.146	0.395
Dyad type (M▸F)	0.410	0.300	0.524	0.369	0.220	0.533	0.410	0.300	0.526	0.349	0.204	0.511
Dyad type (F▸M)	0.389	0.284	0.505	0.310	0.179	0.460	0.385	0.279	0.498	0.290	0.164	0.442
Dyad type (M▸S_1)	0.485	0.371	0.597	0.585	0.453	0.707	0.477	0.360	0.593	0.564	0.426	0.691
Dyad type (S_1▸M)	0.567	0.459	0.667	0.631	0.507	0.743	0.548	0.435	0.655	0.595	0.449	0.724
Dyad type (M▸S_2)	0.473	0.352	0.589	0.571	0.428	0.704	0.464	0.343	0.584	0.546	0.391	0.688
Dyad type (S_2▸M)	0.546	0.433	0.650	0.646	0.510	0.761	0.523	0.403	0.634	0.590	0.428	0.730
Dyad type (F▸S_1)	0.436	0.318	0.556	0.497	0.338	0.645	0.432	0.315	0.553	0.469	0.304	0.626
Dyad type (S_1▸F)	0.417	0.301	0.538	0.393	0.236	0.565	0.410	0.297	0.529	0.358	0.207	0.532
Dyad type (F▸S_2)	0.426	0.308	0.547	0.450	0.283	0.613	0.418	0.302	0.541	0.416	0.244	0.588
Dyad type (S_2▸F)	0.421	0.313	0.535	0.393	0.245	0.551	0.419	0.310	0.532	0.372	0.223	0.537
Dyad type (S_1▸S_2)	0.371	0.269	0.481	0.310	0.185	0.457	0.368	0.268	0.479	0.287	0.165	0.436
Dyad type (S_2▸S_1)	0.474	0.360	0.588	0.498	0.337	0.647	0.459	0.343	0.572	0.446	0.278	0.610

Table S10 shows the variance partitioning coefficients for the same four models, and Table S11 shows the proportion of the total dyad type variance (i.e. the sum of all variance components other than the innovation variance) accounted for by each component.

The family accounts for a somewhat larger proportion of the total variance and of the total dyad type variance in the ordinal than in the continuous models, going by the point estimates. The family is both the smallest component of the the VPC and the smallest component of total dyad type variance for all dyad types in all models, in most cases by some way (although the family components are not all that far behind the partner components for some dyad types in the ordinal dynamic model).

Going by point estimates, for some dyad types (specifically, all those where a child is the actor, plus mothers acting towards fathers in the dynamic models), the actor component of the VPC is larger in each of the continuous models than in the corresponding ordinal model, while for others (specifically, all those where a parent is the actor, other than mothers acting towards fathers in the dynamic models), it is smaller in each of the continuous models than in the corresponding ordinal models. Similarly, for some dyad types, the actor variance accounts for a greater proportion of the total dyad type variance in each of the continuous models than in the corresponding ordinal model, while for others it accounts for a smaller proportion. (However, in some cases the difference in point estimates between the continuous model and the corresponding ordinal model is very small.)

The point estimates of the proportions of the total dyad type variances accounted for by the partner variances are larger in each continuous model than in the corresponding ordinal model for every dyad type but two (younger siblings acting towards older siblings, for which this holds in the dynamic models but the proportions for the non-dynamic models are equal to 3d.p., and older siblings acting towards younger siblings, for which the proportions are slightly larger in both the dynamic and non-dynamic ordinal models), and the same is true for every dyad type for the partner components of the VPCs. For all models, for every dyad type with the exception of older siblings acting towards younger siblings in the ordinal models, the point estimate of both the VPC and the proportion of the total dyad type variance accounted for is greater for the actor variance than for the partner variance.

For all dyad types but one, the point estimate of the dyad type component of the VPC is either larger in the continuous model than in the corresponding ordinal model for both the dynamic than the non-dynamic models, or smaller in the continuous model in both cases. This is also the case for the proportions of the total dyad-type variance for all but two dyad types. Going by point estimates, the dyad type variance forms the largest component of the total dyad type variance, and the second largest component of the VPC, for all dyad types in the continuous models, but while this is also the case for some dyad types in the ordinal model, for others the actor variance accounts for a larger proportion than the dyad type variance.

For all but one dyad type, the point estimate of the innovation component of the VPC is either larger in each continuous model than in the corresponding ordinal model or smaller in each continuous model than in the corresponding ordinal model. Going by point estimates, the innovation forms the largest component of the VPC for all dyad types in all models, by some way; it accounts for more than 50% of the variance for every dyad type in every model, and for some dyad types in some models it accounts for as much as 75%.

Again, for all components, the credible intervals are wide, so the differences discussed here may not be real.

S5.2 Adding dynamics to the Social Relations Model

In this section, we present the results of progressively adding dynamics to the social relations model – first autoregressive effects and then also cross-lagged effects – alongside the orignal social relations model without dynamics, so we can see how this changes the results. Table S12 presents parameter estimates, Table S13 presents the variance partitioning coefficients, and Table S14 presents the proportions of the total dyad type variance (i.e. of the sum of all variance components other than the innovation) accounted for by each component.

Table S12: Results of Ordinal SRM, Ordinal DSRM (no crosslags), and Ordinal DSRM

	Ordinal SRM			Ordinal DSRM (no crosslags)			Ordinal DSRM		
	Mean	Quantiles		Mean	Quantiles		Mean	Quantiles	
		2.5%	97.5%		2.5%	97.5%		2.5%	97.5%
τ_1	0.000	0.000	0.000	0.000	0.000	0.000	0.000	0.000	0.000
τ_2	1.808	1.775	1.840	1.794	1.764	1.826	1.791	1.759	1.812
τ_3	4.429	4.376	4.481	4.398	4.340	4.454	4.386	4.322	4.443
μ (M▸F)	2.266	2.138	2.395	2.249	2.119	2.379	2.244	2.115	2.372
μ (F▸M)	2.032	1.906	2.161	2.017	1.887	2.149	2.015	1.886	2.146
μ (M▸S_1)	2.621	2.483	2.757	2.601	2.464	2.739	2.596	2.460	2.735
μ (S_1▸M)	1.151	1.029	1.273	1.141	1.019	1.263	1.139	1.017	1.260
μ (M▸S_2)	2.616	2.488	2.746	2.594	2.464	2.724	2.589	2.460	2.719
μ (S_2▸M)	1.531	1.413	1.650	1.519	1.401	1.635	1.516	1.400	1.632
μ (F▸S_1)	2.803	2.653	2.953	2.779	2.628	2.930	2.774	2.625	2.923
μ (S_1▸F)	1.480	1.363	1.595	1.473	1.355	1.590	1.471	1.352	1.590
μ (F▸S_2)	2.391	2.254	2.527	2.373	2.237	2.510	2.369	2.234	2.505
μ (S_2▸F)	1.548	1.439	1.659	1.533	1.422	1.645	1.531	1.420	1.643
μ (S_1▸S_2)	1.421	1.316	1.526	1.417	1.308	1.526	1.416	1.307	1.525
μ (S_2▸S_1)	1.658	1.540	1.778	1.651	1.530	1.771	1.648	1.529	1.766
ϕ_1 (M▸F)				0.070	−0.023	0.164	0.068	−0.025	0.162
ϕ_1 (F▸M)				0.202	0.110	0.294	0.202	0.112	0.293
ϕ_1 (M▸S_1)				0.091	0.026	0.157	0.095	0.029	0.161
ϕ_1 (S_1▸M)				0.227	0.154	0.300	0.224	0.150	0.297
ϕ_1 (M▸S_2)				0.108	0.042	0.175	0.102	0.033	0.171
ϕ_1 (S_2▸M)				0.242	0.173	0.311	0.237	0.168	0.307
ϕ_1 (F▸S_1)				0.089	0.003	0.174	0.085	−0.006	0.175
ϕ_1 (S_1▸F)				0.207	0.124	0.289	0.208	0.121	0.296
ϕ_1 (F▸S_2)				0.105	0.021	0.190	0.108	0.023	0.192
ϕ_1 (S_2▸F)				0.122	0.035	0.211	0.120	0.030	0.209
ϕ_1 (S_1▸S_2)				0.170	0.086	0.253	0.143	0.051	0.233
ϕ_1 (S_2▸S_1)				0.143	0.054	0.233	0.164	0.067	0.262
ϕ_2 (M▸F)							−0.024	−0.113	0.066
ϕ_2 (F▸M)							0.007	−0.078	0.092
ϕ_2 (M▸S_1)							−0.054	−0.135	0.027
ϕ_2 (S_1▸M)							−0.009	−0.065	0.046
ϕ_2 (M▸S_2)							0.056	−0.014	0.127
ϕ_2 (S_2▸M)							0.045	−0.015	0.106
ϕ_2 (F▸S_1)							0.016	−0.080	0.114
ϕ_2 (S_1▸F)							0.001	−0.073	0.075
ϕ_2 (F▸S_2)							−0.016	−0.115	0.080
ϕ_2 (S_2▸F)							0.002	−0.068	0.073
ϕ_2 (S_1▸S_2)							0.099	0.015	0.180
ϕ_2 (S_2▸S_1)							0.028	−0.067	0.123
σ_f^2	0.013	0.000	0.057	0.027	0.001	0.102	0.031	0.001	0.111
σ_a^2 (M)	0.208	0.127	0.305	0.202	0.123	0.298	0.200	0.121	0.294
σ_a^2 (F)	0.195	0.123	0.286	0.187	0.115	0.278	0.183	0.110	0.273
σ_a^2 (S_1)	0.135	0.083	0.197	0.122	0.073	0.184	0.121	0.072	0.182
σ_a^2 (S_2)	0.111	0.066	0.167	0.103	0.060	0.160	0.101	0.059	0.157
σ_p^2 (M)	0.088	0.052	0.137	0.085	0.049	0.134	0.084	0.049	0.132
σ_p^2 (F)	0.064	0.039	0.098	0.061	0.037	0.095	0.061	0.037	0.094

(cont.)

(cont.)

	Ordinal SRM			Ordinal DSRM (no crosslags)			Ordinal DSRM		
	Mean	Quantiles		Mean	Quantiles		Mean	Quantiles	
		2.5%	97.5%		2.5%	97.5%		2.5%	97.5%
σ_p^2 (S_1)	0.121	0.070	0.188	0.116	0.067	0.181	0.115	0.066	0.180
σ_p^2 (S_2)	0.092	0.054	0.142	0.088	0.050	0.137	0.087	0.050	0.136
σ_d^2 (M▸F)	0.168	0.088	0.281	0.159	0.081	0.268	0.157	0.081	0.266
σ_d^2 (F▸M)	0.134	0.070	0.225	0.122	0.063	0.209	0.122	0.063	0.210
σ_d^2 (M▸S_1)	0.488	0.329	0.674	0.455	0.301	0.638	0.451	0.300	0.630
σ_d^2 (S_1▸M)	0.408	0.277	0.568	0.350	0.220	0.504	0.350	0.223	0.504
σ_d^2 (M▸S_2)	0.421	0.278	0.591	0.393	0.248	0.563	0.386	0.243	0.557
σ_d^2 (S_2▸M)	0.390	0.263	0.542	0.316	0.193	0.463	0.315	0.193	0.463
σ_d^2 (F▸S_1)	0.332	0.190	0.513	0.298	0.162	0.474	0.296	0.161	0.472
σ_d^2 (S_1▸F)	0.139	0.073	0.233	0.120	0.062	0.204	0.119	0.063	0.204
σ_d^2 (F▸S_2)	0.249	0.134	0.400	0.219	0.112	0.361	0.218	0.110	0.362
σ_d^2 (S_2▸F)	0.122	0.067	0.202	0.115	0.061	0.191	0.115	0.062	0.191
σ_d^2 (S_1▸S_2)	0.108	0.059	0.178	0.100	0.053	0.167	0.096	0.052	0.161
σ_d^2 (S_2▸S_1)	0.247	0.141	0.377	0.210	0.112	0.336	0.201	0.106	0.327
ρ_{ap} (M)	0.195	−0.149	0.503	0.174	−0.186	0.490	0.171	−0.188	0.485
ρ_{ap} (F)	0.075	−0.265	0.400	0.049	−0.294	0.382	0.041	−0.301	0.374
ρ_{ap} (S_1)	0.326	0.009	0.589	0.312	−0.018	0.585	0.302	−0.039	0.580
ρ_{ap} (S_2)	0.407	0.105	0.646	0.369	0.038	0.626	0.360	0.025	0.622
ρ_d (M&F)	0.177	−0.240	0.544	0.171	−0.251	0.538	0.173	−0.254	0.550
ρ_d (M&S_1)	0.446	0.223	0.644	0.482	0.239	0.691	0.503	0.257	0.709
ρ_d (M&S_2)	0.299	0.049	0.521	0.326	0.046	0.572	0.281	−0.009	0.534
ρ_d (F&S_1)	0.071	−0.321	0.436	0.067	−0.345	0.457	0.050	−0.364	0.443
ρ_d (F&S_2)	0.163	−0.236	0.519	0.147	−0.269	0.517	0.151	−0.268	0.515
ρ_d (S_1&S_2)	0.399	0.043	0.673	0.367	−0.015	0.663	0.321	−0.071	0.633
ρ_{e1} (M&F)				−0.137	−0.432	0.180	−0.139	−0.436	0.181
ρ_{e1} (M&S_1)				0.037	−0.123	0.195	0.044	−0.119	0.203
ρ_{e1} (M&S_2)				0.298	0.126	0.461	0.289	0.110	0.453
ρ_{e1} (F&S_1)				0.396	0.197	0.577	0.398	0.199	0.580
ρ_{e1} (F&S_2)				0.240	0.020	0.445	0.241	0.016	0.447
ρ_{e1} (S_1&S_2)				0.147	−0.094	0.373	0.158	−0.086	0.386
ρ_η (M&F)	−0.041	−0.145	0.063	−0.019	−0.133	0.097	−0.021	−0.133	0.093
ρ_η (M&S_1)	0.080	0.025	0.136	0.091	0.031	0.151	0.089	0.029	0.149
ρ_η (M&S_2)	0.128	0.071	0.184	0.101	0.041	0.162	0.104	0.042	0.165
ρ_η (F&S_1)	0.213	0.138	0.283	0.183	0.103	0.260	0.184	0.104	0.263
ρ_η (F&S_2)	0.125	0.044	0.206	0.108	0.021	0.194	0.108	0.019	0.195
ρ_η (S_1&S_2)	0.301	0.222	0.376	0.321	0.236	0.401	0.326	0.241	0.407

For parameters common to all three models, in almost all cases, the point estimates for the model with autoregressive effects but without cross-lagged effects are closer to the point estimates for the model with full dynamics than the model with no dynamics (in some cases only slightly closer and in others a lot closer). The exceptions are the fixed intercept for fathers acting towards younger children (although this may be an artifact of rounding to 3d.p., since there is only 0.001 in it) and three of the dyadic reciprocities (those for mothers and older children, fathers and younger children, and older and younger siblings). The point estimates for the model with only autoregressive effects mostly lie between those for the model with no dynamics and those for the model with full dynamics, but not in all cases.

The largest difference in point estimates between the model with full dynamics and the model with no dynamics is 0.078, the amount by which the dyadic reciprocity for older and younger siblings is smaller in the model with full dynamics. Only three further parameters have differences in point estimates between these models of greater than 0.05: the dyad-type variance for older children acting towards mothers (0.075 smaller in the model with full dynamics), the dyad-type variance for younger children acting towards mothers (0.058 smaller in the model with full dynamics), and the dyadic reciprocity for mothers and younger children (0.057 larger in the model with full dynamics). Thirty-six parameters have differences in point estimates of at least 0.01 but less than 0.05. Eight have differences of at least 0.005 but less than 0.01. The remaining three parameters have differences of less than 0.005, with the smallest being 0.003, the amount by which the partner variance for fathers is smaller in the model with full dynamics.

The largest difference for the thresholds is 0.043, the amount by which τ_3 is lower in the model with full dynamics; and the smallest is 0.017, the amount by which τ_2 is lower in the model with full dynamics. The largest difference for the fixed intercepts is 0.028, the amount by which the intercept for fathers acting towards younger children is smaller in the model with full dynamics; and the smallest difference is 0.005, the amount by which the intercept for younger siblings acting towards older siblings is smaller in the model with full dynamics. The estimate of the family variance is 0.018 larger in the model with full dynamics. The largest difference for the actor variances is 0.014, the amount by which the actor variance for younger children is smaller in the model with full dynamics; and the smallest difference is 0.009, the amount by which the actor variance for mothers is smaller in the model with full dynamics. The largest difference for the partner variances is 0.007, the amount by which the partner variance for younger children is smaller in the model with full dynamics; and the smallest difference is 0.003, the amount by which the partner variance for fathers is smaller in the model with full dynamics. The largest difference for the dyad-type variances is 0.075, the amount by which the dyad-type variance for older children acting towards mothers is smaller in the model with full dynamics; and the smallest difference is 0.008, the amount by which the dyad-type variance for older children acting towards fathers is smaller in the model with full dynamics. The largest difference for the generalised reciprocities is 0.047, the amount by which the generalised reciprocity for older children is smaller in the model with full dynamics; and the smallest difference is 0.024, the amount by which the generalised reciprocity for younger children is smaller in the model with full dynamics). The largest difference for the dyadic reciprocities is 0.078, the amount by which the dyadic reciprocity for older and younger siblings is smaller in the model with full dynamics; and the smallest difference is 0.004, the amount by which the dyadic reciprocity for mothers and fathers is smaller in the model with full dynamics. The largest difference for the correlations between the innovations for dyads involving the same two individuals at the same snapshot is 0.029, the amount by which this correlation for fathers and younger children is smaller in the model with full dynamics; and the smallest difference is 0.009, the amount by which this correlation for mothers and younger children is larger in the model with full dynamics.

In terms of the differences as a percentage of the parameter estimate for the model with full dynamics, the largest is a difference of 100%, for the correlation between the innovations for the two dyad types involving mothers and fathers (which is larger in the model with full dynamics). There are two other differences which are greater than 50%: that for the generalised reciprocity for fathers (82% higher in the model with no dynamics), and that for the family variance (59% lower in the model with no dynamics). Seventeen parameters have differences of at least 10% but less than 50%; thirteen have differences of at least 5% but less than 10%; eight have differences of at least 1% but less than 5%, and the remaining ten parameters have differences of less than 1%.

The differences for the thresholds are both less than 1%. The largest difference for the fixed intercepts is for older children acting towards fathers (1% higher in the model with no dynamics) and the smallest difference is for younger siblings acting toward older siblings (higher by less than 1% in the model with no dynamics). The largest difference for the actor variances is for younger children (12% higher in the model with no dynamics) and the smallest difference is for mothers (4% higher in the model with no dynamics). The largest difference for the partner variances is for older children (6% higher in the model with no dynamics) and the smallest difference is for fathers (5% higher in the model with no dynamics). The largest difference for the dyad-type variances is for older children acting towards mothers (24% higher in the model with no dynamics) and the smallest difference is for mothers acting towards fathers (7% higher in the model with no dynamics). The largest difference for the generalised reciprocities is for fathers (82% higher in the model with no dynamics) and the smallest difference is for younger children (8% higher in the model with no dynamics). The largest difference for the dyadic reciprocities is for fathers and younger children

(44% higher in the model with no dynamics) and the smallest difference is for mothers and fathers (2% higher in the model with no dynamics). The largest difference for the correlations between innovations for dyads involving the same two individuals is for mothers and fathers (100% lower in the model with no dynamics) and the smallest difference is for older and younger siblings (8% lower in the model with no dynamics).

For the model with full dynamics and the model with autoregressive effects but no cross-lagged effects, the differences in point estimates are all less than 0.05. The largest is 0.045, which is the amount by which the dyadic reciprocities for both older and younger siblings and for mothers and older children are smaller in the model with full dynamics. A further seven parameters have differences of less than 0.05 but at least 0.01. Thirteen have differences of less than 0.01 but at least 0.005. The remaining forty-seven have differences of less than 0.005, including six which have differences of less than 0.0005.

The largest difference for the thresholds is 0.012, the amount by which τ_3 is smaller in the model with full dynamics; and the smallest is 0.004, the amount by which τ_2 is smaller in the model with full dynamics. The largest difference for the fixed intercepts is 0.005, the amount by which the intercept for fathers acting towards younger children is smaller in the model with full dynamics; and the smallest is 0.001, the amount by which the intercept for younger siblings acting towards older siblings is smaller in the model with full dynamics. The largest difference for the autoregressive effects is 0.027, the amount by which the autoregressive effect for younger siblings acting towards older siblings is smaller in the model with full dynamics; and the smallest is 0.000 (to the precision we calculate it), the amount by which the autoregressive effect for fathers acting towards mothers is larger in the model with full dynamics. The estimate of the family variance is 0.004 larger in the model with full dynamics. The largest difference for the actor variances is 0.004, the amount by which the actor variance for fathers is smaller in the model with full dynamics; and the smallest is 0.002, the amount by which the actor variance for younger children is smaller in the model with full dynamics. The partner variances are all 0.001 smaller in the model with full dynamics. The largest difference for the dyad-type variances is 0.009, the amount by which the dyad-type variance for older siblings acting towards younger siblings is smaller in the model with full dynamics; and the smallest is 0.000 (to the precision we calculate it), the amount by which the dyad-type variances for fathers acting towards mothers, younger children acting towards mothers, and older children acting towards fathers are all larger in the model with full dynamics, and also the amount by which the dyad-type variance for younger children acting towards fathers is smaller in the model with full dynamics. The largest difference for the generalised reciprocities is 0.010, the amount by which the generalised reciprocity for younger children is smaller in the model with full dynamics; and the smallest is 0.003, the amount by which the generalised reciprocity for mothers is smaller in the model with full dynamics. The largest difference for the dyadic reciprocities is 0.045, as mentioned above; and the smallest is 0.003, the amount by which the dyadic reciprocity for mothers and fathers is larger in the model with full dynamics. The largest difference for the correlations between the innovations at the first snapshot for dyad types that involve the same two family roles is 0.011, the amount by which the correlation for younger and older siblings is larger in the model with full dynamics; and the smallest is 0.000 (to the precision we calculate it), the amount by which the correlation for fathers and older siblings is larger in the model with full dynamics. The largest difference between this correlation for innovations at subsequent snapshots is 0.006, the amount by which the correlation for older and younger siblings is larger in the model with full dynamics; and the smallest is 0.001, the amount by which the correlation for fathers and older children is smaller in the model with full dynamics.

In terms of the differences as a percentage of the parameter estimate for the model with full dynamics, the largest difference is 36%, for the dyadic reciprocity for fathers and younger children (which is smaller in the model with full dynamics). Seven further parameters have percentage differences larger than 10%; three have percentage differences of at least 5% but less than 10%; twenty-eight have differences of at least 1% but less than 5%; and the remaining thirty have differences of less than 1%, with the smallest being 0.033, for the dyad-type variance for younger children acting towards mothers (which is larger in the model with full dynamics).

The differences for the thresholds and the fixed intercepts (which are all smaller in the model with full dynamics) are all less than 1%. The largest difference for the autoregressive effects is for younger children acting towards older children (19% larger in the model with autoregressive effects only) and the smallest is for fathers acting towards mothers (less than 1% smaller in the model with autoregressive effects only). The family variance is 13% smaller in the model with autoregressive effects only. The actor variances are either 1% (mothers and younger children) or 2$

(fathers and older children) larger in the model with autoregressive effects only. The partner variances are all 1% larger in the model with autoregressive effects only. The largest difference for the dyad-type variances is for older siblings acting towards younger siblings (4% larger in the model with autoregressive effects only), and the smallest is for younger siblings acting towards mothers (less than 1% smaller in the model with autoregressive effects only). The largest difference for the generalised reciprocities is for fathers (18% larger in the model with autoregressive effects only), and the smallest is for mothers (2% larger in the model with autoregressive effects only). The largest difference for the dyadic reciprocities is for fathers and younger children (as mentioned above), and the smallest is for mothers and fathers (2% smaller in the model with autoregressive effects only). The largest difference in the correlations between the innovations at the first snapshot for dyad types involving the same two family roles is for mothers and younger children (17% smaller in the model with autoregressive effects only), and the smallest is for fathers and older children (less than 1% smaller in the model with autoregressive effects only). The largest difference between these correlations for subsequent snapshots is for mothers and fathers (9% larger in the model with autoregressive effects only), and the smallest is for fathers and older children (1% larger in the model with autoregressive effects only).

For the model with autoregressive effects only and the model with no dynamics, the largest difference in point estimates is 0.074, the amount by which the dyad-type variance for older children acting towards mothers is larger in the model with no dynamics. Just one other parameter also has a difference of greater than 0.05, the dyad-type variance for younger children acting towards mothers (0.058 larger in the model with no dynamics). Thirty-five parameters have differences of less than 0.05 but at least 0.01; nine have differences of less than 0.01 but at least 0.005; and the remaining five have differences of less than 0.005, with the smallest being 0.002, the amount by which the partner variance for fathers is larger in the model with no dynamics.

The largest difference for the thresholds is 0.031, the amount by which τ_3 is higher in the model with no dynamics; and the smallest is 0.015, the amount by which τ_2 is higher in the model with no dynamics. The largest difference for the fixed intercepts is 0.023, the amount by which the intercept for fathers acting towards younger children is larger in the model with no dynamics; and the smallest is 0.004, the amount by which the intercept for younger siblings acting towards older siblings is larger in the model with no dynamics. The family variance is 0.014 smaller in the model with no dynamics. The largest difference for the actor variances is 0.012, the amount by which the actor variance for younger children is larger in the model with no dynamics; and the smallest is 0.006, the amount by which the actor variance for mothers is larger in the model with no dynamics. The largest difference for the partner variances is 0.005, the amount by which the partner variance for younger children is larger in the model with no dynamics; and the smallest is 0.002, the amount by which the partner variance for fathers is larger the model with no dynamics. The largest difference for the dyad-type variances is 0.074, as already mentioned; and the smallest is 0.008, the amount by which the dyad-type variance for older children acting towards fathers is larger in the model with no dynamics. The largest difference for the generalised reciprocities is 0.038, the amount by which the generalised reciprocity for older children is larger in the model with no dynamics; and the smallest is 0.014, the amount by which the generalised reciprocity for younger children is larger in the model with no dynamics. The largest difference for the dyadic reciprocities is 0.036, the amount by which the dyadic reciprocity for mothers and younger children is smaller in the model with no dynamics; and the smallest is 0.004, the amount by which the dyadic reciprocity for fathers and younger children is larger in the model with no dynamics. The largest difference for the correlations between the innovations for dyad types involving the same two family roles is 0.030, the amount by which this correlation for fathers and younger children is larger in the model with no dynamics; and the smallest is 0.010, the amount by which this correlation for mothers and younger children is smaller in the model with no dynamics.

In terms of the differences as a percentage of the estimate for the model with autoregressive effects only, the largest is 119%, for the correlation between the innovations at the same snapshot for dyad types involving mothers and fathers (which is smaller in the model with no dynamics). Only two other differences are greater than 50%: that for the generalised reciprocity for fathers (55% larger in the model with no dynamics), and that for the family variance (53% smaller in the model with no dynamics). Fourteen differences are less than 50% but at least 10%; twelve are less than 10% but at least 5%; eight are less than 5% but at least 1%; and the remaining fourteen are all less than 1%.

The differences for the thresholds and fixed intercepts are all less than 1%, and larger in the model with no dynamics. The largest difference for the actor variances is for younger children (10% larger in the model with no dynamics), and the smallest is for mothers (3% larger in the model with no dynamics). The partner variances are 4% (mothers and fathers) or 5% (older and younger children) larger in the model with no dynamics. The largest difference for the dyad-type variances is for older children acting towards mothers (24% larger in the model with no dynamics), and the smallest is for mothers acting towards fathers (6% larger in the model with no dynamics). The largest difference for the generalised reciprocities is for fathers, as already mentioned, and the smallest is for younger children (5% larger in the model with no dynamics). The largest difference for the dyadic reciprocities is for fathers and older children (11% larger in the model with no dynamics), and the smallest is for mothers and fathers (4% larger in the model with no dynamics). The largest difference for the correlation between innovations at the same snapshot for dyad types involving the same two family roles is for mothers and fathers, as already mentioned, and the smallest is for older and younger siblings (6% smaller in the model with no dynamics).

If we take the chains of parameter estimates and use these to calculate chains of differences between corresponding parameters in each pair of models (the model with full dynamics vs. the model with autoregressive effects only; the model with full dynamics vs. the model with no dynamics; and the model with autoregressive effects only vs. the model with no dynamics), we find point estimates similar to the differences between the point estimates for the two models, and credible intervals which include zero in every case.

For parameters common to all three models, the patterns of significance are similar across all models: in virtually all cases, parameters which are significant in the model with no dynamics are also significant in the model with full dynamics and in the model with autoregressive effects only, and in all cases parameters which are not significant in the model with no dynamics are not significant in the model with full dynamics or the model with autoregressive effects either. The exceptions to the former are the generalised reciprocity for younger children, which is significant in the model with no dynamics (though the lower limit of the credible interval is not far from zero) but non-significant in the model with full dynamics and the model with autoregressive effects only, the dyadic reciprocity for older and younger siblings, which is also significant in the model with no dynamics but not in the model with full dynamics or the model with autoregressive effects only, and the dyadic reciprocity for mothers and older children, which is significant in the model with no dynamics and the model with autoregressive effects only but not in the model with full dynamics.

The three models agree on the ordering of the point estimates across dyad types for the fixed intercepts; across family roles for the actor variances, for the partner variances, and for the generalised reciprocities; and across undirected dyad types for the dyadic reciprocities. For the dyad-type variances, the models nearly agree on the ordering, with the only point of difference concerning fathers acting towards mothers and younger children acting towards fathers, which are adjacent in the ordering in both models, but with younger children acting towards fathers estimated as the larger of the two in the model with no dynamics and fathers acting towards mothers in the model with full dynamics. The models also nearly agree on the ordering of the correlations between the innovations at the same snapshot for dyads involving the same two family roles, with the single point of difference in this case regarding fathers and older children, and mothers and older children; again, these two undirected dyad types are adjacent in the ordering for both models, with the correlation for mothers and older children being estimated as the larger of the two in the model with no dynamics and the correlation for fathers and older children in the model with full dynamics.

Table S13: Proportions of total variance attributable to each component

	Ordinal SRM			Ordinal DSRM (no crosslags)			Ordinal DSRM		
	Mean	Quantiles		Mean	Quantiles		Mean	Quantiles	
		2.5%	97.5%		2.5%	97.5%		2.5%	97.5%
Family (M▸F)	0.009	0.000	0.038	0.018	0.000	0.068	0.021	0.000	0.073
Family (F▸M)	0.009	0.000	0.039	0.019	0.000	0.070	0.021	0.000	0.075
Family (M▸S_1)	0.007	0.000	0.031	0.015	0.000	0.055	0.017	0.000	0.060

(cont.)

(cont.)

	Ordinal SRM			Ordinal DSRM (no crosslags)			Ordinal DSRM		
	Mean	Quantiles		Mean	Quantiles		Mean	Quantiles	
		2.5%	97.5%		2.5%	97.5%		2.5%	97.5%
Family (S_1▶M)	0.008	0.000	0.034	0.017	0.000	0.063	0.019	0.000	0.067
Family (M▶S_2)	0.007	0.000	0.032	0.016	0.000	0.059	0.018	0.000	0.063
Family (S_2▶M)	0.008	0.000	0.035	0.017	0.000	0.065	0.020	0.000	0.070
Family (F▶S_1)	0.008	0.000	0.034	0.016	0.000	0.061	0.019	0.000	0.066
Family (S_1▶F)	0.009	0.000	0.041	0.020	0.000	0.074	0.023	0.000	0.079
Family (F▶S_2)	0.008	0.000	0.036	0.017	0.000	0.065	0.020	0.000	0.070
Family (S_2▶F)	0.010	0.000	0.042	0.020	0.000	0.075	0.023	0.000	0.080
Family (S_1▶S_2)	0.009	0.000	0.041	0.020	0.000	0.074	0.023	0.000	0.079
Family (S_2▶S_1)	0.008	0.000	0.038	0.018	0.000	0.068	0.021	0.000	0.074
Actor (M▶F)	0.143	0.091	0.199	0.139	0.088	0.195	0.137	0.087	0.193
Actor (F▶M)	0.136	0.090	0.190	0.131	0.084	0.186	0.128	0.080	0.184
Actor (M▶S_1)	0.114	0.071	0.162	0.112	0.069	0.161	0.111	0.069	0.160
Actor (S_1▶M)	0.082	0.051	0.119	0.077	0.046	0.115	0.076	0.046	0.114
Actor (M▶S_2)	0.120	0.074	0.173	0.118	0.073	0.171	0.117	0.072	0.170
Actor (S_2▶M)	0.069	0.041	0.104	0.067	0.039	0.104	0.066	0.038	0.102
Actor (F▶S_1)	0.117	0.075	0.167	0.115	0.072	0.166	0.113	0.068	0.164
Actor (S_1▶F)	0.100	0.062	0.142	0.092	0.055	0.135	0.091	0.055	0.133
Actor (F▶S_2)	0.126	0.081	0.179	0.123	0.077	0.178	0.120	0.073	0.175
Actor (S_2▶F)	0.084	0.052	0.123	0.079	0.047	0.119	0.077	0.045	0.116
Actor (S_1▶S_2)	0.100	0.063	0.141	0.091	0.056	0.133	0.090	0.055	0.132
Actor (S_2▶S_1)	0.074	0.045	0.110	0.071	0.042	0.108	0.070	0.041	0.106
Partner (M▶F)	0.044	0.027	0.067	0.042	0.026	0.064	0.042	0.026	0.064
Partner (F▶M)	0.061	0.037	0.094	0.060	0.035	0.092	0.059	0.035	0.091
Partner (M▶S_1)	0.066	0.038	0.102	0.064	0.037	0.100	0.064	0.037	0.099
Partner (S_1▶M)	0.054	0.032	0.083	0.054	0.031	0.084	0.053	0.031	0.082
Partner (M▶S_2)	0.053	0.031	0.082	0.051	0.030	0.080	0.051	0.029	0.079
Partner (S_2▶M)	0.055	0.032	0.085	0.055	0.032	0.087	0.055	0.032	0.086
Partner (F▶S_1)	0.073	0.042	0.112	0.071	0.041	0.110	0.071	0.040	0.109
Partner (S_1▶F)	0.047	0.029	0.072	0.046	0.028	0.070	0.046	0.028	0.069
Partner (F▶S_2)	0.059	0.035	0.091	0.058	0.033	0.089	0.057	0.033	0.088
Partner (S_2▶F)	0.049	0.030	0.074	0.047	0.029	0.071	0.046	0.029	0.070
Partner (S_1▶S_2)	0.068	0.041	0.103	0.065	0.038	0.100	0.065	0.038	0.099
Partner (S_2▶S_1)	0.081	0.047	0.123	0.080	0.046	0.121	0.079	0.046	0.121
Dyad type (M▶F)	0.115	0.063	0.181	0.109	0.059	0.174	0.108	0.059	0.172
Dyad type (F▶M)	0.093	0.051	0.149	0.085	0.046	0.140	0.086	0.046	0.140
Dyad type (M▶S_1)	0.265	0.193	0.340	0.252	0.179	0.327	0.250	0.178	0.325
Dyad type (S_1▶M)	0.247	0.180	0.318	0.219	0.149	0.294	0.219	0.151	0.293
Dyad type (M▶S_2)	0.242	0.170	0.317	0.229	0.154	0.306	0.225	0.152	0.304
Dyad type (S_2▶M)	0.242	0.175	0.313	0.205	0.135	0.280	0.204	0.134	0.280
Dyad type (F▶S_1)	0.198	0.123	0.282	0.182	0.106	0.266	0.181	0.106	0.265
Dyad type (S_1▶F)	0.102	0.056	0.163	0.089	0.048	0.146	0.089	0.049	0.146
Dyad type (F▶S_2)	0.160	0.092	0.240	0.143	0.077	0.221	0.142	0.076	0.221
Dyad type (S_2▶F)	0.093	0.053	0.146	0.087	0.049	0.139	0.087	0.049	0.139
Dyad type (S_1▶S_2)	0.080	0.045	0.126	0.074	0.041	0.120	0.072	0.040	0.116

(cont.)

(cont.)

	Ordinal SRM			Ordinal DSRM (no crosslags)			Ordinal DSRM		
	Mean	Quantiles		Mean	Quantiles		Mean	Quantiles	
		2.5%	97.5%		2.5%	97.5%		2.5%	97.5%
Dyad type (S_2▸S_1)	0.164	0.100	0.235	0.143	0.082	0.215	0.138	0.078	0.210
Innovation (M▸F)	0.690	0.625	0.750	0.692	0.624	0.754	0.692	0.624	0.755
Innovation (F▸M)	0.701	0.641	0.756	0.705	0.643	0.763	0.706	0.643	0.763
Innovation (M▸S_1)	0.548	0.494	0.600	0.557	0.501	0.612	0.558	0.502	0.614
Innovation (S_1▸M)	0.610	0.554	0.664	0.633	0.572	0.692	0.632	0.571	0.691
Innovation (M▸S_2)	0.578	0.525	0.629	0.586	0.530	0.640	0.589	0.533	0.643
Innovation (S_2▸M)	0.626	0.571	0.677	0.655	0.595	0.711	0.655	0.594	0.712
Innovation (F▸S_1)	0.604	0.539	0.664	0.616	0.549	0.679	0.617	0.551	0.680
Innovation (S_1▸F)	0.742	0.688	0.790	0.753	0.693	0.804	0.752	0.691	0.805
Innovation (F▸S_2)	0.647	0.585	0.705	0.659	0.597	0.719	0.660	0.596	0.719
Innovation (S_2▸F)	0.765	0.709	0.814	0.767	0.705	0.819	0.766	0.705	0.818
Innovation (S_1▸S_2)	0.743	0.691	0.791	0.749	0.692	0.800	0.751	0.693	0.803
Innovation (S_2▸S_1)	0.672	0.612	0.728	0.688	0.624	0.747	0.692	0.627	0.753

Table S14: Proportions of total dyad type variance attributable to each component

	Ordinal SRM			Ordinal DSRM (no crosslags)			Ordinal DSRM		
	Mean	Quantiles		Mean	Quantiles		Mean	Quantiles	
		2.5%	97.5%		2.5%	97.5%		2.5%	97.5%
Family (M▸F)	0.027	0.001	0.119	0.057	0.001	0.204	0.066	0.001	0.219
Family (F▸M)	0.029	0.001	0.129	0.062	0.001	0.221	0.071	0.001	0.239
Family (M▸S_1)	0.015	0.000	0.068	0.033	0.001	0.123	0.038	0.001	0.132
Family (S_1▸M)	0.020	0.001	0.087	0.045	0.001	0.166	0.052	0.001	0.175
Family (M▸S_2)	0.017	0.001	0.076	0.037	0.001	0.139	0.043	0.001	0.148
Family (S_2▸M)	0.021	0.001	0.094	0.050	0.001	0.182	0.057	0.001	0.193
Family (F▸S_1)	0.019	0.001	0.086	0.042	0.001	0.155	0.049	0.001	0.170
Family (S_1▸F)	0.035	0.001	0.155	0.077	0.002	0.267	0.088	0.002	0.284
Family (F▸S_2)	0.023	0.001	0.102	0.050	0.001	0.185	0.058	0.001	0.198
Family (S_2▸F)	0.040	0.001	0.170	0.083	0.002	0.284	0.095	0.002	0.302
Family (S_1▸S_2)	0.036	0.001	0.159	0.076	0.002	0.271	0.088	0.002	0.287
Family (S_2▸S_1)	0.025	0.001	0.114	0.057	0.001	0.207	0.067	0.001	0.226
Actor (M▸F)	0.461	0.304	0.614	0.453	0.293	0.611	0.448	0.288	0.609
Actor (F▸M)	0.455	0.311	0.599	0.446	0.292	0.596	0.437	0.278	0.592
Actor (M▸S_1)	0.252	0.155	0.362	0.254	0.155	0.368	0.252	0.155	0.364
Actor (S_1▸M)	0.211	0.127	0.312	0.212	0.122	0.325	0.209	0.120	0.320
Actor (M▸S_2)	0.286	0.174	0.412	0.287	0.172	0.419	0.286	0.172	0.418
Actor (S_2▸M)	0.186	0.106	0.286	0.197	0.109	0.314	0.193	0.107	0.308
Actor (F▸S_1)	0.298	0.185	0.425	0.301	0.182	0.438	0.296	0.175	0.433
Actor (S_1▸F)	0.388	0.239	0.545	0.375	0.218	0.539	0.368	0.217	0.533
Actor (F▸S_2)	0.358	0.224	0.510	0.363	0.220	0.521	0.356	0.209	0.516
Actor (S_2▸F)	0.359	0.222	0.507	0.341	0.196	0.497	0.332	0.192	0.492

(cont.)

(cont.)

	Ordinal SRM			Ordinal DSRM (no crosslags)			Ordinal DSRM		
	Mean	Quantiles		Mean	Quantiles		Mean	Quantiles	
		2.5%	97.5%		2.5%	97.5%		2.5%	97.5%
Actor (S_1▸S_2)	0.389	0.255	0.526	0.366	0.226	0.513	0.363	0.222	0.514
Actor (S_2▸S_1)	0.227	0.133	0.344	0.229	0.129	0.354	0.229	0.128	0.355
Partner (M▸F)	0.142	0.084	0.221	0.139	0.080	0.218	0.138	0.080	0.216
Partner (F▸M)	0.206	0.120	0.314	0.204	0.118	0.313	0.202	0.115	0.314
Partner (M▸S_1)	0.147	0.083	0.231	0.146	0.082	0.229	0.145	0.081	0.229
Partner (S_1▸M)	0.138	0.079	0.216	0.147	0.083	0.234	0.145	0.081	0.231
Partner (M▸S_2)	0.126	0.072	0.199	0.125	0.069	0.200	0.124	0.070	0.199
Partner (S_2▸M)	0.148	0.084	0.233	0.162	0.090	0.261	0.160	0.089	0.258
Partner (F▸S_1)	0.186	0.103	0.292	0.187	0.103	0.297	0.186	0.101	0.297
Partner (S_1▸F)	0.184	0.109	0.280	0.188	0.111	0.291	0.186	0.109	0.287
Partner (F▸S_2)	0.169	0.095	0.264	0.171	0.095	0.270	0.169	0.094	0.269
Partner (S_2▸F)	0.208	0.123	0.319	0.204	0.119	0.314	0.201	0.118	0.310
Partner (S_1▸S_2)	0.265	0.159	0.391	0.262	0.153	0.394	0.261	0.152	0.391
Partner (S_2▸S_1)	0.249	0.143	0.376	0.257	0.145	0.391	0.259	0.146	0.395
Dyad type (M▸F)	0.369	0.220	0.533	0.351	0.203	0.517	0.349	0.204	0.511
Dyad type (F▸M)	0.310	0.179	0.460	0.289	0.164	0.441	0.290	0.164	0.442
Dyad type (M▸S_1)	0.585	0.453	0.707	0.566	0.429	0.693	0.564	0.426	0.691
Dyad type (S_1▸M)	0.631	0.507	0.743	0.596	0.449	0.724	0.595	0.449	0.724
Dyad type (M▸S_2)	0.571	0.428	0.704	0.551	0.395	0.693	0.546	0.391	0.688
Dyad type (S_2▸M)	0.646	0.510	0.761	0.591	0.431	0.731	0.590	0.428	0.730
Dyad type (F▸S_1)	0.497	0.338	0.645	0.470	0.304	0.627	0.469	0.304	0.626
Dyad type (S_1▸F)	0.393	0.236	0.565	0.360	0.209	0.533	0.358	0.207	0.532
Dyad type (F▸S_2)	0.450	0.283	0.613	0.416	0.248	0.587	0.416	0.244	0.588
Dyad type (S_2▸F)	0.393	0.245	0.551	0.373	0.225	0.535	0.372	0.223	0.537
Dyad type (S_1▸S_2)	0.310	0.185	0.457	0.296	0.171	0.445	0.287	0.165	0.436
Dyad type (S_2▸S_1)	0.498	0.337	0.647	0.457	0.289	0.618	0.446	0.278	0.610

The VPCs show slight differences between the three models, with (going by point estimates) the family effects and innovations generally accounting for a slightly greater proportion in the model with full dynamics and the model with autoregressive effects only than in the model with no dynamics and the other components accounting for a slightly smaller proportion. The differences between the model with full dynamics and the model with autoregressive effects only are even slighter; the family effects and innovations generally account for a greater proportion in the model with full dynamics and the actor and dyad-type effects for a smaller proportion, while the partner effects account for the same proportion to the precision we calculate for around half the dyad types and for a greater proportion in the model with autoregressive effects only in the rest.

The broad pattern, however, is the same across all three models. The family effects account for very little variation (1% at most for the model with no dynamics, for older children acting towards fathers, 2% at most for the model with autoregressive effects only, for younger children acting towards fathers, older children acting towards fathers, and younger children acting towards older children, and a little over 2% at most for the model with full dynamics, for the same dyad types as the model with autoregressive effects only). The innovations account for most of the variance, ranging from 55% (for mothers acting towards younger children) to 77% (for older children acting towards fathers) for the model with no dynamics, and from 56% (again for mothers acting towards younger children) to 77% (again

for older children acting towards fathers) for the model with autoregressive effects only and the model with full dynamics. The ordering of the other three components is less clear; while going by point estimates the partner effects provide the smallest contribution of the three for all dyad types except older children acting towards younger children (for which its contribution is the second smallest after that of the actor effects) and for most dyad types (all except mothers acting towards fathers, fathers acting towards mothers, and younger siblings acting towards older siblings, and for the model with full dynamics and the model with autoregressive effects only also younger children acting towards fathers, for which the contribution of the actor effect is the largest) the dyad-type effects provide the largest, the credible intervals for the contributions of the actor effects overlap those for the partner effects for all but one dyad type (mothers acting towards fathers), in some cases only slightly but in some cases considerably, and they also overlap those for the dyad type effects for three quarters of the dyad types (including mothers acting towards fathers), again in some cases only slightly but in others considerably. The credible intervals for the contribution of the partner effects also overlap those for the contribution of the family effects for all dyad types for the model with full dynamics and the model with autoregressive effects only, and for over half the dyad types for the model with no dynamics. The contribution of the actor effects ranges from 7% (for older children acting towards mothers) to 14% (for mothers acting towards fathers) for all models; the contribution of the partner effects ranges from 4% (for mothers acting towards fathers) to 8% (for older siblings acting towards younger siblings) for all models; and the contribution of the dyad-type effects ranges from 7% (the model with autoregressive effects only and the model with full dynamics) or 8% (the model with no dynamics; in all cases for younger siblings acting towards older siblings) to 25% (the model with autoregressive effects only and the model with full dynamics) or 27% (the model with no dynamics; in all cases for mothers acting towards younger children).

The contributions to the total dyad-type variance also show slight differences between the three models, again greater between the model with no dynamics and the model with full dynamics and between the model with no dynamics and the model with autoregressive effects only than between the model with autoregressive effects only and the model with full dynamics. The contributions of the family effects are generally higher in the model with autoregressive effects only than in the model with no dynamics, and higher again in the model with full dynamics. The contributions of the actor effects are generally higher in the model with no dynamics than in the model with full dynamics, and in the model with autoregressive effects only than in the model with full dynamics, but are about equally often higher in the model with no dynamics than in the model with autoregressive effects only and higher in the model with autoregressive effects only than in the model with no dynamics. The latter is also the case for the contribution of the partner effects, and their contribution is also equally often higher in the model with no dynamics than in the model with full dynamics and either higher or the same (to the precision we calculate them) in the model with full dynamics than in the model with no dynamics; but their contribution is generally higher in the model with autoregressive effects only than in the model with full dynamics. The contribution of the dyad-type effects is generally larger in the model with autoregressive effects only than in the model with full dynamics, and larger again in the model with no dynamics.

Again, the broad pattern is the same across all three models. The family effects again account for little variation (ranging from 2%, for mothers acting towards younger children, to 4%, for older children acting towards fathers, for the model with no dynamics; from 3%, for mothers acting towards younger children, to 8%, for older children acting towards fathers, for the model with autoregressive effects only; and from 4%, for mothers acting towards younger children, to 10%, for older children acting towards fathers, for the model with full dynamics). Going by the point estimates, again the partner effects provide the next smallest contribution for all dyad types except older siblings acting towards younger siblings, and the dyad-type effects provide the largest contribution for all dyad types except mothers acting towards fathers, fathers acting towards mothers, and younger siblings acting towards older siblings, and for the model with autoregressive effects only and the model with full dynamics also younger children acting towards fathers. But again, there is overlap between credible intervals in many cases. The intervals for the contribution of the family effects overlap those for the partner effects for more than half the dyad types for the model with no dynamics, for nearly half of the dyad types for the model with autoregressive effects only, and for half the dyad types for the model with full dynamics. The intervals for the contribution of the actor effects overlap those for the partner effects for every dyad type except mothers acting towards fathers in all three models, in some cases considerably, and overlap those for the dyad-type effects for two thirds of the dyad types for the model with no dynamics and for three quarters of the dyad types for the model with full dynamics (including in all models mothers acting towards fathers), again in some cases considerably. The contribution of the actor effects ranges from

Table S15: Correlations between responses at successive snapshots in ordinal dynamic SRM

	Mean	S.D.	Quantiles				
			2.5%	25%	50%	75%	97.5%
M▸F	0.353	0.043	0.270	0.325	0.354	0.382	0.436
F▸M	0.430	0.038	0.355	0.405	0.430	0.456	0.502
M▸S_1	0.492	0.030	0.433	0.472	0.492	0.512	0.549
S_1▸M	0.500	0.029	0.442	0.480	0.500	0.520	0.556
M▸S_2	0.468	0.030	0.409	0.448	0.468	0.488	0.527
S_2▸M	0.490	0.028	0.435	0.471	0.490	0.509	0.545
F▸S_1	0.433	0.038	0.357	0.407	0.433	0.458	0.506
S_1▸F	0.398	0.037	0.325	0.374	0.398	0.423	0.470
F▸S_2	0.408	0.036	0.336	0.384	0.408	0.432	0.479
S_2▸F	0.323	0.041	0.244	0.296	0.323	0.351	0.402
S_1▸S_2	0.351	0.040	0.271	0.324	0.352	0.379	0.429
S_2▸S_1	0.416	0.039	0.339	0.390	0.416	0.442	0.491

19% (in the model with no dynamics and the model with full dynamics) or 20% (in the model with autoregressive effects only), for older children acting towards mothers to 45% (in the model with autoregressive effects only and the model with full dynamics) or 46% (in the model with no dynamics), for mothers acting towards fathers. The contribution of the partner effects ranges from 12% (in the model with full dynamics) or 13% (in the model with autoregressive effects only and the model with no dynamics), for mothers acting towards older children, to 26% (in the model with autoregressive effects only and the model with full dynamics) or 27% (in the model with no dynamics), for younger siblings acting towards older siblings. The contribution of the dyad-type effects ranges from 31% (for fathers acting towards mothers and for younger siblings acting towards older siblings) to 65% (for older children acting towards mothers) in the model with no dynamics; from 29% (for fathers acting towards mothers) to 60% (for younger children acting towards mothers) in the model with autoregressive effects only; and from 29% (for younger siblings acting towards older siblings) to 60% (for younger children acting towards mothers) in the model with full dynamics.

S5.3 Further results for the Dynamic Social Relations Model

In this section, we provide some supplementary results for the model we focus on, the ordinal social relations model with full dynamics. These are the correlations between the responses at successive snapshots, the total correlation between each pair of dyad types, and the differences between each pair of directed or undirected dyad types or family roles for various parameters of the model. As described in Section S3.5, these are all calculated using the saved parameter chains.

The correlations between the responses at successive snapshots are shown in Table S15. The correlations are moderate for all dyad types. Going by the point estimates, the correlation is strongest (at 0.500) for younger children acting towards mothers and weakest (at 0.323) for older children acting towards fathers. The credible intervals are reasonably wide.

The total dyad-type correlations are shown in Table S18 and are laid out in the form of a correlation matrix, but instead of the correlations of 1 running down the diagonal, the total dyad type variance (i.e. the sum of all components of variance other than the innovation) for each dyad type is shown instead. Each cell is shaded according to the strength of the correlation and according to the relationship between the two dyad types concerned, as described in Tables S16 and S17.

Table S16: Colours used for different types of correlation

A	Between the same two people (with actor and partner roles each way round)
B	Between different dyads with the same actor
C	Between different dyads with the same partner
D	Between different dyads sharing one person who is the actor in one and the partner in the other
E	Between different dyads consisting entirely of different people

Table S17: Shadings used to indicate type and strength of correlation

Absolute value	A	B	C	D	E
0.6 or more					
0.5 to 0.6					
0.4 to 0.5					
0.3 to 0.4					
0.2 to 0.3					
Less than 0.2					

There is quite a difference between dyad types in the total dyad type variance, with the point estimates ranging from 0.307 for older children acting towards fathers to 0.796 for mothers acting towards younger children. The correlations also vary considerably between pairs of dyad types, from extremely weak correlations, many of which are non-significant, with the lowest point estimate being 0.047, for the correlation between fathers acting towards older children and mothers acting towards younger children, to moderate correlations, with the highest point estimate being 0.458, for the correlation between fathers acting towards mothers and fathers acting towards younger children. Not surprisingly, considering how these correlations are calculated, the correlations between pairs of dyad types which do not share a family role and between pairs of dyad types which share exactly one family role, which is the actor in one and the partner in the other, are all weak and in many cases non-significant (and account for the majority of weak and non-significant correlations). As expected, the correlations between pairs of dyad types which share exactly one family role which is the partner in both are (with the exception of the weak correlation of 0.195 for mothers and fathers acting towards older children) moderately low, ranging from 0.206 for younger and older siblings children towards mothers to 0.284 for younger and older children acting towards fathers, and the correlations between pairs of dyad types which share exactly one family role which is the actor in both are moderately low to moderate, ranging from 0.271 for older children towards mothers and younger children to 0.458 for fathers towards mothers and older children. Less expected is that two of the six correlations between pairs of dyad types involving the same undirected dyad (mothers and fathers, and fathers and older children) are weak and non-significant, although the remaining four are moderately low to moderate, ranging from 0.235 for fathers and older children to 0.423 for mothers and younger children.

Table S18: Estimated total correlations and variances from Ordinal Dynamic Social Relations model

	M▸F	F▸M	M▸S_1	S_1▸M	M▸S_2	S_2▸M	F▸S_1	S_1▸F	F▸S_2	S_2▸F	S_1▸S_2	S_2▸S_1
M▸F	0.448 (0.325,0.602)											
F▸M	0.190 (−0.016,0.386)	0.420 (0.310,0.555)										
M▸S_1	0.385 (0.263,0.507)	0.092 (−0.010,0.220)	0.796 (0.629,0.992)									
S_1▸M	0.103 (−0.012,0.242)	0.231 (0.130,0.361)	0.423 (0.273,0.559)	0.585 (0.448,0.752)								
M▸S_2	0.410 (0.278,0.542)	0.098 (−0.011,0.231)	0.308 (0.197,0.430)	0.083 (−0.009,0.199)	0.703 (0.556,0.877)							
S_2▸M	0.109 (−0.012,0.254)	0.243 (0.136,0.381)	0.082 (−0.009,0.198)	0.206 (0.113,0.329)	0.305 (0.142,0.456)	0.530 (0.405,0.682)						
F▸S_1	0.066 (−0.034,0.203)	0.417 (0.291,0.546)	0.207 (0.117,0.318)	0.111 (0.022,0.228)	0.046 (0.001,0.156)	0.053 (0.001,0.179)	0.624 (0.470,0.815)					
S_1▸F	0.235 (0.128,0.390)	0.092 (−0.049,0.274)	0.129 (0.027,0.257)	0.343 (0.218,0.480)	0.062 (0.001,0.202)	0.071 (0.001,0.231)	0.180 (−0.023,0.374)	0.332 (0.243,0.446)				
F▸S_2	0.072 (−0.037,0.219)	0.458 (0.319,0.594)	0.047 (0.001,0.160)	0.055 (0.001,0.184)	0.195 (0.107,0.311)	0.124 (0.031,0.255)	0.377 (0.248,0.511)	0.083 (−0.044,0.250)	0.518 (0.391,0.678)			
S_2▸F	0.244 (0.133,0.404)	0.095 (−0.051,0.283)	0.060 (0.001,0.198)	0.070 (0.001,0.227)	0.139 (0.037,0.273)	0.326 (0.202,0.468)	0.079 (−0.041,0.238)	0.284 (0.156,0.461)	0.235 (0.028,0.428)	0.307 (0.222,0.419)		
S_1▸S_2	0.076 (0.002,0.246)	0.079 (0.002,0.257)	0.129 (0.027,0.257)	0.342 (0.221,0.476)	0.241 (0.140,0.369)	0.154 (0.040,0.305)	0.146 (0.030,0.289)	0.452 (0.306,0.598)	0.281 (0.165,0.427)	0.200 (0.055,0.377)	0.334 (0.245,0.444)	
S_2▸S_1	0.066 (0.001,0.219)	0.069 (0.001,0.228)	0.244 (0.142,0.362)	0.131 (0.026,0.263)	0.116 (0.030,0.236)	0.271 (0.160,0.407)	0.275 (0.160,0.407)	0.172 (0.036,0.333)	0.135 (0.035,0.271)	0.354 (0.225,0.496)	0.380 (0.193,0.546)	0.448 (0.329,0.595)

Table S19: Shadings used to indicate direction and magnitude of differences between parameters except autoregressive effects, cross-lagged effects, and actor and partner effects and generalised reciprocities

Absolute value	Dyad type in column > dyad type in row by...	Dyad type in column < dyad type in row by...
0.5 or more		
0.4 to 0.5		
0.3 to 0.4		
0.2 to 0.3		
0.1 to 0.2		
Less than 0.1		

Table S20 shows the differences between the fixed intercepts for each pair of dyad types, with each cell showing the result of subtracting the intercept for the dyad type in that row from the intercept for the dyad type in that column. Table S19 explains the colours that are used to shade the cells in this table. These shadings are applied only when the 95% credible interval for the difference in question does not include 0.

At the bottom end of the scale, the intercept for younger children acting towards mothers is significantly smaller than the intercept for every other dyad type, with the largest point estimate of the absolute difference between this and any other dyad type's intercept being 1.636, for fathers acting towards younger children. Also smaller than most other dyad types' intercepts, with the differences from every dyad type other than younger children acting towards mothers either showing them to be significantly smaller or not significantly different, are older children acting towards mothers (largest difference 1.258, again from fathers acting towards younger children), younger children acting towards fathers (largest difference 1.303, again from fathers acting towards younger children), older children acting towards fathers (largest difference 1.244, again from fathers acting towards younger children), and younger children acting towards older children (largest difference 1.358, again for fathers acting towards younger children).

At the other end of the scale, there is no dyad type whose intercept is significantly larger than the intercept for every other dyad type, but there are three dyad types whose intercepts are significantly larger than the intercept for every dyad type other than those three, whose intercepts are not significantly different from each other. These are mothers acting towards younger children (for which the largest difference is 1.458, from younger children acting towards mothers), mothers acting towards older children (for which the largest difference is 1.451, again from younger children acting towards mothers), and fathers acting towards younger children (for which the largest difference is 1.636, again from younger children acting towards mothers).

For most pairs of dyad types, the intercept of one is significantly bigger than the intercept of the other. There are only twelve pairs of dyad types whose intercepts cannot be distinguished from each other. Nevertheless, there are only two dyad types whose intercepts can be distinguished from every other dyad type: fathers acting towards mothers and younger children acting towards mothers. Every other dyad type's intercept is indistinguishable from that of at least one other dyad type. The intercepts for older children acting towards mothers and older children acting towards fathers are indistinguishable from the greatest number of intercepts of other dyad types (four), with younger children acting towards fathers next (at three).

Table S20: Estimated differences between fixed intercepts from Ordinal Dynamic Social Relations model

	M▶F	F▶M	M▶S_1	S_1▶M	M▶S_2	S_2▶M	F▶S_1	S_1▶F	F▶S_2	S_2▶F	S_1▶S_2	S_2▶S_1
M▶F												
F▶M	0.229 (0.062,0.398)											
M▶S_1	−0.352 (−0.511,−0.192)	−0.581 (−0.760,−0.403)										
S_1▶M	1.106 (0.936,1.274)	0.876 (0.715,1.040)	1.458 (1.306,1.607)									
M▶S_2	−0.345 (−0.497,−0.194)	−0.574 (−0.747,−0.401)	0.007 (−0.152,0.165)	−1.451 (−1.620,−1.280)								
S_2▶M	0.728 (0.565,0.891)	0.499 (0.342,0.658)	1.080 (0.909,1.251)	−0.377 (−0.531,−0.224)	1.073 (0.928,1.218)							
F▶S_1	−0.530 (−0.717,−0.342)	−0.759 (−0.921,−0.596)	−0.178 (−0.357,0.004)	−1.636 (−1.817,−1.454)	−0.185 (−0.373,0.002)	−1.258 (−1.439,−1.075)						
S_1▶F	0.773 (0.613,0.929)	0.543 (0.376,0.713)	1.125 (0.953,1.294)	−0.333 (−0.482,−0.183)	1.118 (0.948,1.287)	0.045 (−0.115,0.204)	1.303 (1.131,1.473)					
F▶S_2	−0.125 (−0.305,0.055)	−0.354 (−0.508,−0.202)	0.227 (0.043,0.412)	−1.230 (−1.407,−1.052)	0.221 (0.051,0.391)	−0.853 (−1.021,−0.685)	0.406 (0.237,0.572)	−0.897 (−1.071,−0.724)				
S_2▶F	0.713 (0.559,0.866)	0.484 (0.322,0.647)	1.065 (0.894,1.238)	−0.392 (−0.553,−0.233)	1.059 (0.899,1.216)	−0.015 (−0.157,0.126)	1.244 (1.064,1.421)	−0.059 (−0.206,0.086)	0.838 (0.679,0.994)			
S_1▶S_2	0.828 (0.666,0.989)	0.599 (0.437,0.762)	1.180 (1.016,1.344)	−0.277 (−0.420,−0.134)	1.173 (1.020,1.325)	0.100 (−0.049,0.248)	1.358 (1.182,1.532)	0.055 (−0.084,0.197)	0.953 (0.791,1.112)	0.115 (−0.030,0.260)		
S_2▶S_1	0.596 (0.427,0.763)	0.367 (0.197,0.535)	0.948 (0.786,1.112)	−0.509 (−0.669,−0.350)	0.941 (0.777,1.104)	−0.132 (−0.282,0.015)	1.126 (0.953,1.297)	−0.177 (−0.333,−0.021)	0.721 (0.552,0.891)	−0.117 (−0.261,0.029)	−0.232 (−0.358,−0.105)	

Table S21: Shadings used to indicate direction and magnitude of differences between autoregressive effects, cross-lagged effects, and actor and partner effects and generalised reciprocities

Absolute value	Dyad type in column > dyad type in row by...	Dyad type in column < dyad type in row by...
0.150 or more		
0.125 to 0.150		
0.100 to 0.125		
0.075 to 0.100		
0.050 to 0.075		
Less than 0.050		

Table S22 also shows differences in the same way as Table S20, but for the autoregressive effects this time. The shading used for this table is different to the shading used for Table S20, reflecting the fact that what might be considered a big difference in autoregressive effects is probably different to what might be considered a big difference in intercepts, and is shown in Table S21. Again, this shading is applied only for differences whose 95% credible interval excludes 0.

Unlike for the fixed intercepts, most of the differences (all but fourteen) between autoregressive effects are non-significant, meaning that most pairs of dyad types' autoregressive effects cannot be distinguished from each other. Still, there are a few dyad types whose autoregressive effects are either indistinguishable from or significantly bigger than the autoregressive effect of every other dyad type, and a few whose autoregressive effects are either indistinguishable from significantly smaller than the autoregressive effect of every other dyad type. Among the first group are older children acting towards mothers (significantly bigger than six other dyad types); younger children acting towards mothers (significantly bigger than five other dyad types); younger children acting towards fathers (significantly bigger than two other dyad types); and fathers acting towards mothers (significantly bigger than one other dyad type). Among the second group are mothers acting towards fathers (significantly smaller than four other dyad types); mothers acting towards younger children (significantly smaller than three other dyad types); mothers acting towards older children, fathers acting towards younger children and fathers acting towards older children (each significantly smaller than two other dyad types); and older children acting towards fathers (significantly smaller than one other dyad type). We should note that while the estimates of the autoregressive for all the dyad types in the first group were significant, the second group includes the two dyad types for which they were not significant (mothers acting towards fathers and fathers acting towards younger children), and the significant difference we find between all dyad types in the first group and mothers acting towards fathers, and between two of the dyad types in the first group and fathers acting towards younger children, may reflect there being no autoregressive effect for mothers acting towards fathers or fathers acting towards younger children and our already having established the autoregressive effects for the dyad types in the first group are significantly different from zero, rather than the autoregressive effects for dyad types in the first group being larger than autoregressive effects for mothers acting towards fathers and/or fathers acting towards younger children which exist but are too small for us to have detected as significant.

As expected, the differences are generally smaller than the differences between the fixed intercepts, with the largest point estimate being 0.169 (the amount by which the autoregressive effect for older children acting towards mothers is larger than the autoregressive effect for mothers acting towards fathers), in contrast to the largest point estimate of 1.636 (the amount by which the intercept for fathers acting towards younger children is larger than the intercept for younger children acting towards mothers). The point estimate of the smallest significant difference is also smaller for the autoregressive effects (at 0.114, the amount by which the autoregressive effect for younger children acting towards fathers is larger than the autoregressive effect for mothers acting towards younger children) than for the intercepts (at 0.227, the amount by which the intercept for mothers acting towards younger children is larger than the intercept for fathers acting towards older children). This is in line with what we saw from the estimates of the intercepts and autoregressive effects: smaller differences between the point estimates for different dyad types and more precise estimation (narrower credible intervals) for the autoregressive effects than the intercepts.

Table S22: Estimated differences between fixed lags from Ordinal Dynamic Social Relations model

	M▸F	F▸M	M▸S_1	S_1▸M	M▸S_2	S_2▸M	F▸S_1	S_1▸F	F▸S_2	S_2▸F	S_1▸S_2	S_2▸S_1
M▸F												
F▸M	−0.134 (−0.263,−0.003)											
M▸S_1	−0.027 (−0.141,0.089)	0.107 (−0.007,0.220)										
S_1▸M	−0.156 (−0.274,−0.036)	−0.022 (−0.138,0.096)	−0.129 (−0.229,−0.029)									
M▸S_2	−0.034 (−0.147,0.083)	0.100 (−0.016,0.214)	−0.007 (−0.102,0.088)	0.122 (0.020,0.223)								
S_2▸M	−0.169 (−0.283,−0.051)	−0.035 (−0.149,0.079)	−0.142 (−0.238,−0.045)	−0.013 (−0.114,0.087)	−0.135 (−0.234,−0.037)							
F▸S_1	−0.017 (−0.145,0.113)	0.117 (−0.012,0.246)	0.010 (−0.103,0.121)	0.139 (0.024,0.255)	0.017 (−0.097,0.130)	0.152 (0.039,0.267)						
S_1▸F	−0.140 (−0.267,−0.012)	−0.006 (−0.132,0.119)	−0.114 (−0.223,−0.004)	0.015 (−0.096,0.128)	−0.106 (−0.217,0.005)	0.029 (−0.084,0.141)	−0.124 (−0.250,0.002)					
F▸S_2	−0.039 (−0.164,0.086)	0.094 (−0.030,0.219)	−0.013 (−0.119,0.094)	0.116 (0.005,0.227)	−0.006 (−0.114,0.103)	0.129 (0.020,0.238)	−0.023 (−0.146,0.101)	0.101 (−0.020,0.221)				
S_2▸F	−0.052 (−0.179,0.077)	0.082 (−0.044,0.209)	−0.026 (−0.136,0.085)	0.103 (−0.011,0.219)	−0.018 (−0.131,0.094)	0.117 (0.004,0.230)	−0.035 (−0.163,0.091)	0.088 (−0.035,0.213)	−0.013 (−0.135,0.109)			
S_1▸S_2	−0.075 (−0.204,0.055)	0.059 (−0.070,0.188)	−0.048 (−0.161,0.065)	0.081 (−0.037,0.197)	−0.041 (−0.155,0.074)	0.094 (−0.020,0.209)	−0.058 (−0.187,0.070)	0.065 (−0.061,0.191)	−0.035 (−0.159,0.088)	−0.023 (−0.150,0.105)		
S_2▸S_1	−0.095 (−0.230,0.040)	0.039 (−0.095,0.171)	−0.069 (−0.187,0.048)	0.060 (−0.062,0.182)	−0.062 (−0.181,0.057)	0.074 (−0.045,0.193)	−0.079 (−0.212,0.053)	0.045 (−0.086,0.176)	−0.056 (−0.184,0.073)	−0.043 (−0.176,0.089)	−0.021 (−0.160,0.117)	

Table S23: Estimated differences between fixed crosslags from Ordinal Dynamic Social Relations model

	M▸F	F▸M	M▸S$_1$	S$_1$▸M	M▸S$_2$	S$_2$▸M	F▸S$_1$	S$_1$▸F	F▸S$_2$	S$_2$▸F	S$_1$▸S$_2$	S$_2$▸S$_1$
M▸F												
F▸M	−0.031 (−0.144,0.080)											
M▸S$_1$	0.030 (−0.091,0.151)	0.061 (−0.056,0.178)										
S$_1$▸M	−0.014 (−0.120,0.092)	0.017 (−0.085,0.118)	−0.044 (−0.136,0.047)									
M▸S$_2$	−0.080 (−0.195,0.034)	−0.049 (−0.158,0.061)	−0.110 (−0.217,−0.003)	−0.065 (−0.156,0.025)								
S$_2$▸M	−0.069 (−0.177,0.039)	−0.038 (−0.142,0.066)	−0.099 (−0.200,0.002)	−0.055 (−0.138,0.028)	0.011 (−0.075,0.097)							
F▸S$_1$	−0.040 (−0.172,0.091)	−0.009 (−0.138,0.118)	−0.070 (−0.196,0.055)	−0.026 (−0.140,0.086)	0.040 (−0.081,0.162)	0.029 (−0.086,0.143)						
S$_1$▸F	−0.025 (−0.141,0.092)	0.007 (−0.107,0.118)	−0.054 (−0.165,0.055)	−0.010 (−0.103,0.083)	0.055 (−0.046,0.158)	0.044 (−0.051,0.139)	0.015 (−0.102,0.133)					
F▸S$_2$	−0.007 (−0.141,0.126)	0.024 (−0.107,0.154)	−0.037 (−0.164,0.090)	0.007 (−0.106,0.119)	0.072 (−0.049,0.193)	0.062 (−0.051,0.176)	0.033 (−0.106,0.170)	0.017 (−0.105,0.139)				
S$_2$▸F	−0.026 (−0.139,0.089)	0.005 (−0.106,0.115)	−0.056 (−0.162,0.051)	−0.012 (−0.102,0.078)	0.054 (−0.046,0.153)	0.043 (−0.050,0.135)	0.014 (−0.107,0.135)	−0.001 (−0.105,0.102)	−0.019 (−0.131,0.094)			
S$_1$▸S$_2$	−0.123 (−0.243,−0.000)	−0.091 (−0.210,0.027)	−0.152 (−0.266,−0.038)	−0.108 (−0.208,−0.009)	−0.043 (−0.150,0.068)	−0.053 (−0.155,0.049)	−0.083 (−0.211,0.046)	−0.098 (−0.207,0.013)	−0.115 (−0.243,0.012)	−0.097 (−0.206,0.011)		
S$_2$▸S$_1$	−0.052 (−0.182,0.080)	−0.021 (−0.148,0.106)	−0.082 (−0.206,0.044)	−0.037 (−0.148,0.075)	0.028 (−0.089,0.147)	0.017 (−0.096,0.131)	−0.012 (−0.149,0.126)	−0.027 (−0.146,0.095)	−0.044 (−0.180,0.092)	−0.026 (−0.143,0.092)	0.071 (−0.051,0.192)	

Table S23 shows differences between the cross-lagged effects in the same way as Table S20 for the fixed intercepts or Table S22 for the autoregressive effects. The shading used is the same as for Table S22, shown in Table S21, and again is applied only to differences whose credible interval excludes 0.

Even fewer differences (only four, and one of these only just so) are significant than for the autoregressive effects, meaning that for the vast majority of pairs of dyad types the cross-lagged effect of one cannot be distinguished from the cross-lagged effect of the other. Still, there are two dyad types for which the cross-lagged effect is either significantly greater than or indistinguishable from the cross-lagged effect for every other dyad type (younger siblings acting towards older siblings, the only dyad type for which the estimate of the cross-lagged effect was significant, for which the cross-lagged effect is significantly greater than that of three other dyad types; and mothers acting towards older siblings, significantly greater than one other dyad type), and three dyad types for which the cross-lagged effect is either significantly smaller than or indistinguishable from the cross-lagged effect for every other dyad type (mothers acting towards younger siblings, significantly smaller than two other dyad types; and mothers acting towards fathers and younger siblings acting towards mothers, each significantly smaller than one other dyad type). Among those differences which are significant, the magnitudes are comparable to those of the significant differences between autoregressive effects, with the largest being 0.152 (the amount by which the cross-lagged effect for younger siblings acting towards older siblings is greater than the cross-lagged effect for mothers acting towards younger siblings) and the smallest 0.108 (the amount by which the cross-lagged effect for younger siblings acting towards older siblings is greater than the cross-lagged effect for younger siblings acting towards mothers).

Table S24 shows differences between the actor variances, partner variances, and generalised reciprocities in a similar way to Tables S20, S22, and S23 for the fixed intercepts, autoregressive effects, and cross-lagged effects respectively. Again, the shading used is that shown in Table S21, and is applied only to differences whose credible interval excludes 0.

Most of the parameters cannot be distinguished from each other. The main exception is in comparing actor and partner variances: the actor variances for mothers and fathers are larger than all but the younger children's partner variance, and the actor variance for younger children is larger than the partner variance for fathers. No actor variance is found to be significantly smaller than any partner variance, but the rest of the comparisons between actor and partner variances do not find the actor variance to be significantly larger than the partner variance either. The largest difference is 0.139 (the amount by which the mothers' actor variance is larger than the fathers' partner variance), and the smallest significant difference is 0.060 (the amount by which the younger children's actor variance is larger than the fathers' partner variance). Besides the differences of actor from partner variances, there is just one significant difference: the mothers' actor variance is significantly larger than the older children's actor variance (with a point estimate of 0.099).

Table S24: Estimated differences between actor and partner effects and generalised reciprocities from Ordinal Dynamic Social Relations model

		Actor effects M	Actor effects F	Actor effects S_1	Actor effects S_2	Partner effects M	Partner effects F	Partner effects S_1	Partner effects S_2	Generalised reciprocities M	Generalised reciprocities F	Generalised reciprocities S_1	Generalised reciprocities S_2
Actor effects	M												
Actor effects	F	0.017 (−0.101,0.136)											
Actor effects	S_1	0.079 (−0.022,0.186)	0.062 (−0.030,0.164)										
Actor effects	S_2	0.099 (0.006,0.202)	0.082 (−0.008,0.180)	0.020 (−0.052,0.093)									
Partner effects	M	0.116 (0.027,0.215)	0.099 (0.014,0.197)	0.037 (−0.030,0.108)	0.017 (−0.045,0.083)								
Partner effects	F	0.139 (0.055,0.237)	0.122 (0.043,0.216)	0.060 (0.001,0.126)	0.040 (−0.013,0.101)	0.023 (−0.025,0.077)							
Partner effects	S_1	0.085 (−0.016,0.192)	0.068 (−0.029,0.172)	0.006 (−0.069,0.080)	−0.014 (−0.089,0.060)	−0.031 (−0.104,0.037)	−0.054 (−0.123,0.006)						
Partner effects	S_2	0.113 (0.023,0.214)	0.097 (0.011,0.194)	0.034 (−0.033,0.105)	0.014 (−0.043,0.074)	−0.003 (−0.063,0.056)	−0.026 (−0.080,0.023)	0.028 (−0.040,0.101)					
Generalised reciprocities	M	0.029 (−0.278,0.380)	0.012 (−0.308,0.380)	−0.050 (−0.362,0.308)	−0.070 (−0.384,0.284)	−0.087 (−0.395,0.266)	−0.110 (−0.427,0.250)	−0.056 (−0.373,0.305)	−0.084 (−0.402,0.272)				
Generalised reciprocities	F	0.158 (−0.186,0.514)	0.142 (−0.189,0.486)	0.079 (−0.256,0.422)	0.060 (−0.278,0.404)	0.042 (−0.295,0.387)	0.019 (−0.314,0.364)	0.073 (−0.265,0.419)	0.045 (−0.290,0.389)	0.129 (−0.341,0.585)			
Generalised reciprocities	S_1	−0.102 (−0.390,0.244)	−0.119 (−0.406,0.228)	−0.181 (−0.448,0.149)	−0.201 (−0.482,0.144)	−0.218 (−0.499,0.122)	−0.241 (−0.520,0.100)	−0.187 (−0.456,0.141)	−0.215 (−0.496,0.128)	−0.131 (−0.585,0.325)	−0.261 (−0.713,0.214)		
Generalised reciprocities	S_2	−0.160 (−0.434,0.177)	−0.177 (−0.446,0.160)	−0.239 (−0.506,0.094)	−0.259 (−0.507,0.062)	−0.276 (−0.539,0.055)	−0.299 (−0.563,0.037)	−0.245 (−0.511,0.089)	−0.273 (−0.523,0.049)	−0.189 (−0.626,0.249)	−0.319 (−0.754,0.139)	−0.058 (−0.477,0.360)	

Table S25 shows difference between the relationship variances for each pair of dyad types in the same way as Tables S20, S22, and S23 for the fixed intercepts, autoregressive effects, and cross-lagged effects respectively. The shading is that shown in Table S19, and is again only applied to differences where the credible interval excludes 0.

There are fewer significant differences than for the fixed intercepts, but rather more than for the other paramters whose differences we have examined thus far. The pattern is of some particularly large and some particularly small variances, with each variance being either significantly larger than at least one other and indistinguishable from the rest, or significantly smaller than at least one other and indistinguishable from the rest. The variances for fathers acting towards mothers, younger children acting towards fathers, older children acting towards fathers, and younger children acting towards older children are each significantly smaller than five other variances; the variance for mothers acting towards fathers is significantly smaller than three other variances; and the variances for fathers acting towards older children and older children acting towards younger children are each significantly smaller than one other variance. The variance for mothers acting towards younger children is significantly larger than seven other variances; the variances for younger children acting towards mothers and mothers acting towards older children are each significantly larger than five other variances; and the variances for older children acting towards mothers and fathers acting towards younger children are each significantly larger than four other variances.

The significant differences are generally larger than those between autoregressive effects, cross-lagged effects, or actor variances, partner variances and generalised reciprocities, though generally smaller than those between fixed intercepts. The largest point estimate is 0.355, the amount by which the variance for mothers acting towards younger children is larger than the variance for younger children acting towards older children, while the smallest is 0.174, the amount by which the variance for fathers acting towards younger children is larger than the variance for fathers acting towards mothers.

Table S25: Estimated differences between dyad type effects from Ordinal Dynamic Social Relations model

	M▸F	F▸M	M▸S_1	S_1▸M	M▸S_2	S_2▸M	F▸S_1	S_1▸F	F▸S_2	S_2▸F	S_1▸S_2	S_2▸S_1
M▸F												
F▸M	0.035 (−0.078,0.155)											
M▸S_1	−0.294 (−0.494,−0.106)	−0.329 (−0.521,−0.156)										
S_1▸M	−0.193 (−0.370,−0.025)	−0.228 (−0.395,−0.073)	0.101 (−0.104,0.308)									
M▸S_2	−0.229 (−0.417,−0.052)	−0.264 (−0.444,−0.099)	0.065 (−0.158,0.288)	−0.036 (−0.252,0.175)								
S_2▸M	−0.158 (−0.326,0.002)	−0.193 (−0.351,−0.044)	0.136 (−0.073,0.350)	0.035 (−0.161,0.236)	0.071 (−0.124,0.272)							
F▸S_1	−0.139 (−0.334,0.033)	−0.174 (−0.360,−0.011)	0.155 (−0.068,0.377)	0.054 (−0.160,0.257)	0.090 (−0.133,0.308)	0.019 (−0.197,0.221)						
S_1▸F	0.038 (−0.079,0.163)	0.003 (−0.103,0.109)	0.331 (0.158,0.523)	0.230 (0.081,0.399)	0.266 (0.102,0.447)	0.195 (0.048,0.356)	0.176 (0.021,0.360)					
F▸S_2	−0.061 (−0.223,0.094)	−0.096 (−0.254,0.044)	0.233 (0.025,0.444)	0.132 (−0.061,0.322)	0.168 (−0.028,0.371)	0.097 (−0.089,0.278)	0.078 (−0.118,0.284)	−0.098 (−0.256,0.037)				
S_2▸F	0.042 (−0.066,0.165)	0.008 (−0.091,0.110)	0.336 (0.167,0.527)	0.235 (0.089,0.401)	0.271 (0.112,0.451)	0.200 (0.057,0.358)	0.181 (0.027,0.367)	0.005 (−0.093,0.106)	0.103 (−0.024,0.257)			
S_1▸S_2	0.061 (−0.040,0.179)	0.026 (−0.063,0.124)	0.355 (0.192,0.542)	0.254 (0.111,0.417)	0.290 (0.134,0.466)	0.219 (0.082,0.373)	0.200 (0.050,0.382)	0.023 (−0.064,0.120)	0.122 (−0.004,0.274)	0.019 (−0.068,0.109)		
S_2▸S_1	−0.044 (−0.191,0.100)	−0.079 (−0.219,0.047)	0.250 (0.053,0.457)	0.148 (−0.027,0.334)	0.185 (−0.005,0.380)	0.113 (−0.059,0.291)	0.094 (−0.089,0.296)	−0.082 (−0.221,0.046)	0.017 (−0.150,0.188)	−0.087 (−0.226,0.038)	−0.105 (−0.228,0.000)	

Table S26: Estimated differences between dyadic reciprocities from Ordinal Dynamic Social Relations model

	M&F	M&S_1	M&S_2	F&S_1	F&S_2	S_1&S_2
M&F						
M&S_1	−0.329 (−0.810,0.132)					
M&S_2	−0.107 (−0.612,0.376)	0.222 (−0.125,0.573)				
F&S_1	0.124 (−0.468,0.702)	0.453 (−0.006,0.913)	0.231 (−0.252,0.722)			
F&S_2	0.022 (−0.554,0.591)	0.352 (−0.090,0.825)	0.130 (−0.332,0.620)	−0.101 (−0.664,0.476)		
S_1&S_2	−0.148 (−0.681,0.395)	0.181 (−0.225,0.629)	−0.041 (−0.464,0.427)	−0.272 (−0.808,0.287)	−0.170 (−0.708,0.380)	

Table S26 shows the differences between the dyadic reciprocities for each pair of undirected dyad types, in a similar way to Tables S20, S22, and S23 for the fixed intercepts, autoregressive effects, and cross-lagged effects respectively. The cells would be shaded if any of the 95% credible intervals excluded 0, but they do not, so we do not provide a key to shading colours for this table.

None of the dyadic reciprocities is distinguishable from any other dyadic reciprocity. This is due not to the point estimates for the differences being small – they range from 0.041, for the amount by which the reciprocity between younger and older siblings is estimated to be larger than the reciprocity for mothers and older siblings, to 0.453, for the amount by which the reciprocity for mothers and younger siblings is estimated to be larger than the reciprocity for fathers and younger siblings – but to the 95% credible intervals being extremely wide – even the smallest is 0.698 wide, which on a correlation scale is huge, and six of them are wider than 1. This reflects the wide credible intervals of the estimates of the dyadic reciprocities, whose widths range from 0.452 for mothers and younger children, the sole significant dyadic reciprocity, to 0.807 for fathers and younger children.

Table S27 shows the differences between each dyadic reciprocity (in the columns) and each cross-lagged effect (in the rows). It makes sense to compare these, since they both capture the extent to which an actor's behaviour is influenced by a specific partner's behaviour towards them – at that same snapshot for the dyadic reciprocities and at the previous snapshot for the cross-lagged effects – and are on the same scale. As before, the differences are the quantity in the row subtracted from the quantity in the column. The shading used in cells showing a significant difference is that given in Table S19.

The dyadic reciprocity for mothers and younger children (which was the only dyadic reciprocity we found to be significant) is significantly larger than the cross-lagged effect for every dyad type, including mothers acting towards younger children (indeed the point estimate for this dyad type is the largest of any difference between a cross-lagged effect and this dyadic reciprocity) and younger children acting towards mothers, suggesting for mothers and younger children the present moment may be more important than the immediate past in terms of the actor being influenced by the partner's behaviour. In particular, this dyadic reciprocity is significantly larger than the cross-lagged effect for younger children acting towards older children, which is the only one we found to be significant. However, other than the dyadic reciprocity for mothers and younger children being significantly larger than the cross-lagged effect for mothers acting towards younger children, there are no other significant differences between a dyadic reciprocity and a cross-lagged effect.

Table S28 shows the same thing as Table S27, except instead of taking the difference between the numbers we find at each iteration in the chains for the dyadic reciprocities and cross-lagged effects, we first take the absolute value of these numbers and then calculate the differences. We are thus looking at differences between the cross-lagged effects and dyadic reciprocities in the strength of the effect, regardless of whether it is in the same direction in both cases, and a positive value means that the dyadic reciprocity in question is stronger than the cross-lagged effect in

Table S27: Estimated differences between dyadic reciprocities and cross-lags from Ordinal Dynamic Social Relations model

	M&F	M&S_1	M&S_2	F&S_1	F&S_2	S_1&S_2
M▸F	0.197<br>(−0.248,0.588)	0.527<br>(0.264,0.753)	0.304<br>(−0.002,0.574)	0.073<br>(−0.352,0.481)	0.175<br>(−0.252,0.555)	0.345<br>(−0.056,0.672)
F▸M	0.166<br>(−0.276,0.555)	0.495<br>(0.235,0.719)	0.273<br>(−0.029,0.541)	0.042<br>(−0.381,0.447)	0.144<br>(−0.283,0.521)	0.314<br>(−0.091,0.641)
M▸S_1	0.227<br>(−0.207,0.607)	0.556<br>(0.290,0.789)	0.334<br>(0.036,0.604)	0.103<br>(−0.319,0.507)	0.205<br>(−0.220,0.581)	0.375<br>(−0.025,0.702)
S_1▸M	0.183<br>(−0.248,0.562)	0.512<br>(0.256,0.731)	0.290<br>(−0.005,0.549)	0.059<br>(−0.360,0.459)	0.160<br>(−0.259,0.532)	0.331<br>(−0.068,0.651)
M▸S_2	0.117<br>(−0.312,0.499)	0.447<br>(0.190,0.667)	0.225<br>(−0.082,0.499)	−0.006<br>(−0.424,0.396)	0.095<br>(−0.328,0.471)	0.265<br>(−0.136,0.587)
S_2▸M	0.128<br>(−0.304,0.509)	0.457<br>(0.206,0.675)	0.235<br>(−0.065,0.502)	0.004<br>(−0.415,0.405)	0.106<br>(−0.315,0.475)	0.276<br>(−0.123,0.596)
F▸S_1	0.157<br>(−0.277,0.546)	0.487<br>(0.225,0.716)	0.264<br>(−0.039,0.537)	0.033<br>(−0.404,0.457)	0.135<br>(−0.294,0.513)	0.305<br>(−0.105,0.633)
S_1▸F	0.173<br>(−0.260,0.556)	0.502<br>(0.246,0.721)	0.280<br>(−0.018,0.547)	0.049<br>(−0.373,0.460)	0.150<br>(−0.275,0.526)	0.321<br>(−0.082,0.643)
F▸S_2	0.190<br>(−0.245,0.580)	0.519<br>(0.255,0.750)	0.297<br>(−0.007,0.569)	0.066<br>(−0.359,0.474)	0.167<br>(−0.271,0.560)	0.338<br>(−0.066,0.671)
S_2▸F	0.171<br>(−0.266,0.554)	0.501<br>(0.245,0.720)	0.278<br>(−0.017,0.544)	0.047<br>(−0.374,0.449)	0.149<br>(−0.281,0.526)	0.319<br>(−0.083,0.641)
S_1▸S_2	0.075<br>(−0.360,0.458)	0.404<br>(0.144,0.628)	0.182<br>(−0.118,0.451)	−0.049<br>(−0.469,0.355)	0.052<br>(−0.374,0.430)	0.223<br>(−0.191,0.554)
S_2▸S_1	0.145<br>(−0.294,0.533)	0.475<br>(0.209,0.703)	0.253<br>(−0.050,0.522)	0.022<br>(−0.403,0.429)	0.123<br>(−0.304,0.503)	0.293<br>(−0.118,0.630)

Table S28: Estimated absolute differences between dyadic reciprocities and cross-lags from Ordinal Dynamic Social Relations model

	M&F	M&S_1	M&S_2	F&S_1	F&S_2	S_1&S_2
M▸F	0.183<br>(−0.044,0.511)	0.462<br>(0.206,0.677)	0.243<br>(−0.013,0.501)	0.134<br>(−0.055,0.431)	0.168<br>(−0.049,0.483)	0.290<br>(−0.018,0.599)
F▸M	0.189<br>(−0.038,0.518)	0.468<br>(0.216,0.680)	0.249<br>(−0.004,0.507)	0.141<br>(−0.045,0.434)	0.174<br>(−0.039,0.487)	0.296<br>(−0.010,0.605)
M▸S_1	0.167<br>(−0.069,0.498)	0.445<br>(0.197,0.657)	0.227<br>(−0.033,0.490)	0.118<br>(−0.078,0.415)	0.151<br>(−0.071,0.465)	0.274<br>(−0.038,0.584)
S_1▸M	0.200<br>(−0.019,0.526)	0.479<br>(0.231,0.687)	0.260<br>(0.007,0.514)	0.152<br>(−0.025,0.444)	0.185<br>(−0.020,0.494)	0.307<br>(0.002,0.614)
M▸S_2	0.166<br>(−0.066,0.496)	0.445<br>(0.190,0.662)	0.226<br>(−0.039,0.494)	0.118<br>(−0.074,0.414)	0.151<br>(−0.068,0.470)	0.273<br>(−0.038,0.582)
S_2▸M	0.177<br>(−0.050,0.506)	0.456<br>(0.205,0.670)	0.237<br>(−0.022,0.497)	0.128<br>(−0.059,0.421)	0.162<br>(−0.052,0.474)	0.284<br>(−0.024,0.592)
F▸S_1	0.183<br>(−0.047,0.510)	0.461<br>(0.209,0.677)	0.243<br>(−0.013,0.502)	0.134<br>(−0.059,0.430)	0.167<br>(−0.051,0.483)	0.290<br>(−0.021,0.598)
S_1▸F	0.194<br>(−0.027,0.518)	0.473<br>(0.222,0.684)	0.254<br>(0.001,0.510)	0.146<br>(−0.036,0.436)	0.179<br>(−0.030,0.489)	0.301<br>(−0.005,0.606)
F▸S_2	0.182<br>(−0.049,0.512)	0.461<br>(0.208,0.675)	0.242<br>(−0.012,0.502)	0.134<br>(−0.057,0.427)	0.167<br>(−0.049,0.477)	0.289<br>(−0.020,0.598)
S_2▸F	0.196<br>(−0.025,0.521)	0.474<br>(0.223,0.684)	0.255<br>(0.002,0.509)	0.147<br>(−0.033,0.438)	0.180<br>(−0.029,0.492)	0.302<br>(−0.004,0.609)
S_1▸S_2	0.125<br>(−0.115,0.458)	0.404<br>(0.144,0.627)	0.185<br>(−0.076,0.449)	0.077<br>(−0.122,0.375)	0.110<br>(−0.116,0.430)	0.232<br>(−0.091,0.554)
S_2▸S_1	0.179<br>(−0.054,0.508)	0.458<br>(0.200,0.675)	0.239<br>(−0.017,0.498)	0.131<br>(−0.063,0.424)	0.164<br>(−0.058,0.478)	0.286<br>(−0.028,0.598)

Table S29: Estimated differences between innovation correlations and cross-lags from Ordinal Dynamic Social Relations model

	M&F	M&S_1	M&S_2	F&S_1	F&S_2	S_1&S_2
M▸F	−0.116	0.068	0.313	0.422	0.265	0.182
	(−0.428,0.214)	(−0.119,0.251)	(0.114,0.502)	(0.206,0.625)	(0.026,0.489)	(−0.080,0.426)
F▸M	−0.147	0.037	0.282	0.391	0.233	0.151
	(−0.459,0.187)	(−0.146,0.219)	(0.083,0.468)	(0.175,0.590)	(−0.004,0.459)	(−0.107,0.394)
M▸S_1	−0.086	0.098	0.343	0.452	0.294	0.212
	(−0.392,0.244)	(−0.086,0.282)	(0.148,0.528)	(0.238,0.650)	(0.057,0.516)	(−0.045,0.453)
S_1▸M	−0.130	0.054	0.299	0.408	0.250	0.168
	(−0.430,0.195)	(−0.118,0.224)	(0.111,0.473)	(0.201,0.596)	(0.019,0.463)	(−0.084,0.403)
M▸S_2	−0.195	−0.012	0.233	0.342	0.185	0.102
	(−0.500,0.135)	(−0.188,0.161)	(0.038,0.414)	(0.132,0.536)	(−0.051,0.402)	(−0.152,0.341)
S_2▸M	−0.185	−0.001	0.244	0.353	0.195	0.113
	(−0.488,0.141)	(−0.174,0.169)	(0.054,0.422)	(0.145,0.545)	(−0.037,0.411)	(−0.139,0.348)
F▸S_1	−0.156	0.028	0.273	0.382	0.225	0.142
	(−0.470,0.181)	(−0.162,0.215)	(0.071,0.464)	(0.161,0.591)	(−0.018,0.453)	(−0.123,0.392)
S_1▸F	−0.140	0.044	0.289	0.397	0.240	0.157
	(−0.446,0.189)	(−0.134,0.219)	(0.095,0.469)	(0.183,0.597)	(0.006,0.456)	(−0.097,0.397)
F▸S_2	−0.123	0.061	0.306	0.415	0.257	0.175
	(−0.435,0.215)	(−0.129,0.251)	(0.102,0.500)	(0.195,0.623)	(0.016,0.488)	(−0.087,0.424)
S_2▸F	−0.142	0.042	0.287	0.396	0.239	0.156
	(−0.447,0.189)	(−0.135,0.218)	(0.093,0.467)	(0.185,0.591)	(0.007,0.451)	(−0.097,0.395)
S_1▸S_2	−0.238	−0.054	0.191	0.300	0.142	0.059
	(−0.547,0.095)	(−0.235,0.125)	(−0.007,0.376)	(0.086,0.499)	(−0.096,0.364)	(−0.196,0.299)
S_2▸S_1	−0.167	0.016	0.261	0.370	0.213	0.130
	(−0.477,0.168)	(−0.172,0.202)	(0.059,0.453)	(0.149,0.577)	(−0.029,0.440)	(−0.131,0.378)

question, whereas in Table S27 it instead means the dyadic reciprocity is larger, which would actually mean weaker in cases where both are negative. Again, the shading for the significant differences is that shown in Table S19.

The results are not much different than in Table S27. We find again that the dyadic reciprocity for mothers and younger children is significantly stronger than the cross-lagged effect for every dyad type (although the differences are slightly reduced in magnitude), and additionally find that the dyadic reciprocity for mothers and older children is significantly stronger than the cross-lagged effect for three dyad types: younger children acting towards mothers younger children acting towards fathers and older children acting towards fathers, although not significantly stronger than the cross-lagged effect for the dyad type we had previously found it to be significantly larger than, mothers acting towards younger children, and that the dyadic reciprocity for younger and older siblings is significantly stronger than the cross-lagged effect for younger children acting towards mothers.

Whether we look at values as they are or at absolute values, the significant differences are generally larger than for any other differences we have looked at, except those between fixed intercepts. The non-significant differences are in many cases fairly large too, but have wide credible intervals. The lower ends of the credible intervals for differences from the dyadic reciprocity for mothers and younger children are in all cases (and whether we look at original or absolute values) quite far from zero, but the lower ends for the other differences we find to be significant in both tables are much closer (very close, in the case of Table S28

Table S29 shows the differences between each innovation correlation for snapshots after the first (in the columns) each cross-lag parameter (in the rows), and Table S30 shows the same thing for the innovation correlations at the first snapshot. Tables S31 and S32 show the equivalent differences between absolute values. (In each case, we subtract the cross-lag parameter from the innovation correlation.) Again, the shading for cells showing significant differences is that shown in Table S19.

Table S30: Estimated differences between innovation correlations at the first snapshot and cross-lags from Ordinal Dynamic Social Relations model

	M&F	M&S_1	M&S_2	F&S_1	F&S_2	S_1&S_2
M▶F	0.003 (−0.135,0.143)	0.113 (0.005,0.221)	0.128 (0.020,0.236)	0.208 (0.087,0.327)	0.131 (0.005,0.257)	0.350 (0.228,0.471)
F▶M	−0.028 (−0.167,0.111)	0.082 (−0.021,0.185)	0.097 (−0.007,0.201)	0.177 (0.061,0.293)	0.100 (−0.021,0.222)	0.319 (0.201,0.438)
M▶S_1	0.033 (−0.105,0.172)	0.143 (0.046,0.240)	0.158 (0.057,0.259)	0.238 (0.126,0.350)	0.161 (0.041,0.280)	0.380 (0.265,0.493)
S_1▶M	−0.011 (−0.137,0.115)	0.099 (0.017,0.179)	0.114 (0.030,0.198)	0.194 (0.096,0.289)	0.117 (0.012,0.221)	0.336 (0.235,0.435)
M▶S_2	−0.077 (−0.210,0.058)	0.033 (−0.059,0.127)	0.048 (−0.043,0.140)	0.128 (0.022,0.234)	0.051 (−0.062,0.166)	0.270 (0.159,0.377)
S_2▶M	−0.066 (−0.194,0.063)	0.044 (−0.042,0.130)	0.059 (−0.027,0.144)	0.139 (0.038,0.239)	0.062 (−0.045,0.168)	0.281 (0.178,0.383)
F▶S_1	−0.037 (−0.186,0.114)	0.073 (−0.042,0.187)	0.088 (−0.028,0.203)	0.168 (0.048,0.289)	0.091 (−0.040,0.222)	0.310 (0.183,0.437)
S_1▶F	−0.022 (−0.157,0.116)	0.089 (−0.007,0.186)	0.104 (0.007,0.199)	0.183 (0.076,0.289)	0.107 (−0.009,0.221)	0.326 (0.213,0.435)
F▶S_2	−0.004 (−0.153,0.145)	0.106 (−0.010,0.220)	0.121 (0.006,0.235)	0.201 (0.075,0.325)	0.124 (−0.002,0.250)	0.343 (0.215,0.471)
S_2▶F	−0.023 (−0.156,0.111)	0.087 (−0.005,0.178)	0.102 (0.007,0.196)	0.182 (0.076,0.288)	0.105 (−0.004,0.217)	0.324 (0.214,0.432)
S_1▶S_2	−0.119 (−0.261,0.021)	−0.009 (−0.110,0.091)	0.006 (−0.097,0.109)	0.085 (−0.028,0.199)	0.009 (−0.111,0.129)	0.228 (0.114,0.341)
S_2▶S_1	−0.049 (−0.196,0.099)	0.061 (−0.051,0.174)	0.076 (−0.036,0.191)	0.156 (0.031,0.280)	0.080 (−0.050,0.210)	0.298 (0.178,0.419)

Table S31: Estimated absolute differences between innovation correlations and cross-lags from Ordinal Dynamic Social Relations model

	M&F	M&S_1	M&S_2	F&S_1	F&S_2	S_1&S_2
M▸F	0.133 (−0.054,0.403)	0.034 (−0.075,0.175)	0.248 (0.056,0.421)	0.357 (0.148,0.546)	0.201 (−0.013,0.413)	0.129 (−0.051,0.351)
F▸M	0.139 (−0.042,0.403)	0.040 (−0.061,0.177)	0.254 (0.067,0.427)	0.363 (0.158,0.551)	0.207 (−0.005,0.417)	0.135 (−0.040,0.355)
M▸S_1	0.117 (−0.076,0.386)	0.018 (−0.094,0.159)	0.232 (0.037,0.408)	0.341 (0.130,0.533)	0.185 (−0.032,0.400)	0.113 (−0.072,0.338)
S_1▸M	0.151 (−0.024,0.413)	0.051 (−0.037,0.184)	0.266 (0.082,0.433)	0.374 (0.172,0.560)	0.218 (0.008,0.425)	0.146 (−0.020,0.364)
M▸S_2	0.117 (−0.072,0.385)	0.017 (−0.091,0.159)	0.232 (0.038,0.408)	0.340 (0.131,0.531)	0.184 (−0.033,0.398)	0.112 (−0.069,0.338)
S_2▸M	0.127 (−0.057,0.394)	0.028 (−0.074,0.168)	0.242 (0.054,0.418)	0.351 (0.145,0.540)	0.195 (−0.019,0.407)	0.123 (−0.052,0.345)
F▸S_1	0.133 (−0.055,0.401)	0.034 (−0.078,0.175)	0.248 (0.057,0.422)	0.357 (0.146,0.548)	0.201 (−0.014,0.413)	0.129 (−0.051,0.351)
S_1▸F	0.144 (−0.033,0.410)	0.045 (−0.051,0.181)	0.259 (0.073,0.430)	0.368 (0.164,0.555)	0.212 (0.000,0.420)	0.140 (−0.030,0.359)
F▸S_2	0.132 (−0.057,0.401)	0.033 (−0.079,0.172)	0.247 (0.055,0.421)	0.356 (0.147,0.548)	0.200 (−0.014,0.413)	0.128 (−0.053,0.350)
S_2▸F	0.146 (−0.031,0.410)	0.047 (−0.047,0.181)	0.261 (0.075,0.430)	0.370 (0.166,0.556)	0.214 (0.004,0.422)	0.142 (−0.029,0.360)
S_1▸S_2	0.075 (−0.121,0.347)	−0.024 (−0.143,0.127)	0.190 (−0.007,0.375)	0.299 (0.086,0.497)	0.143 (−0.078,0.363)	0.071 (−0.116,0.299)
S_2▸S_1	0.129 (−0.061,0.398)	0.030 (−0.084,0.173)	0.244 (0.052,0.418)	0.353 (0.142,0.545)	0.197 (−0.018,0.410)	0.125 (−0.056,0.349)

Table S32: Estimated absolute differences between innovation correlations at the first snapshot and cross-lags from Ordinal Dynamic Social Relations model

	M&F	M&S_1	M&S_2	F&S_1	F&S_2	S_1&S_2
M▸F	0.008<br>(−0.084,0.107)	0.048<br>(−0.044,0.126)	0.063<br>(−0.030,0.142)	0.143<br>(0.039,0.236)	0.067<br>(−0.041,0.168)	0.285<br>(0.177,0.382)
F▸M	0.014<br>(−0.070,0.111)	0.054<br>(−0.029,0.129)	0.069<br>(−0.016,0.145)	0.149<br>(0.050,0.239)	0.073<br>(−0.030,0.170)	0.291<br>(0.190,0.384)
M▸S_1	−0.008<br>(−0.105,0.098)	0.032<br>(−0.067,0.120)	0.047<br>(−0.051,0.134)	0.127<br>(0.017,0.229)	0.050<br>(−0.064,0.158)	0.269<br>(0.156,0.371)
S_1▸M	0.025<br>(−0.043,0.119)	0.066<br>(−0.006,0.134)	0.080<br>(0.007,0.148)	0.160<br>(0.071,0.244)	0.084<br>(−0.010,0.177)	0.303<br>(0.210,0.389)
M▸S_2	−0.009<br>(−0.100,0.096)	0.032<br>(−0.059,0.117)	0.046<br>(−0.043,0.131)	0.126<br>(0.022,0.225)	0.050<br>(−0.061,0.157)	0.268<br>(0.159,0.369)
S_2▸M	0.002<br>(−0.081,0.102)	0.042<br>(−0.042,0.121)	0.057<br>(−0.027,0.135)	0.137<br>(0.038,0.231)	0.061<br>(−0.042,0.162)	0.279<br>(0.178,0.375)
F▸S_1	0.008<br>(−0.087,0.108)	0.048<br>(−0.046,0.126)	0.063<br>(−0.032,0.142)	0.143<br>(0.039,0.235)	0.066<br>(−0.045,0.168)	0.285<br>(0.176,0.381)
S_1▸F	0.019<br>(−0.058,0.114)	0.059<br>(−0.020,0.130)	0.074<br>(−0.005,0.146)	0.154<br>(0.060,0.241)	0.078<br>(−0.022,0.174)	0.296<br>(0.198,0.387)
F▸S_2	0.007<br>(−0.087,0.109)	0.047<br>(−0.047,0.126)	0.062<br>(−0.033,0.141)	0.142<br>(0.036,0.236)	0.066<br>(−0.046,0.168)	0.284<br>(0.176,0.382)
S_2▸F	0.020<br>(−0.055,0.114)	0.061<br>(−0.016,0.131)	0.076<br>(−0.002,0.146)	0.155<br>(0.063,0.242)	0.079<br>(−0.017,0.174)	0.298<br>(0.201,0.387)
S_1▸S_2	−0.050<br>(−0.150,0.064)	−0.010<br>(−0.110,0.089)	0.005<br>(−0.097,0.106)	0.085<br>(−0.028,0.197)	0.009<br>(−0.109,0.127)	0.227<br>(0.114,0.338)
S_2▸S_1	0.004<br>(−0.093,0.106)	0.044<br>(−0.053,0.125)	0.059<br>(−0.038,0.141)	0.139<br>(0.029,0.235)	0.063<br>(−0.051,0.166)	0.281<br>(0.175,0.378)

Table S33: Estimated differences between dyadic reciprocities (columns) and innovation correlations (rows) from Ordinal Dynamic Social Relations model

	M&F	M&S_1	M&S_2	F&S_1	F&S_2	S_1&S_2
M&F	0.313<br>(−0.222,0.799)	0.642<br>(0.246,1.011)	0.420<br>(−0.002,0.815)	0.189<br>(−0.339,0.694)	0.290<br>(−0.233,0.774)	0.461<br>(−0.044,0.901)
M&S_1	0.129<br>(−0.323,0.537)	0.458<br>(0.160,0.729)	0.236<br>(−0.095,0.538)	0.005<br>(−0.438,0.434)	0.107<br>(−0.338,0.506)	0.277<br>(−0.148,0.635)
M&S_2	−0.116<br>(−0.572,0.302)	0.213<br>(−0.081,0.488)	−0.009<br>(−0.345,0.306)	−0.240<br>(−0.684,0.193)	−0.138<br>(−0.586,0.272)	0.032<br>(−0.395,0.397)
F&S_1	−0.225<br>(−0.688,0.200)	0.104<br>(−0.199,0.396)	−0.118<br>(−0.462,0.210)	−0.349<br>(−0.806,0.097)	−0.247<br>(−0.708,0.171)	−0.077<br>(−0.511,0.297)
F&S_2	−0.067<br>(−0.537,0.369)	0.262<br>(−0.058,0.568)	0.040<br>(−0.317,0.382)	−0.191<br>(−0.658,0.259)	−0.090<br>(−0.566,0.354)	0.081<br>(−0.364,0.474)
S_1&S_2	0.015<br>(−0.470,0.464)	0.345<br>(0.009,0.668)	0.122<br>(−0.246,0.472)	−0.109<br>(−0.588,0.361)	−0.007<br>(−0.484,0.442)	0.163<br>(−0.302,0.579)

For innovation correlations for snapshots after the first, whether we look at differences or differences in absolute values, the correlations for mothers and older children and for fathers and younger children are larger than all the cross-lag parameters, except that the innovation correlation for mothers and older children is not larger than the cross-lag parameter for younger siblings acting towards older siblings. The innovation correlation for fathers and older children is also larger than the cross-lag parameters for some dyad types: when we look at differences in absolute values, those for younger children acting towards mothers, and for both younger and older children acting towards fathers, and when we look at differences in signed values, the same and additionally those for mothers acting towards fathers, mothers acting towards younger children, and fathers acting towards older children. We cannot tell which is larger out of the innovation correlation and the cross-lag parameter for any other combination of an undirected and a directed dyad type. The significant differences are large, ranging from around 0.23 to around 0.45 for the differences in signed values and from around 0.21 to around 0.37 for the differences in absolute values.

For innovation correlations at the first snapshot, we see somewhat different patterns. Whether we look at the differences in absolute or signed values, the innovation correlations for fathers and younger children and for younger and older siblings are larger than the cross-lag parameter for every dyad type, except that the innovation correlation for fathers and younger children is not larger than the cross-lag parameter for younger siblings acting towards older siblings. For the differences in absolute values, additionally the innovation correlation for mothers and older children is larger than the cross-lag parameter for younger children acting towards mothers (although the difference is relatively small). For the differences in signed values, the cross-lag parameters for mothers acting towards fathers, mothers acting towards younger children, and younger children acting towards mothers are all smaller than every innovation correlation except that for mothers and fathers, and the innovation correlation for mothers and older children is also larger than the cross-lag parameters for younger children acting towards fathers, fathers acting towards older children, and older children acting towards fathers. Again, we cannot tell which out of the innovation correlation and the cross-lag parameter is larger in any other case. The significant differences are generally smaller than for the innovation correlations for snapshots after the first, ranging from around 0.10 to around 0.38 for the differences in signed values and from around 0.08 to around 0.30 for the differences in absolute values.

Table S33 shows the differences in signed values between the dyadic reciprocities and the innovation correlations for innovations after the first snapshot, Table S34 shows the differences in absolute values, and Tables S35 and S36 show the same for innovation correlations at the first snapshot. In each case, the dyadic reciprocities are in the columns, the innovation correlations are in the rows, and the value shown is calculated by subtracting the innovation correlation from the dyadic reciprocity. Again, the shading applied to cells showing significant differences is that shown in Table S19.

Whether we look at differences in signed values or in absolute values, and at the innovation correlations for innovations after the first snapshot or the innovation correlations at the snapshot, the only dyadic reciprocity that is larger

Table S34: Estimated absolute differences between dyadic reciprocities (columns) and innovation correlations (rows) from Ordinal Dynamic Social Relations model

	M&F	M&S_1	M&S_2	F&S_1	F&S_2	S_1&S_2
M&F	0.050<br>(−0.312,0.433)	0.328<br>(−0.025,0.618)	0.110<br>(−0.250,0.436)	0.001<br>(−0.331,0.354)	0.034<br>(−0.319,0.401)	0.157<br>(−0.254,0.532)
M&S_1	0.149<br>(−0.112,0.487)	0.427<br>(0.151,0.660)	0.209<br>(−0.065,0.481)	0.100<br>(−0.128,0.406)	0.134<br>(−0.118,0.459)	0.256<br>(−0.072,0.577)
M&S_2	−0.065<br>(−0.360,0.303)	0.213<br>(−0.081,0.488)	−0.005<br>(−0.310,0.306)	−0.114<br>(−0.373,0.218)	−0.081<br>(−0.363,0.274)	0.042<br>(−0.312,0.396)
F&S_1	−0.174<br>(−0.482,0.201)	0.104<br>(−0.199,0.396)	−0.114<br>(−0.431,0.210)	−0.223<br>(−0.495,0.121)	−0.189<br>(−0.482,0.174)	−0.067<br>(−0.433,0.297)
F&S_2	−0.018<br>(−0.342,0.365)	0.261<br>(−0.058,0.555)	0.042<br>(−0.286,0.372)	−0.067<br>(−0.359,0.283)	−0.033<br>(−0.349,0.350)	0.089<br>(−0.288,0.467)
S_1&S_2	0.054<br>(−0.278,0.428)	0.332<br>(0.008,0.612)	0.114<br>(−0.217,0.428)	0.005<br>(−0.291,0.349)	0.039<br>(−0.280,0.405)	0.161<br>(−0.224,0.533)

Table S35: Estimated differences between dyadic reciprocities (columns) and innovation correlations (rows) at the first snapshot from Ordinal Dynamic Social Relations model

	M&F	M&S_1	M&S_2	F&S_1	F&S_2	S_1&S_2
M&F	0.194<br>(−0.254,0.591)	0.523<br>(0.253,0.763)	0.301<br>(−0.009,0.583)	0.070<br>(−0.358,0.481)	0.172<br>(−0.261,0.558)	0.342<br>(−0.071,0.678)
M&S_1	0.084<br>(−0.349,0.461)	0.413<br>(0.155,0.633)	0.191<br>(−0.103,0.452)	−0.040<br>(−0.456,0.363)	0.062<br>(−0.360,0.433)	0.232<br>(−0.165,0.550)
M&S_2	0.069<br>(−0.364,0.446)	0.398<br>(0.143,0.616)	0.176<br>(−0.124,0.444)	−0.055<br>(−0.471,0.345)	0.047<br>(−0.375,0.418)	0.217<br>(−0.182,0.536)
F&S_1	−0.011<br>(−0.444,0.370)	0.319<br>(0.060,0.542)	0.096<br>(−0.205,0.363)	−0.135<br>(−0.561,0.274)	−0.033<br>(−0.458,0.341)	0.137<br>(−0.265,0.462)
F&S_2	0.066<br>(−0.370,0.453)	0.395<br>(0.135,0.621)	0.173<br>(−0.127,0.445)	−0.058<br>(−0.480,0.348)	0.043<br>(−0.391,0.428)	0.214<br>(−0.188,0.542)
S_1&S_2	−0.153<br>(−0.587,0.230)	0.176<br>(−0.085,0.404)	−0.046<br>(−0.347,0.221)	−0.277<br>(−0.701,0.129)	−0.175<br>(−0.602,0.200)	−0.005<br>(−0.415,0.325)

Table S36: Estimated absolute differences between dyadic reciprocities (columns) and innovation correlations at the first snapshot (rows) from Ordinal Dynamic Social Relations model

	M&F	M&S_1	M&S_2	F&S_1	F&S_2	S_1&S_2
M&F	0.175<br>(−0.061,0.505)	0.454<br>(0.196,0.672)	0.235<br>(−0.026,0.494)	0.127<br>(−0.073,0.421)	0.160<br>(−0.065,0.475)	0.282<br>(−0.030,0.595)
M&S_1	0.135<br>(−0.094,0.461)	0.413<br>(0.155,0.633)	0.195<br>(−0.060,0.452)	0.086<br>(−0.102,0.382)	0.119<br>(−0.097,0.435)	0.242<br>(−0.068,0.550)
M&S_2	0.120<br>(−0.109,0.446)	0.398<br>(0.143,0.616)	0.180<br>(−0.082,0.444)	0.071<br>(−0.117,0.367)	0.104<br>(−0.112,0.419)	0.227<br>(−0.084,0.536)
F&S_1	0.040<br>(−0.199,0.371)	0.319<br>(0.060,0.542)	0.100<br>(−0.161,0.363)	−0.008<br>(−0.207,0.296)	0.025<br>(−0.202,0.344)	0.147<br>(−0.172,0.462)
F&S_2	0.117<br>(−0.128,0.452)	0.395<br>(0.135,0.620)	0.176<br>(−0.089,0.444)	0.068<br>(−0.135,0.371)	0.101<br>(−0.131,0.429)	0.223<br>(−0.093,0.542)
S_1&S_2	−0.102<br>(−0.341,0.231)	0.176<br>(−0.085,0.404)	−0.042<br>(−0.306,0.221)	−0.151<br>(−0.351,0.150)	−0.118<br>(−0.345,0.202)	0.005<br>(−0.314,0.325)

than any innovation correlation is that for mothers and younger children. (This was the only dyadic reciprocity found to be significant.) At the first snapshot, it is larger than the innovation correlations for all undirected dyad types except younger and older siblings, whether we look at difference in signed or absolute values. For snapshots after the first, it is larger than the innovation correlations for mothers and younger children and for younger and older siblings whichever kind of differences we look at, and for differences in signed values it is also larger than the innovation correlation for mothers and fathers. For all other combinations of innovation correlation at one undirected dyad type and dyadic reciprocity at the same or another type, we cannot say which is larger. The significant differences are again quite large, ranging from around 0.31 to around 0.45 (differences in absolute values) or 0.52 (differences in signed values) for innovation correlations at the first snapshot and around 0.33 or 0.35 to around 0.43 (differences in absolute values) or 0.64 (differences in signed values) for innovation correlations for snapshots after the first.

We therefore have evidence that some of the dyadic reciprocities are larger (both stronger and more positive) than some of the innovation correlations and than some of the cross-lag parameters, and that some of the innovation correlations are larger (again both stronger and more positive) than some of the cross-lag parameters, but not enough evidence to say that in general the dyadic reciprocities are larger than the innovation correlations which are in turn larger than the cross-lag parameters (which would imply that the most important way in which the members of a dyad influence each other is through their general behaviour over time, followed by their behaviour at the present moment, with their behaviour in the immediate past the least important). The only cases where we find a significant difference (whether in signed or absolute values) between parameters for the same undirected dyad type, or an undirected dyad type and one of the directed dyad types involving its members, are that the dyadic reciprocity for mothers and younger children is larger than the cross-lag parameters for both mothers acting towards younger children and younger children acting towards mothers; that the innovation correlations for snapshots after the first are larger than the cross-lag parameters for both relevant directed dyads for mothers and older children, fathers and younger children, and fathers and older children; that the innovation correlations at the first snapshot are larger than the cross-lag parameters for both relevant directed dyads for mothers and younger children, fathers and younger children, and younger and older siblings; and that the dyadic reciprocity is larger than the innovation correlation for mothers and younger children.

S5.4 Adding a time trend

Table S37: Results of Ordinal DSRM, and Ordinal DSRM with time trends

	Ordinal DSRM			Ordinal DSRM with time trends		
	Mean	Quantiles		Mean	Quantiles	
		2.5%	97.5%		2.5%	97.5%
τ_1	0.000	0.000	0.000	0.000	0.000	0.000
τ_2	1.791	1.759	1.812	1.814	1.767	1.853
τ_3	4.386	4.322	4.443	4.424	4.366	4.493
μ (M▸F)	2.244	2.115	2.372	2.339	2.178	2.499
μ (F▸M)	2.015	1.886	2.146	2.091	1.925	2.255
μ (M▸S_1)	2.596	2.460	2.735	2.661	2.503	2.819
μ (S_1▸M)	1.139	1.017	1.260	1.300	1.156	1.445
μ (M▸S_2)	2.589	2.460	2.719	2.647	2.496	2.798
μ (S_2▸M)	1.516	1.400	1.632	1.697	1.554	1.842
μ (F▸S_1)	2.774	2.625	2.923	2.856	2.679	3.032
μ (S_1▸F)	1.471	1.352	1.590	1.537	1.387	1.688
μ (F▸S_2)	2.369	2.234	2.505	2.489	2.323	2.657
μ (S_2▸F)	1.531	1.420	1.643	1.575	1.430	1.716
μ (S_1▸S_2)	1.416	1.307	1.525	1.521	1.387	1.656

(cont.)

(cont.)

	Ordinal DSRM			Ordinal DSRM with time trends		
	Mean	Quantiles		Mean	Quantiles	
		2.5%	97.5%		2.5%	97.5%
μ (S$_2$▸S$_1$)	1.648	1.529	1.766	1.881	1.737	2.024
ϕ_1 (M▸F)	0.068	−0.025	0.162	0.063	−0.029	0.157
ϕ_1 (F▸M)	0.202	0.112	0.293	0.199	0.106	0.292
ϕ_1 (M▸S$_1$)	0.095	0.029	0.161	0.093	0.027	0.159
ϕ_1 (S$_1$▸M)	0.224	0.150	0.297	0.210	0.137	0.284
ϕ_1 (M▸S$_2$)	0.102	0.033	0.171	0.101	0.032	0.169
ϕ_1 (S$_2$▸M)	0.237	0.168	0.307	0.225	0.155	0.294
ϕ_1 (F▸S$_1$)	0.085	−0.006	0.175	0.080	−0.011	0.172
ϕ_1 (S$_1$▸F)	0.208	0.121	0.296	0.210	0.124	0.297
ϕ_1 (F▸S$_2$)	0.108	0.023	0.192	0.100	0.015	0.186
ϕ_1 (S$_2$▸F)	0.120	0.030	0.209	0.120	0.032	0.210
ϕ_1 (S$_1$▸S$_2$)	0.143	0.051	0.233	0.143	0.051	0.233
ϕ_1 (S$_2$▸S$_1$)	0.164	0.067	0.262	0.133	0.037	0.231
ϕ_2 (M▸F)	−0.024	−0.113	0.066	−0.030	−0.118	0.060
ϕ_2 (F▸M)	0.007	−0.078	0.092	0.005	−0.080	0.089
ϕ_2 (M▸S$_1$)	−0.054	−0.135	0.027	−0.063	−0.145	0.017
ϕ_2 (S$_1$▸M)	−0.009	−0.065	0.046	−0.011	−0.067	0.044
ϕ_2 (M▸S$_2$)	0.056	−0.014	0.127	0.057	−0.014	0.129
ϕ_2 (S$_2$▸M)	0.045	−0.015	0.106	0.047	−0.013	0.108
ϕ_2 (F▸S$_1$)	0.016	−0.080	0.114	0.013	−0.084	0.112
ϕ_2 (S$_1$▸F)	0.001	−0.073	0.075	−0.001	−0.076	0.075
ϕ_2 (F▸S$_2$)	−0.016	−0.115	0.080	−0.017	−0.115	0.080
ϕ_2 (S$_2$▸F)	0.002	−0.068	0.073	0.002	−0.070	0.073
ϕ_2 (S$_1$▸S$_2$)	0.099	0.015	0.180	0.094	0.011	0.175
ϕ_2 (S$_2$▸S$_1$)	0.028	−0.067	0.123	0.026	−0.067	0.119
α_1 (M▸F)				−0.014	−0.031	0.003
α_1 (F▸M)				−0.011	−0.029	0.007
α_1 (M▸S$_1$)				−0.009	−0.024	0.006
α_1 (S$_1$▸M)				−0.032	−0.048	−0.016
α_1 (M▸S$_2$)				−0.006	−0.020	0.008
α_1 (S$_2$▸M)				−0.033	−0.048	−0.018
α_1 (F▸S$_1$)				−0.012	−0.030	0.007
α_1 (S$_1$▸F)				−0.010	−0.029	0.009
α_1 (F▸S$_2$)				−0.020	−0.037	−0.002
α_1 (S$_2$▸F)				−0.005	−0.022	0.011
α_1 (S$_1$▸S$_2$)				−0.024	−0.044	−0.005
α_1 (S$_2$▸S$_1$)				−0.057	−0.077	−0.037
σ_f^2	0.031	0.001	0.111	0.027	0.001	0.102
σ_a^2 (M)	0.200	0.121	0.294	0.207	0.126	0.305
σ_a^2 (F)	0.183	0.110	0.273	0.191	0.116	0.285
σ_a^2 (S$_1$)	0.121	0.072	0.182	0.125	0.075	0.189
σ_a^2 (S$_2$)	0.101	0.059	0.157	0.104	0.061	0.161
σ_p^2 (M)	0.084	0.049	0.132	0.084	0.049	0.133
σ_p^2 (F)	0.061	0.037	0.094	0.061	0.038	0.094
σ_p^2 (S$_1$)	0.115	0.066	0.180	0.118	0.068	0.185
σ_p^2 (S$_2$)	0.087	0.050	0.136	0.088	0.051	0.137
σ_d^2 (M▸F)	0.157	0.081	0.266	0.160	0.081	0.271

(cont.)

(cont.)

	Ordinal DSRM			Ordinal DSRM with time trends		
	Mean	Quantiles		Mean	Quantiles	
		2.5%	97.5%		2.5%	97.5%
σ_d^2 (F▸M)	0.122	0.063	0.210	0.125	0.065	0.214
σ_d^2 (M▸S$_1$)	0.451	0.300	0.630	0.461	0.307	0.644
σ_d^2 (S$_1$▸M)	0.350	0.223	0.504	0.356	0.227	0.510
σ_d^2 (M▸S$_2$)	0.386	0.243	0.557	0.392	0.249	0.562
σ_d^2 (S$_2$▸M)	0.315	0.193	0.463	0.331	0.205	0.481
σ_d^2 (F▸S$_1$)	0.296	0.161	0.472	0.304	0.166	0.484
σ_d^2 (S$_1$▸F)	0.119	0.063	0.204	0.121	0.062	0.208
σ_d^2 (F▸S$_2$)	0.218	0.110	0.362	0.228	0.117	0.377
σ_d^2 (S$_2$▸F)	0.115	0.062	0.191	0.116	0.062	0.195
σ_d^2 (S$_1$▸S$_2$)	0.096	0.052	0.161	0.096	0.052	0.162
σ_d^2 (S$_2$▸S$_1$)	0.201	0.106	0.327	0.211	0.112	0.336
ρ_{ap} (M)	0.171	−0.188	0.485	0.195	−0.158	0.508
ρ_{ap} (F)	0.041	−0.301	0.374	0.051	−0.295	0.383
ρ_{ap} (S$_1$)	0.302	−0.039	0.580	0.317	−0.018	0.589
ρ_{ap} (S$_2$)	0.360	0.025	0.622	0.362	0.034	0.622
ρ_d (M&F)	0.173	−0.254	0.550	0.178	−0.248	0.549
ρ_d (M&S$_1$)	0.503	0.257	0.709	0.507	0.265	0.712
ρ_d (M&S$_2$)	0.281	−0.009	0.534	0.269	−0.017	0.520
ρ_d (F&S$_1$)	0.050	−0.364	0.443	0.051	−0.368	0.451
ρ_d (F&S$_2$)	0.151	−0.268	0.515	0.155	−0.259	0.525
ρ_d (S$_1$&S$_2$)	0.321	−0.071	0.633	0.315	−0.083	0.634
ρ_{e1} (M&F)	−0.139	−0.436	0.181	−0.143	−0.435	0.165
ρ_{e1} (M&S$_1$)	0.044	−0.119	0.203	0.040	−0.120	0.199
ρ_{e1} (M&S$_2$)	0.289	0.110	0.453	0.288	0.111	0.453
ρ_{e1} (F&S$_1$)	0.398	0.199	0.580	0.392	0.191	0.576
ρ_{e1} (F&S$_2$)	0.241	0.016	0.447	0.239	0.017	0.444
ρ_{e1} (S$_1$&S$_2$)	0.158	−0.086	0.386	0.088	−0.169	0.330
ρ_η (M&F)	−0.021	−0.133	0.093	−0.027	−0.140	0.088
ρ_η (M&S$_1$)	0.089	0.029	0.149	0.085	0.024	0.145
ρ_η (M&S$_2$)	0.104	0.042	0.165	0.104	0.043	0.165
ρ_η (F&S$_1$)	0.184	0.104	0.263	0.183	0.104	0.260
ρ_η (F&S$_2$)	0.108	0.019	0.195	0.106	0.018	0.193
ρ_η (S$_1$&S$_2$)	0.326	0.241	0.407	0.326	0.242	0.406

Table S37 shows the results of the model of interest alongside the results of a model which is identical except that it includes a fixed linear time trend for each dyad type. The estimates for the parameters that the models have in common (i.e. for all the parameters of the model of interest) do not greatly change between the two. In terms of the absolute change in point estimates, the largest changes are for the fixed intercepts, all with larger point estimates in the model with the time trend). The five parameters for which this change is greater than 0.1 are all fixed intercepts, and the fixed intercepts are all in the top thirteen parameters ordering by size of absolute change in point estimates (the other parameter which makes up the top thirteen in addition to the twelve fixed intercepts being the correlation between the innovations at the first snapshot for older and younger siblings, for which the point estimate in the model with the time trend is 0.071 smaller than in the model without a time trend). It is not surprising that the intercepts should show relatively more change, and (since, as we will see, the time trends tend to be negative) that

they should be estimated as larger in the model with the time trend, since they now represent not the average constructiveness but the average constructiveness at the first snapshot.

There are nine parameters with changes smaller than any of the intercepts (i.e., smaller than 0.044) but larger than 0.010: both freely estimated thresholds (both estimated as higher in the model with the time trend, 0.038 higher for τ_3 and 0.023 higher for τ_2), three of the autoregressive effects (all with smaller point estimates in the model with the time trend, by 0.030 for older siblings acting towards younger siblings, 0.013 for younger children acting towards mothers, and 0.012 for older chidren acting towards mothers), two of the generalised reciprocities (both with larger point estimates in the model with the time trend, by 0.024 for mothers and 0.015 for younger children), a dyad type variance (for older children acting towards mothers, 0.016 higher in the model with the time trend), and a dyadic reciprocity (for mothers and older children, 0.012 smaller in the model with the time trend). There are fifteen parameters with changes larger than 0.005 but no larger than 0.010; twenty-six parameters with changes larger than 0.001 but no larger than 0.005; eleven with changes of 0.001; and the remaining seven have changes smaller than 0.001.

As mentioned, the thresholds and fixed intercepts all have larger point estimates in the model with a time trend than in the model without a time trend. The largest change for a fixed intercept is 0.233 and the smallest is 0.044. The autoregressive and cross-lagged effects all have smaller point estimates in the model with a time trend than in the model without a time trend, with the exception of two autoregressive effects and two cross-lagged effects. Among autoregressive effects with smaller point estimates in the model with a time trend, the largest difference is 0.030 and the smallest difference is 0.001, while for those with larger point estimates in the model with a time trend, the larger difference is 0.001 and the smaller is less than this. Among cross-lagged effects with smaller point estimates in the model with a time trend, the largest difference is 0.010 and the smallest difference less than 0.001, while for those with larger point estimates in the model with a time trend, the larger difference is 0.002 and the smaller 0.001. With the exception of the family variance, which is estimated as 0.004 smaller in the model with a time trend, the variances are all estimated as larger in the model with a time trend. The differences range from 0.003 to 0.007 for the actor variances, 0.001 to 0.004 for the partner variances, and less than 0.001 to 0.016 for the dyad type variances. The generalised reciprocities also all have larger point estimates in the model with a time trend (ranging from 0.002 to 0.024 larger), as do all but two of the dyadic reciprocities, with point estimates of dyadic reciprocities being larger in the model with a time trend by between 0.001 and 0.005, and smaller by between 0.007 and 0.012. The correlations between the innovations at the first snapshot all have smaller point estimates in the model with a time trend, by between 0.001 and 0.071, and the correlations between the innovations at subsequent snapshots also all have smaller point estimates, by less than 0.001 to 0.006, except for one which has a point estimate larger by less than 0.001.

In terms of the absolute change as a percentage of the point estimate for the model with the time trend, there are a few quite large changes, but many of these are small changes in numeric terms to estimates which were already small. This applies to the largest change in these terms, which is a change of 227%, but represents a reduction of only 0.002 in the point estimate (of the cross-lagged effect for younger children acting towards fathers). Of the six changes of more than 20%, only two are absolute changes of more than 0.01: the correlation between the innovations at the first snapshot for older and younger siblings (a change of 80%, representing a reduction of 0.071), and the autoregressive effect for older siblings acting towards younger siblings (a change of 23%, representing a reduction of 0.030). Of the eleven changes of more than 10%, only four are absolute changes of more than 0.01: the generalised reciprocity for mothers (a change of 12%, representing an increase of 0.024), the fixed intercept for younger children acting towards mothers (also a change of 12%, representing an increase of 0.161), the fixed intercept for older siblings acting towards younger siblings (also a change of 12%, representing an increase of 0.233), and the fixed intercept for older children acting towards mothers (a change of 11%, representing an increase of 0.181). Of the remaining changes, eight are greater than 5% but less than 10%, twenty-one are greater than 2.5% but less than 5%, twenty-one are greater than 1% but less than 2.5%, and the remaining fourteen are less than 1%.

The widths of the 95% credible intervals show even less change than the point estimates. Again the largest changes in absolute terms are for the fixed intercepts (this time with no other parameter intervening when the parameters are ranked in order of the magnitude of the difference), with the intervals for all of these being wider in the model with a time trend than in the model without a time trend, and the largest difference being 0.071 (for fathers acting

towards mothers) and the smallest 0.041 (for mothers acting towards younger children). Besides the fixed intercepts, only six parameters have absolute changes of more than 0.010: one of the thresholds (τ_2, wider in the model with a time trend), two of the correlations between the innovations at the first snapshot (for younger and older siblings, wider in the model with a time trend; and for mothers and fathers, narrower in the model with a time trend), a generalised reciprocity (for younger children, narrower in the model with a time trend), and two dyadic reciprocities (for younger and older siblings, and for fathers and younger children, both wider in the model with a time trend). There are eleven parameters with changes of greater than 0.005 but less than 0.010 (six of which have narrower intervals in the model with the time trend and five wider); twenty-three with changes of greater than 0.001 but no greater than 0.005 (five of which have narrower intervals in the model with the time trend and eighteen wider); and the remaining twenty-nine have changes of 0.001 or less (fourteen of which have narrower intervals in the model with the time trend and fifteen wider). The changes are also smaller than for the point estimates in terms of the percentage of the width of the interval in the model with a time trend that the absolute change represents. The largest change, for τ_2, is 39% (corresponding to an absolute difference of 0.033), and then the next largest are all the fixed intercepts, ranging from 13% (for mothers acting towards younger children) to 22% (for older children acting towards fathers). The remaining parameters all have changes less than 10%, with only two being larger than 5% (the family variance, at 8%, and the correlation between the innovations at the first snapshot for younger and older siblings, at 6%). There are ten parameters with changes of less than 5% but greater than 2.5%; twenty-one with changes of less than 2.5% but greater than 1%; and the remaining thirty-five parameters have changes of less than 1%.

The pattern of significance is the same for the model with and without a time trend for the parameters common to both, so in particular, the same two autoregressive effects are found non-significant, the same cross-lagged effect is found significant, the generalised reciprocity is found significant, the same dyadic reciprocity is found significant, the same three correlations between innovations at the first snapshot are found significant, and the same correlation between innovations at subsequent snapshots is found non-significant.

Table S38: Differences between parameters in ordinal DSRMs with and without time trend

	Mean	S.D.	Quantiles				
			2.5%	25%	50%	75%	97.5%
τ_1	0.000	0.000	0.000	0.000	0.000	0.000	0.000
τ_2	0.023	0.030	−0.029	0.006	0.020	0.039	0.082
τ_3	0.038	0.044	−0.058	0.009	0.045	0.068	0.112
μ (M▸F)	0.095	0.104	−0.110	0.025	0.094	0.165	0.300
μ (F▸M)	0.077	0.107	−0.135	0.005	0.077	0.149	0.288
μ (M▸S_1)	0.065	0.107	−0.146	−0.007	0.066	0.138	0.270
μ (S_1▸M)	0.161	0.096	−0.025	0.096	0.161	0.226	0.350
μ (M▸S_2)	0.057	0.101	−0.140	−0.011	0.058	0.125	0.256
μ (S_2▸M)	0.181	0.094	−0.002	0.117	0.180	0.244	0.367
μ (F▸S_1)	0.082	0.118	−0.150	0.003	0.083	0.161	0.317
μ (S_1▸F)	0.066	0.098	−0.127	−0.000	0.065	0.132	0.258
μ (F▸S_2)	0.121	0.109	−0.091	0.047	0.121	0.195	0.333
μ (S_2▸F)	0.044	0.093	−0.136	−0.019	0.044	0.108	0.225
μ (S_1▸S_2)	0.105	0.088	−0.069	0.045	0.104	0.164	0.282
μ (S_2▸S_1)	0.233	0.095	0.046	0.169	0.232	0.296	0.419
ϕ_1 (M▸F)	−0.005	0.067	−0.137	−0.049	−0.005	0.040	0.127
ϕ_1 (F▸M)	−0.003	0.066	−0.131	−0.048	−0.003	0.042	0.127
ϕ_1 (M▸S_1)	−0.002	0.048	−0.095	−0.034	−0.002	0.030	0.091
ϕ_1 (S_1▸M)	−0.013	0.053	−0.116	−0.049	−0.014	0.022	0.091
ϕ_1 (M▸S_2)	−0.001	0.049	−0.097	−0.034	−0.002	0.032	0.096
ϕ_1 (S_2▸M)	−0.012	0.050	−0.109	−0.046	−0.012	0.022	0.088
ϕ_1 (F▸S_1)	−0.004	0.065	−0.132	−0.048	−0.004	0.039	0.124

(cont.)

(cont.)

	Mean	S.D.	Quantiles				
			2.5%	25%	50%	75%	97.5%
ϕ_1 (S_1▸F)	0.001	0.063	−0.124	−0.040	0.002	0.043	0.124
ϕ_1 (F▸S_2)	−0.007	0.062	−0.128	−0.049	−0.007	0.035	0.112
ϕ_1 (S_2▸F)	0.000	0.064	−0.126	−0.044	−0.000	0.044	0.126
ϕ_1 (S_1▸S_2)	−0.000	0.065	−0.130	−0.044	0.000	0.044	0.128
ϕ_1 (S_2▸S_1)	−0.030	0.070	−0.167	−0.078	−0.030	0.017	0.105
ϕ_2 (M▸F)	−0.006	0.064	−0.133	−0.049	−0.006	0.038	0.119
ϕ_2 (F▸M)	−0.003	0.061	−0.123	−0.043	−0.003	0.038	0.117
ϕ_2 (M▸S_1)	−0.010	0.059	−0.126	−0.049	−0.009	0.030	0.104
ϕ_2 (S_1▸M)	−0.002	0.040	−0.081	−0.029	−0.002	0.025	0.078
ϕ_2 (M▸S_2)	0.001	0.051	−0.100	−0.033	0.001	0.035	0.102
ϕ_2 (S_2▸M)	0.002	0.044	−0.085	−0.028	0.002	0.031	0.087
ϕ_2 (F▸S_1)	−0.004	0.070	−0.141	−0.050	−0.003	0.044	0.133
ϕ_2 (S_1▸F)	−0.002	0.054	−0.108	−0.037	−0.001	0.035	0.103
ϕ_2 (F▸S_2)	−0.000	0.070	−0.138	−0.048	0.000	0.048	0.138
ϕ_2 (S_2▸F)	−0.000	0.051	−0.101	−0.035	−0.000	0.034	0.100
ϕ_2 (S_1▸S_2)	−0.005	0.059	−0.121	−0.044	−0.005	0.035	0.111
ϕ_2 (S_2▸S_1)	−0.002	0.068	−0.136	−0.048	−0.002	0.044	0.132
σ_f^2	−0.004	0.042	−0.093	−0.027	−0.002	0.018	0.083
σ_a^2 (M)	0.007	0.064	−0.118	−0.035	0.007	0.050	0.132
σ_a^2 (F)	0.008	0.060	−0.110	−0.032	0.008	0.048	0.127
σ_a^2 (S_1)	0.005	0.041	−0.075	−0.023	0.004	0.032	0.085
σ_a^2 (S_2)	0.003	0.036	−0.068	−0.021	0.003	0.027	0.073
σ_p^2 (M)	0.001	0.030	−0.060	−0.019	0.001	0.020	0.061
σ_p^2 (F)	0.001	0.021	−0.041	−0.013	0.000	0.014	0.042
σ_p^2 (S_1)	0.004	0.042	−0.079	−0.024	0.004	0.032	0.087
σ_p^2 (S_2)	0.001	0.031	−0.060	−0.019	0.001	0.022	0.063
σ_d^2 (M▸F)	0.003	0.068	−0.130	−0.041	0.003	0.047	0.140
σ_d^2 (F▸M)	0.002	0.054	−0.104	−0.032	0.002	0.037	0.111
σ_d^2 (M▸S_1)	0.010	0.120	−0.228	−0.070	0.009	0.089	0.248
σ_d^2 (S_1▸M)	0.007	0.102	−0.195	−0.061	0.006	0.075	0.208
σ_d^2 (M▸S_2)	0.006	0.113	−0.214	−0.070	0.007	0.082	0.229
σ_d^2 (S_2▸M)	0.016	0.098	−0.177	−0.049	0.016	0.081	0.207
σ_d^2 (F▸S_1)	0.008	0.113	−0.213	−0.067	0.007	0.081	0.235
σ_d^2 (S_1▸F)	0.001	0.052	−0.103	−0.032	0.001	0.035	0.105
σ_d^2 (F▸S_2)	0.010	0.092	−0.171	−0.050	0.010	0.070	0.194
σ_d^2 (S_2▸F)	0.002	0.048	−0.094	−0.029	0.002	0.032	0.098
σ_d^2 (S_1▸S_2)	0.000	0.040	−0.080	−0.025	0.000	0.026	0.080
σ_d^2 (S_2▸S_1)	0.009	0.081	−0.152	−0.043	0.009	0.062	0.168
ρ_{ap} (M)	0.024	0.242	−0.451	−0.139	0.026	0.187	0.499
ρ_{ap} (F)	0.010	0.248	−0.473	−0.161	0.009	0.178	0.498
ρ_{ap} (S_1)	0.015	0.223	−0.424	−0.135	0.015	0.163	0.455
ρ_{ap} (S_2)	0.002	0.215	−0.420	−0.140	0.004	0.146	0.424
ρ_d (M&F)	0.005	0.291	−0.562	−0.193	0.004	0.203	0.569
ρ_d (M&S_1)	0.004	0.163	−0.314	−0.106	0.003	0.113	0.328
ρ_d (M&S_2)	−0.012	0.195	−0.396	−0.144	−0.011	0.118	0.373
ρ_d (F&S_1)	0.001	0.298	−0.578	−0.204	0.002	0.205	0.580
ρ_d (F&S_2)	0.004	0.287	−0.552	−0.193	0.003	0.198	0.565
ρ_d (S_1&S_2)	−0.007	0.262	−0.527	−0.180	−0.008	0.170	0.508

(cont.)

(cont.)

	Mean	S.D.	Quantiles				
			2.5%	25%	50%	75%	97.5%
ρ_{e1} (M&F)	−0.004	0.220	−0.434	−0.153	−0.003	0.145	0.426
ρ_{e1} (M&S_1)	−0.004	0.116	−0.232	−0.083	−0.004	0.074	0.223
ρ_{e1} (M&S_2)	−0.001	0.124	−0.243	−0.086	−0.002	0.083	0.241
ρ_{e1} (F&S_1)	−0.006	0.138	−0.275	−0.099	−0.006	0.087	0.267
ρ_{e1} (F&S_2)	−0.001	0.154	−0.301	−0.106	−0.003	0.102	0.302
ρ_{e1} (S_1&S_2)	−0.071	0.176	−0.417	−0.189	−0.070	0.048	0.276
ρ_{η} (M&F)	−0.006	0.082	−0.165	−0.061	−0.007	0.049	0.155
ρ_{η} (M&S_1)	−0.004	0.043	−0.089	−0.034	−0.004	0.025	0.080
ρ_{η} (M&S_2)	0.000	0.044	−0.087	−0.030	0.000	0.030	0.087
ρ_{η} (F&S_1)	−0.001	0.057	−0.113	−0.040	−0.001	0.038	0.111
ρ_{η} (F&S_2)	−0.002	0.063	−0.124	−0.044	−0.002	0.041	0.122
ρ_{η} (S_1&S_2)	−0.000	0.059	−0.117	−0.040	0.000	0.040	0.115

Table S38 shows the result of subtracting each parameter in the model without a time trend from the corresponding parameter in the model with a time trend at each iteration, then summarising the resulting chain of differences, similarly to what we did to look at the difference between the fixed intercepts, autoregressive effects, cross-lagged effects, and other parameters in the model of interest. The mean differences are all identical to the differences in point estimates (to 3d.p.). There is a significant difference for only one parameter: the fixed intercept for older siblings acting towards younger siblings (the same parameter for which we found the largest difference in point estimates). The 95% credible interval for this difference is 0.046 to 0.419.

Returning to Table S37, the point estimates of the time trends are all negative, suggesting participants' behaviour may tend to get less constructive as the exercise proceeds no matter what their family role is and who they are interacting with. However, the time trends are only significant for five dyad types, so we can definitely conclude that behaviour becomes less constructive over time only for younger and older children acting towards mothers, fathers acting towards older children, and younger and older siblings acting towards each other. These significant time trends range from −0.020 (for fathers acting towards older children) to −0.057 (for older siblings acting towards younger siblings), or in other words an expected 0.3 to 0.8 lower at the last snapshot compared to the first.

Figure S1 shows the average trajectory over time for each dyad type, calculated as $\mu_{ij} + \alpha_{1ij}t$, using the point estimates of μ_{ij} and α_{1ij}. It is immediately clear that the trajectory for older siblings acting towards younger siblings is markedly steeper than those for other dyad types, and also apparent that the trajectory for older children acting towards fathers is markedly flatter than those for other dyad types. (It is also readily apparent that constructiveness is higher across the whole period of observation for dyad types involving a parent as the actor than for dyad types involving a child as the actor.)

Figures S2 to S13 show for each dyad type a comparison between the average trajectory in the model with a time trend and the average trajectory in the model without a time trend (the latter, of course, being completely flat in every case). For the model with the time trend, we calculate the average trajectory at every iteration using the intercept and time trend for that dyad type at that iteration, then summarise across iterations, with the lines plotting the median at each snapshot and the coloured regions plotting the interval between the 2.5th and 97.5th percentiles at each snapshot. For the model without the time trend, the idea is the same, but things are simpler, as instead of calculating the average trajectory at each snapshot, we simply plot at every snapshot the median estimate of the fixed intercept and the upper and lower limits of its 95% credible interval. The thresholds shown are for the model with a time trend.

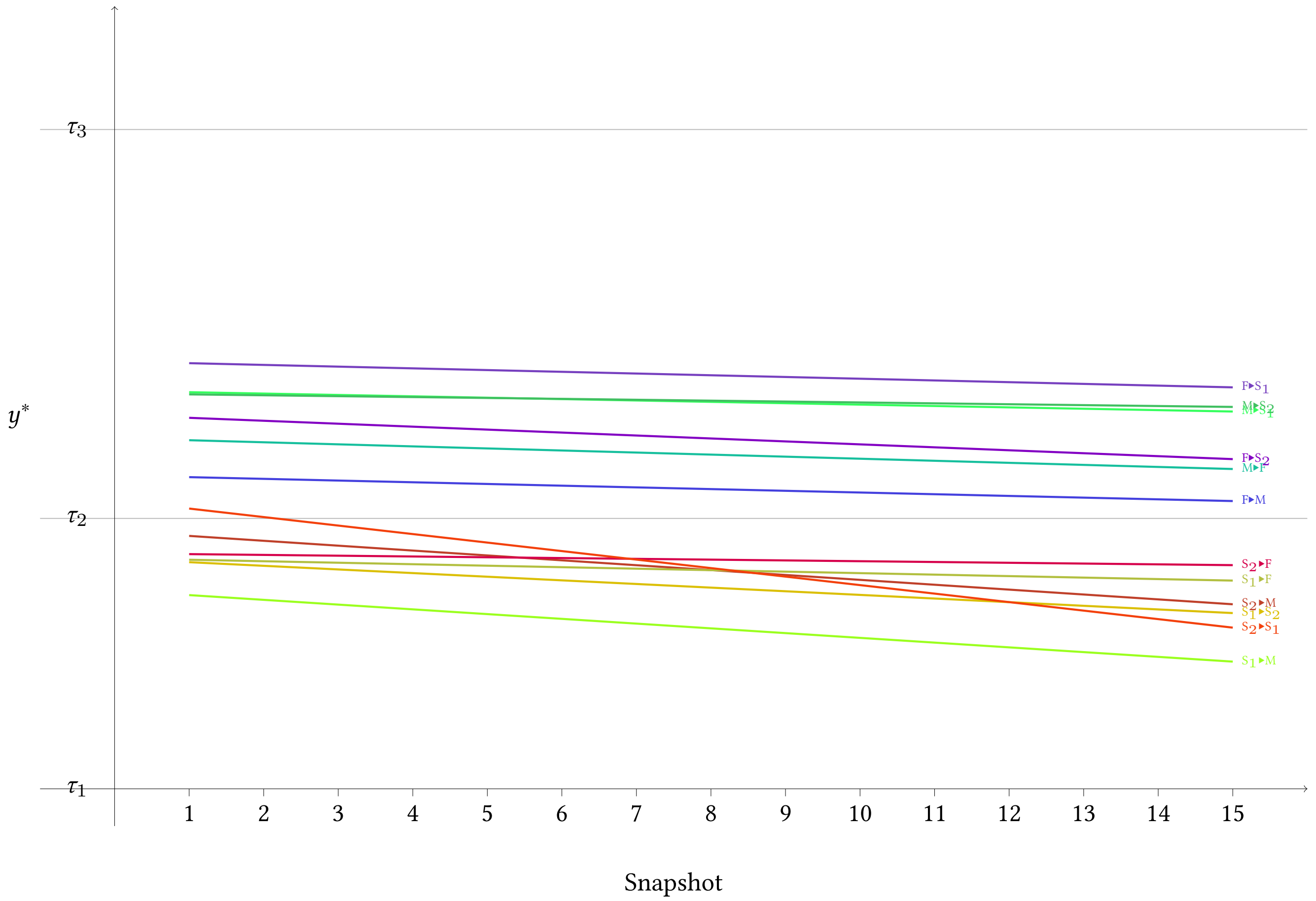

Figure S1: Predicted constructiveness over time for each dyad type

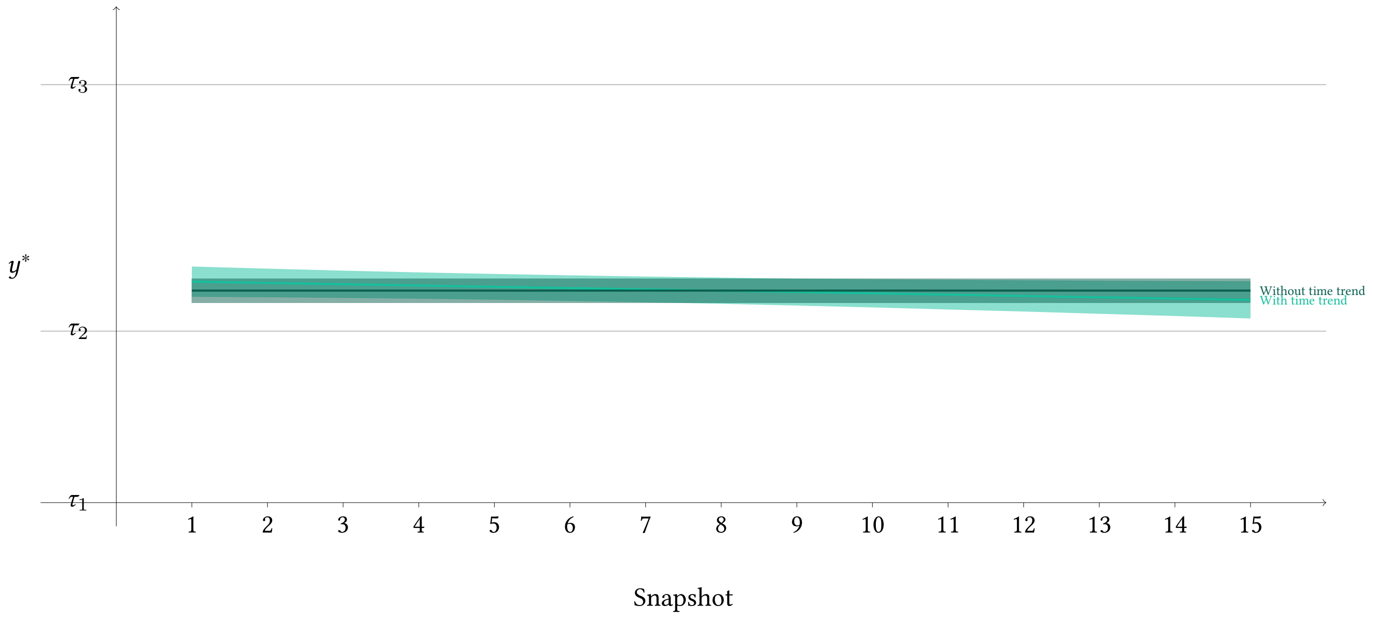

Figure S2: Predicted constructiveness over time for mothers acting towards fathers in the models with and without a time trend

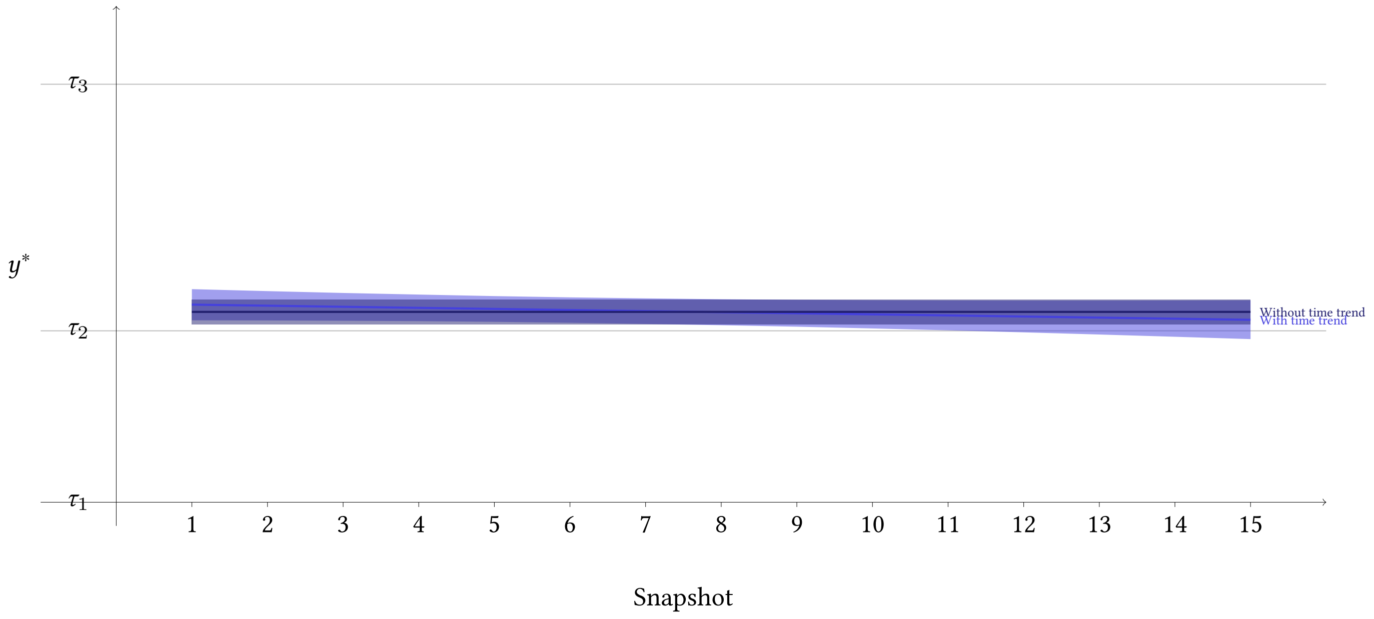

Figure S3: Predicted constructiveness over time for fathers acting towards mothers in the models with and without a time trend

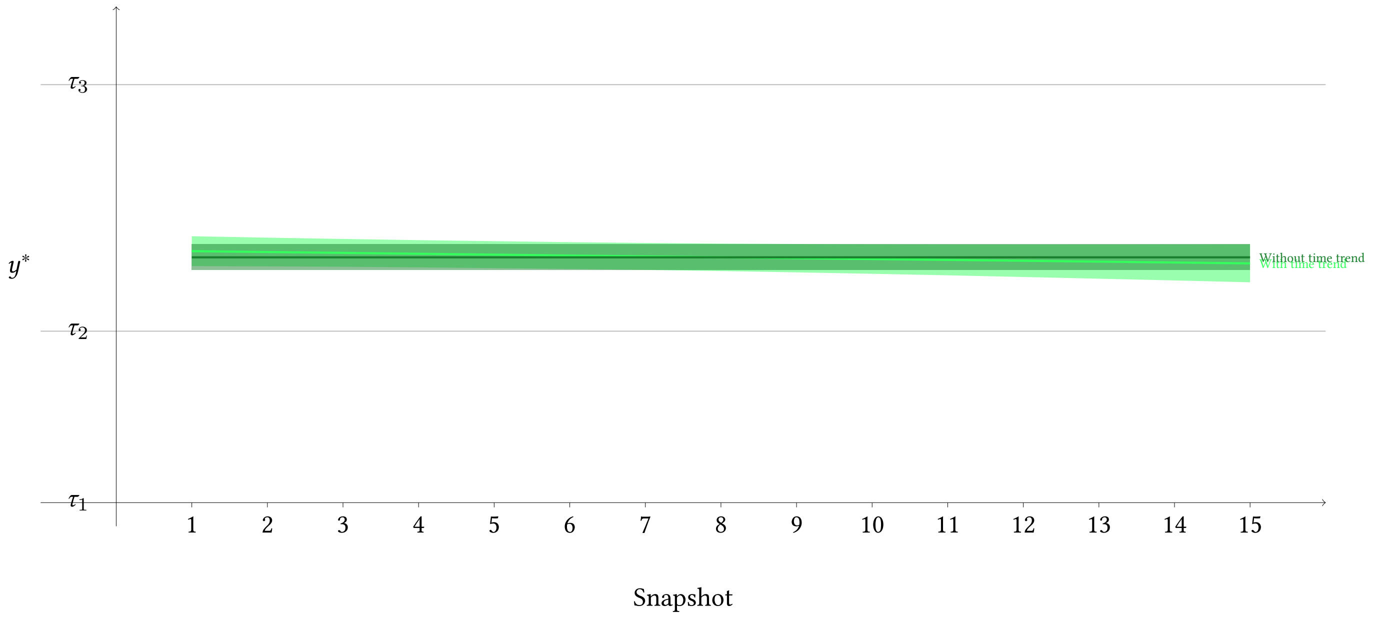

Figure S4: Predicted constructiveness over time for mothers acting towards younger children in the models with and without a time trend

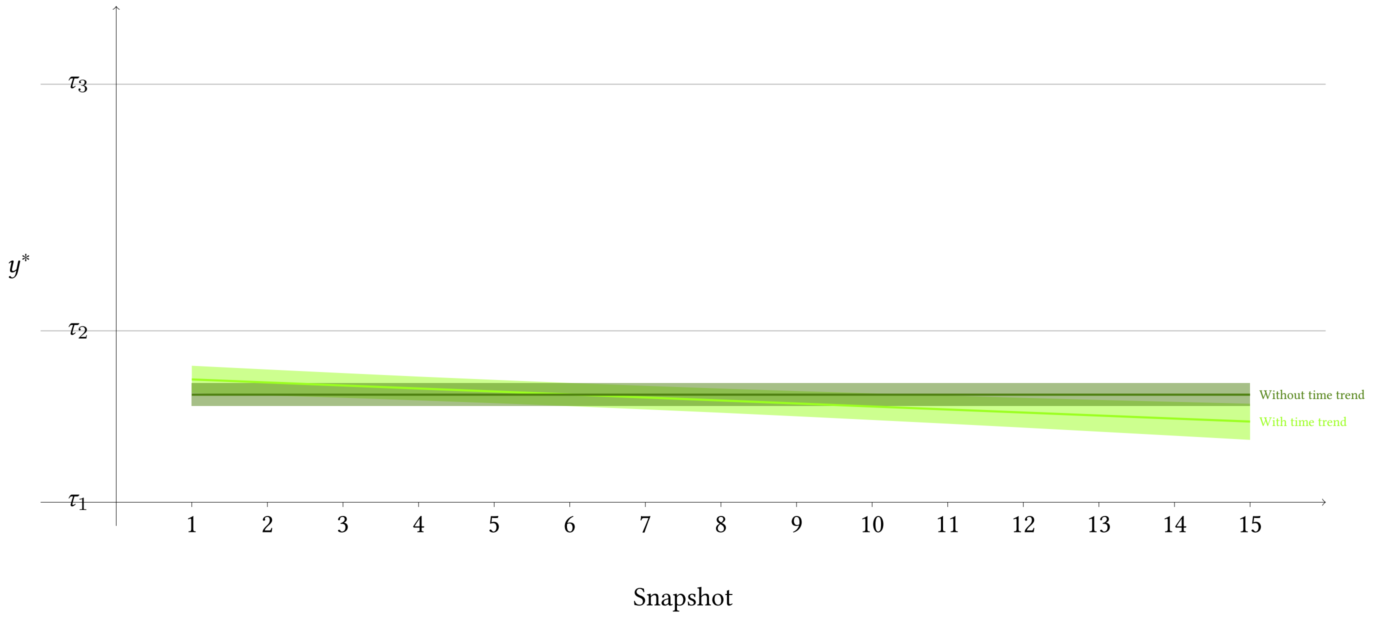

Figure S5: Predicted constructiveness over time for younger children acting towards mothers in the models with and without a time trend

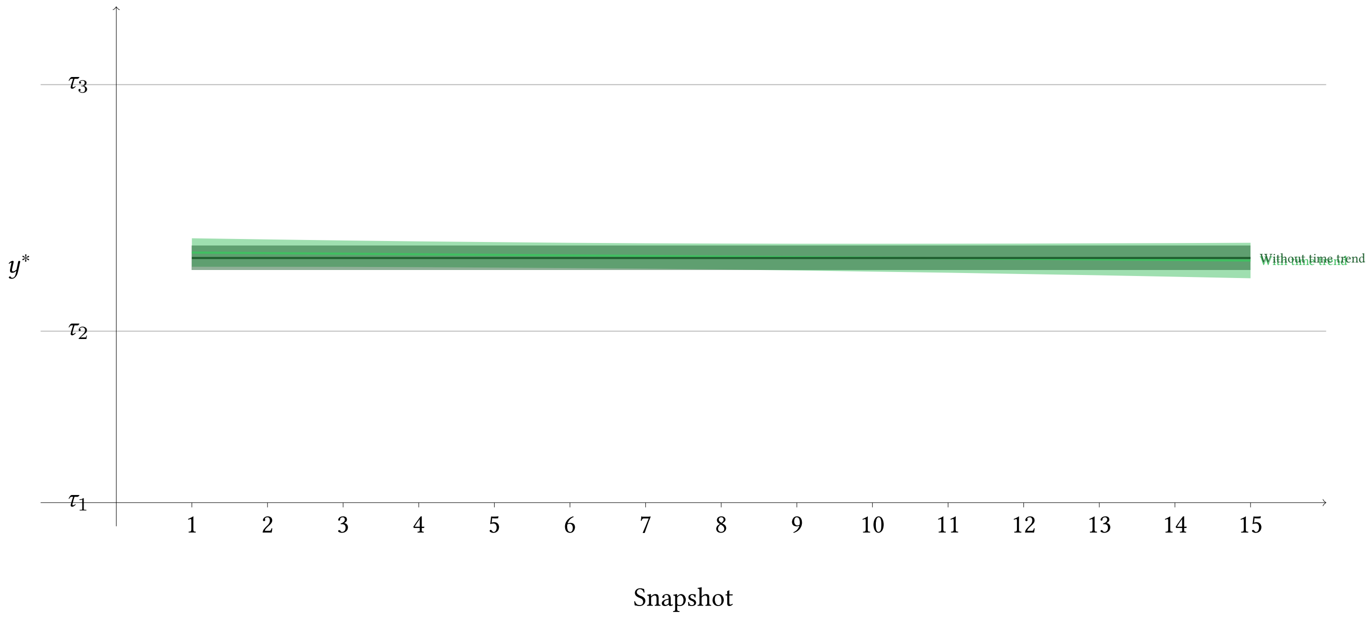

Figure S6: Predicted constructiveness over time for mothers acting towards older children in the models with and without a time trend

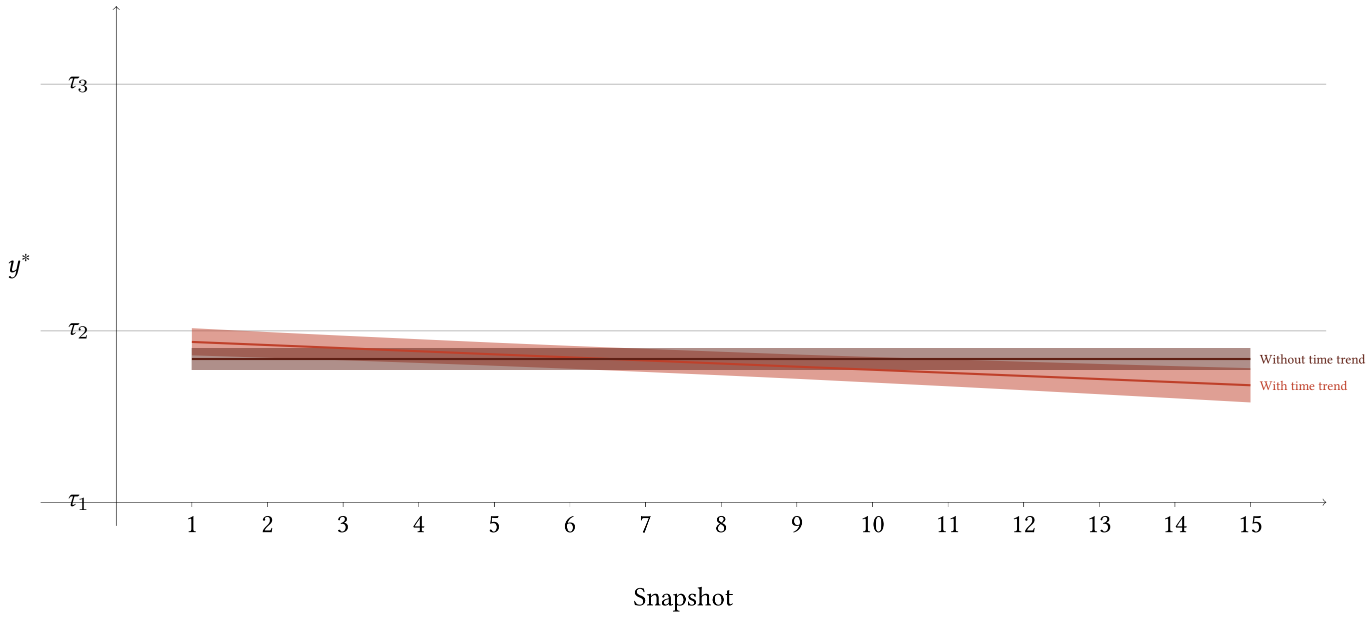

Figure S7: Predicted constructiveness over time for older children acting towards mothers in the models with and without a time trend

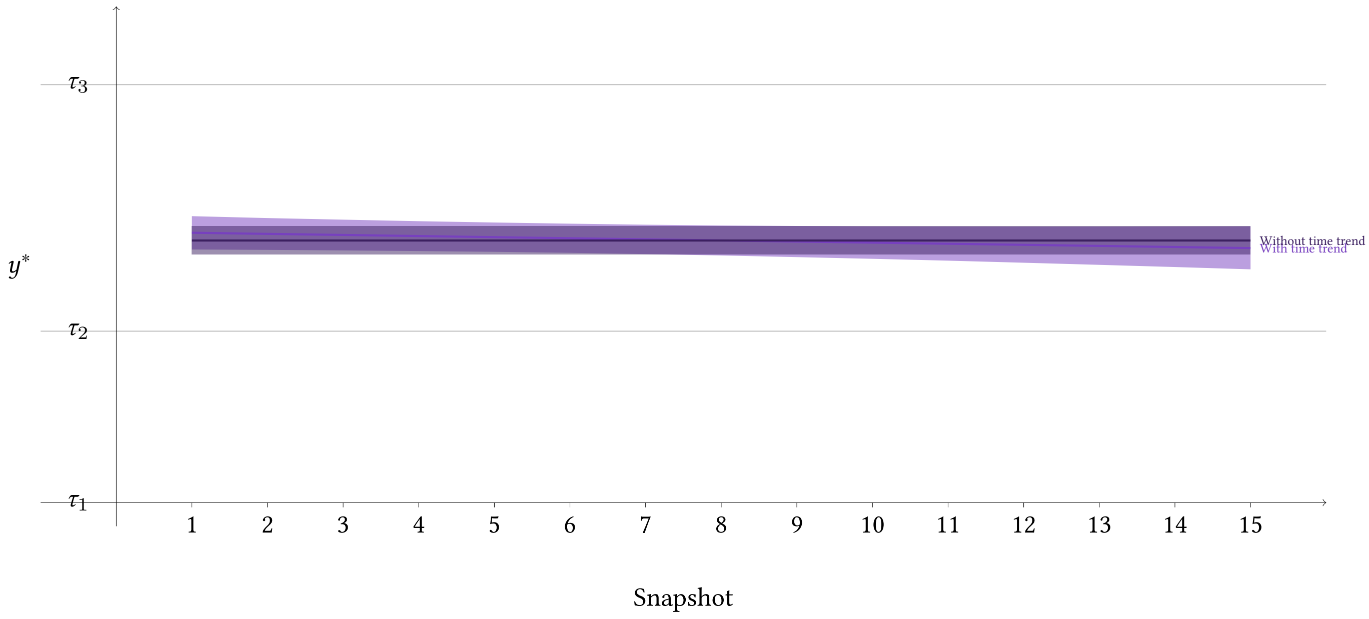

Figure S8: Predicted constructiveness over time for fathers acting towards younger children in the models with and without a time trend

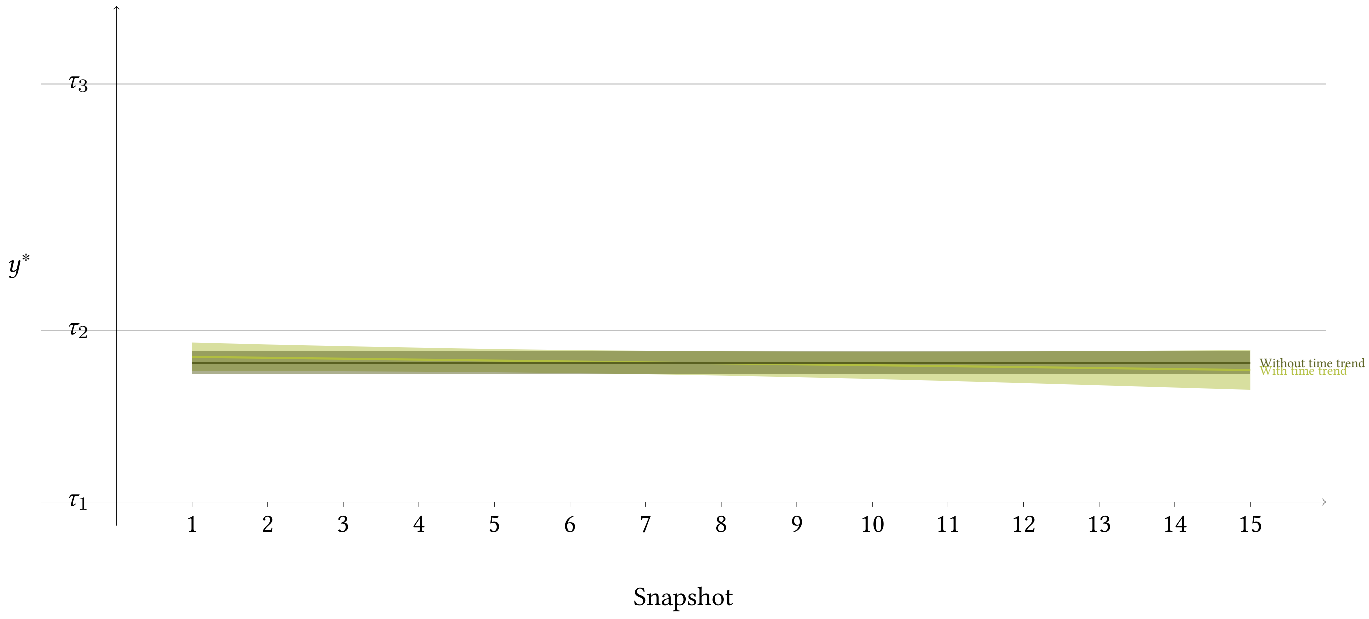

Figure S9: Predicted constructiveness over time for younger children acting towards fathers in the models with and without a time trend

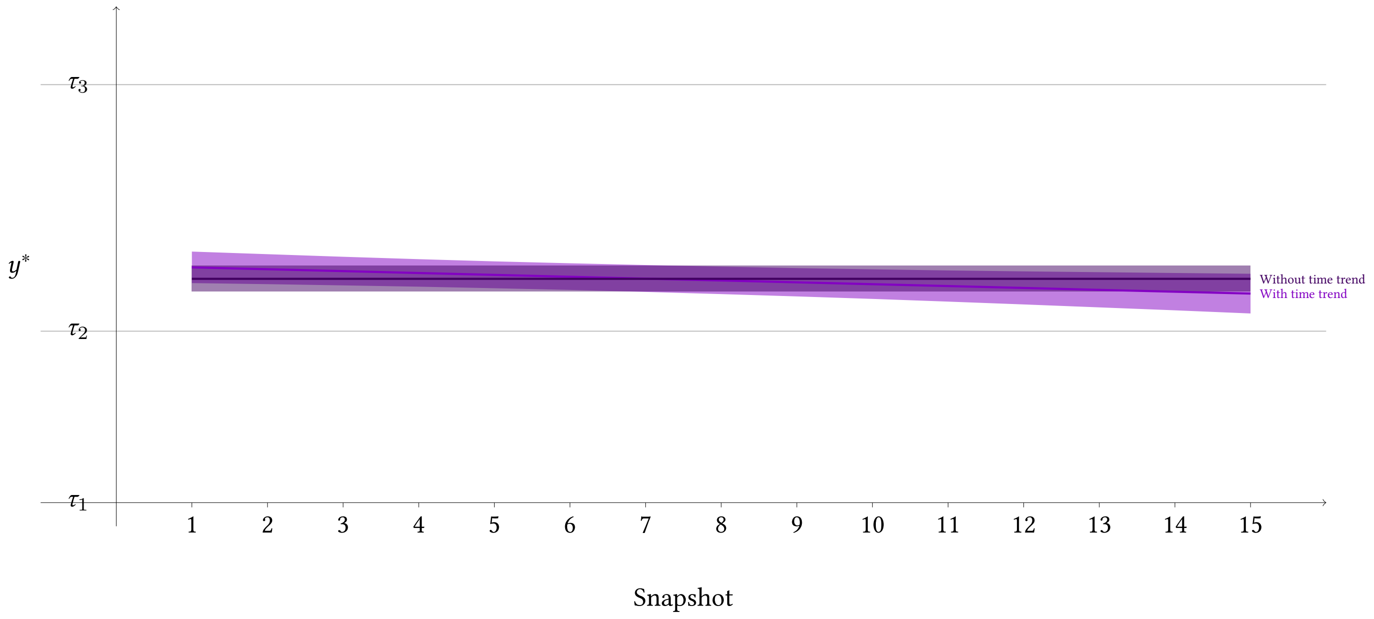

Figure S10: Predicted constructiveness over time for fathers acting towards older children in the models with and without a time trend

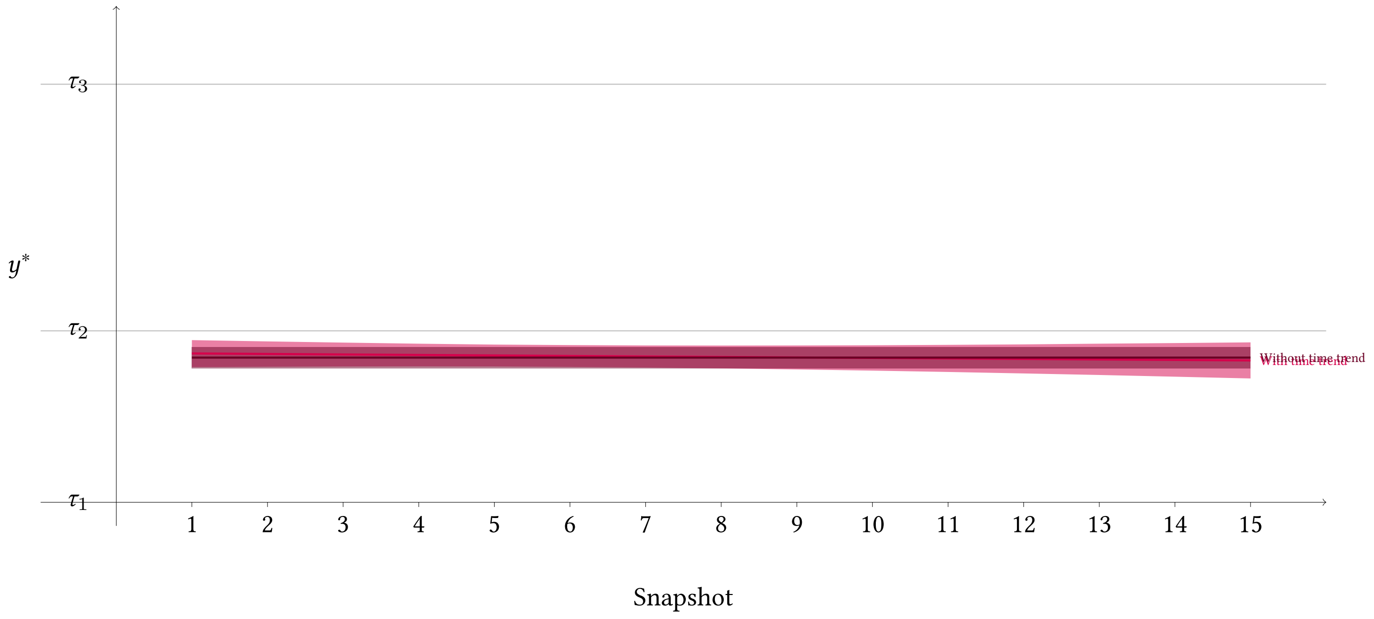

Figure S11: Predicted constructiveness over time for older children acting towards fathers in the models with and without a time trend

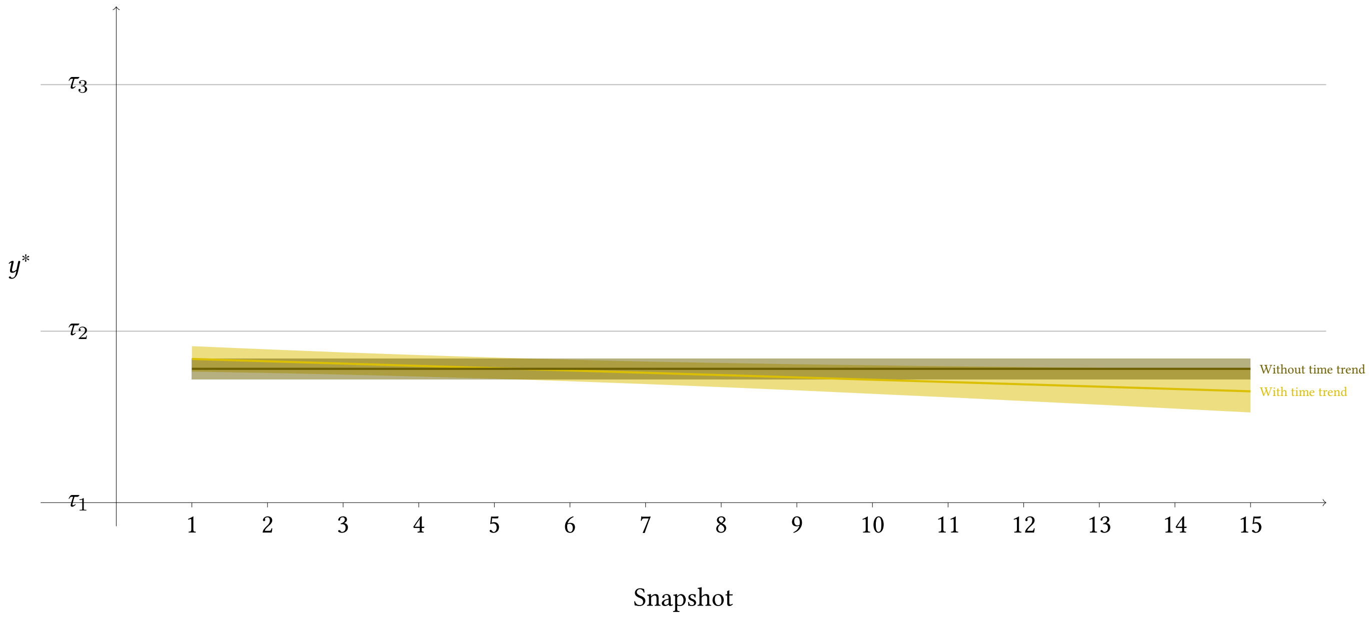

Figure S12: Predicted constructiveness over time for younger siblings acting towards older siblings in the models with and without a time trend

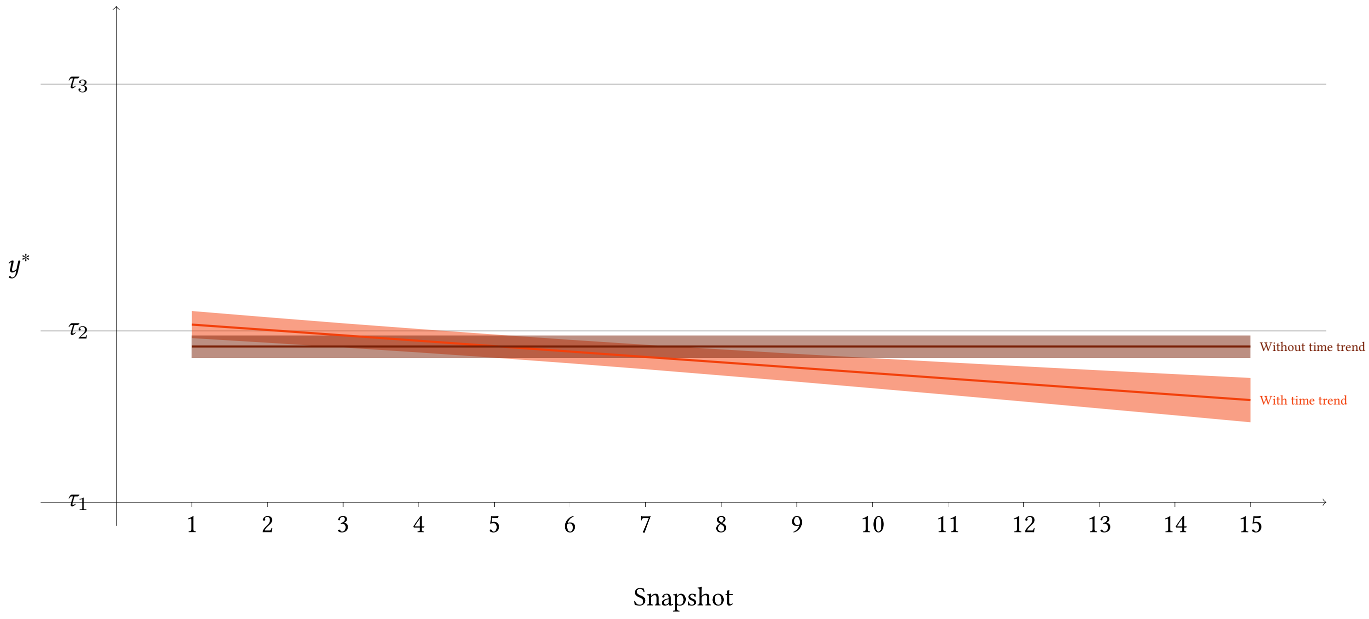

Figure S13: Predicted constructiveness over time for older siblings acting towards younger siblings in the models with and without a time trend

It can be seen that for most dyad types there is considerable overlap throughout the observation period between the trajectories from the two models, although for others (particularly olders siblings acting towards younger siblings) this is only the case for a few snapshots somewhere in the middle of the observation period, with more separation between the trajectories at the start and end of the period.

References

Browne, D. T., Wade, M., Plamondon, A., Leckie, G., Perlman, M., Madigan, S. and Jenkins, J. M. (2018) Child and contextual effects in the emergence of differential maternal sensitivity across siblings. *Developmental Psychology*, **54**, 1265–1276.

Meunier, J. C., Boyle, M., O'Connor, T. G. and Jenkins, J. M. (2013) Multilevel mediation: Cumulative contextual risk, maternal differential treatment, and children's differential behaviour within families. *Child Development*, **84**, 1594–1615.

Pillinger, R., Steele, F., Leckie, G. and Jenkins, J. (Submitted) A dynamic social relations model for multi-group longitudinal dyadic data with continuous or ordinal responses. *Journal of the Royal Statistical Society Series A*.
